# Introgression of Eastern Chinese and Southern Chinese haplotypes contributes to the improvement of fertility and immunity in European modern pigs

**DOI:** 10.1093/gigascience/giaa014

**Published:** 2020-03-06

**Authors:** Hao Chen, Min Huang, Bin Yang, Zhongping Wu, Zheng Deng, Yong Hou, Jun Ren, Lusheng Huang

**Affiliations:** State Key Laboratory of Pig Genetic Improvement and Production Technology, Jiangxi Agricultural University, Nanchang 330045, P. R. China

**Keywords:** French Large White, disease resistance, reproductive performance, AHR, artificial introgression, gene

## Abstract

**Background:**

Pigs were domesticated independently from European and Asian wild boars nearly 10,000 years ago. Chinese indigenous pigs have been historically introduced to improve Europe local pigs. However, the geographic origin and biological functions of introgressed Chinese genes in modern European pig breeds remain largely unknown.

**Results:**

Here we explored whole-genome sequencing data from 266 Eurasian wild boars and domestic pigs to produce a fine-scale map of introgression between French Large White (FLW) and Chinese pigs. We show that FLW pigs had historical admixture with both Southern Chinese (SCN) and Eastern Chinese (ECN) pigs ∼200–300 years ago. Moreover, a set of SCN haplotypes was shown to be beneficial for improving disease resistance and ECN haplotypes are favorable for improved reproductive performance in FLW pigs. In addition, we confirm human-mediated introgression events at the *AHR* locus, at which the haplotype of most likely ECN origin contributes to increased fertility of FLW pigs.

**Conclusions:**

This study advances our understanding of the breeding history of global domestic pigs and highlights the importance of artificial introgression in the formation of phenotypic characteristics in domestic animals.

## Introduction

Integrated genomic and archaeological evidence has illuminated the fact that the wild boar (*Sus scrofa*) originated in the islands of southeast Asia ∼5 million years ago and then dispersed throughout Eurasia. Approximately 1 million years ago, geographic isolation caused by glacial events hampered the continuous gene flow among Eurasian wild boars, causing European and Asian wild boars to differentiate from each other [1–4]. Roughly 10,000 years ago, European and Asian wild boars were domesticated independently in the Near East and China, respectively [3, 5, 6]. After long-term artificial selection and natural selection, abundant genetic resources of domestic pigs appeared in China, accounting for approximately one-third of global breeds [7, 8]. Chinese pigs are distributed in diverse geographic regions and have different breed features. For example, Erhualian (EHL) and Meishan pigs in East China are known for their prolificacy, with a litter size of >15, and for their thick skin. Luchuan (LUC) and Bama pigs in South China have inferior reproductive performance (8–10 piglets per parity), thin skin, and excellent heat resistance [7]. These pig breeds not only play a critical role in the Chinese pig industry but also have contributed to the development of international commercial breeds, such as the Large White (LW) [9, 10].

Chinese pigs were introduced to Europe mainly during 3 historical periods [7]. From 1685 to 1757, the Qing Dynasty set up 4 foreign trade ports: 2 in East China (Shanghai and Ningbo) and 2 (Zhangzhou and Guangzhou) in South China. Europe (especially England) had frequent trade with China through these 4 ports, mainly via the East India Company. This raises the possibility that Eastern Chinese (ECN) and Southern Chinese (SCN) pigs may have been transported to European countries during this period. From 1757 to 1841, only the Guangzhou port in South China was permitted access to foreign trade, and a ban was imposed on maritime trade or intercourse with foreign countries in 1757. It is well documented that SCN pigs had been introduced to England for the hybridization of local pigs during this period, contributing to the formation of Berkshire [9] and LW pigs [10]. In 1978, the Chinese government launched the reform and open-door policy. Since then, ECN pigs, including Meishan, Jinhua, and Jiaxing Black, have been introduced into France, America, and Japan for the development of prolific synthetic lines [7].

Recently, whole-genome resequencing analysis has confirmed the human-mediated translocation of Chinese pigs into Europe that provided genetic variations for the selective breeding of modern commercial LW pigs [11]. However, it remains unknown whether SCN or ECN pigs or both were introduced to Europe, because previous studies used a limited number of Chinese pigs from different locations as a whole population. French Large White (FLW) pigs are known for their excellent reproductive performance. A remarkable genetic improvement of litter size has been witnessed in FLW pigs over the past decades, but the molecular mechanisms underlying the fecundity remain unclear, although the fecundity is speculated to be related to the recent introgression of highly prolific Chinese pigs such as ECN pigs [7]. Further studies are required to test this speculation.

In this study, we explored whole-genome sequencing data of 266 Eurasian pigs to show that both SCN and ECN haplotypes were introgressed into LW pigs ∼200–300 years ago. Some of the introgressed haplotypes have been under preferential selection to improve fertility and immunity in FLW pigs. For instance, the prolificacy-associated *AHR* haplotype was most likely introgressed from ECN pigs to FLW pigs through human-driven transportation. These findings advance our understanding of the breeding history and genetic mechanisms underlying breed characteristics of global domestic pigs.

## Results

### Whole-genome sequencing data

We obtained whole-genome sequencing data of 266 animals from 25 populations (Supplementary Table S1), including 36 highly prolific FLW pigs from the nucleus populations of 2 breeding companies. The 36 pigs were selected on the basis of their total number born (TNB) of >19 piglets and distant genetic relationship between each individual (Supplementary Fig. S1). High-depth resequencing was conducted on a Hiseq 2000 or 2500 sequencer (Illumina, San Diego, California, USA data (see Methods), we called 32.7 million single-nucleotide polymorphisms (SNPs) from the 266 individuals. For the 28 LW pigs whose sequence data were retrieved from the public NCBI database (see Methods), we used the Illumina Porcine SNP60 chip [12] data set to identify their origin. We demonstrated that 14 individuals belonged to the American Large White (ALW) lineage, and the other 14 individuals belonged to the Dutch Large White (NLW) line (Supplementary Fig. S2).

### Genetic differentiation between SCN and ECN pigs

**Figure 1:**
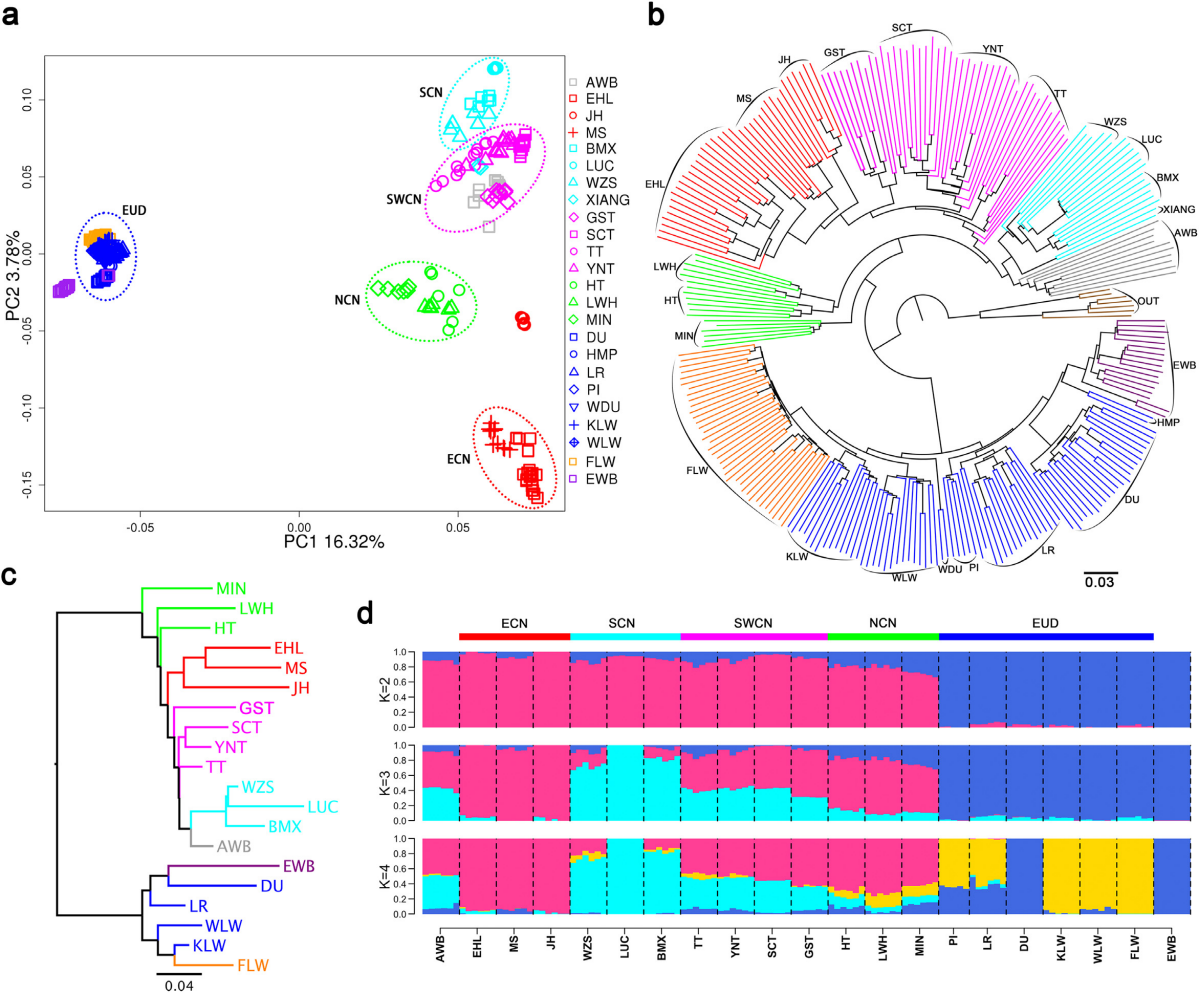
Population relationship and structure. (**a**) Principal component analysis of Chinese and European pigs. ECN, East Chinese pig; EUD, European domestic pig; NCN, North Chinese pig; SCN, South Chinese pig; SWCN, Southwest Chinese pig. (**b**) Neighbor-joining (NJ) tree based on an identity-by-state matrix among individuals. (**c**) NJ tree based on an F_ST_ matrix between populations. (**d**) Population structure of Chinese and European pigs revealed by ADMIXTURE analysis. AWB: Asian wild boar; BMX: Bamaxiang pig; DU: Duroc; EHL: Erhualian pig; EWB: European wild boar; FLW: French Large White pig; GST: Tibetan pig (gansu); HMP: Hampshire; HT: Hetao pig; JH: Jinhua pig; KLW: Korea Large White pig; LR: Landrace; LUC: Luchuan pig; LWH: Laiwu pig; MIN: Min pig; MS: Meishan pig; OUT: outgroup; PI: Pietrain; SCT: Tibetan pig (Sichuan); TT: Tibetan pig (Tibet); WDU: White Duroc; WLW: Dutch Large White pig; WZS: Wuzhishan pig; XIANG: Xiang pig; YNT: Tibetan pig (Yunnan).

Eurasian wild boars began to differentiate as early as ∼1 million years ago [2, 3], and Chinese and European wild boars were independently domesticated ∼10,000 years ago [1, 3]. The remarkable genetic differentiation between Chinese and Western pigs was reflected in the results from principal component analysis (PCA), phylogenetic analysis, and admixture analysis (Fig. 1). In our PCA analysis, the first principal component (PC1) accounted for 16.32% of the total eigenvalue, which clearly separated the Chinese pig from the Western pig. The second principal component (PC2) showed the differentiation among Chinese pigs, especially between SCN and ECN pigs (PC2 = 3.78%; Fig. 1a). In a neighbor-joining (NJ) tree between individuals (Fig. 1b) and populations (Fig. 1c), Chinese and Western pigs defined 2 separate clades. For Chinese domestic pigs, SCN and ECN pigs formed 2 different branches. The clustering pattern was similar to the maximum likelihood tree revealed with TreeMix analysis, in which 2 Sumatras wild boars, 1 *Sus barbatus*, 1 *Sus verrucosus*, 1 *Sus cebifrons*, 1 *Sus celebensis*, and 1 *Phacochoerus africanus* were treated as an outgroup (OUT), and the interpretation of the maximum likelihood tree reached 99.9% (Supplementary Fig. S3). In an admixture analysis, Chinese pigs and European pigs showed 2 distinct ancestral lineages when *K* = 2, although there were gene flows between the 2 groups, especially the North Chinese pig, that clearly mixed with European pig lineages, whereas LW (including FLW) pigs showed signatures of admixture with Chinese pigs. ECN pigs represented by Jinhua (JH) pigs and SCN pigs represented by Luchuan pigs appeared as the 2 ancestral lineages of Chinese pigs when *K* = 3 (Fig. 1d). Altogether, these findings not only confirmed the independent domestication of Chinese and European pigs but also revealed that SCN pigs and ECN pigs have marked genetic differentiation and represent 2 ancient lineages of the Chinese domestic pig.

### SCN and ECN pigs were introgressed into Europe between 220 and 310 years ago

**Figure 2:**
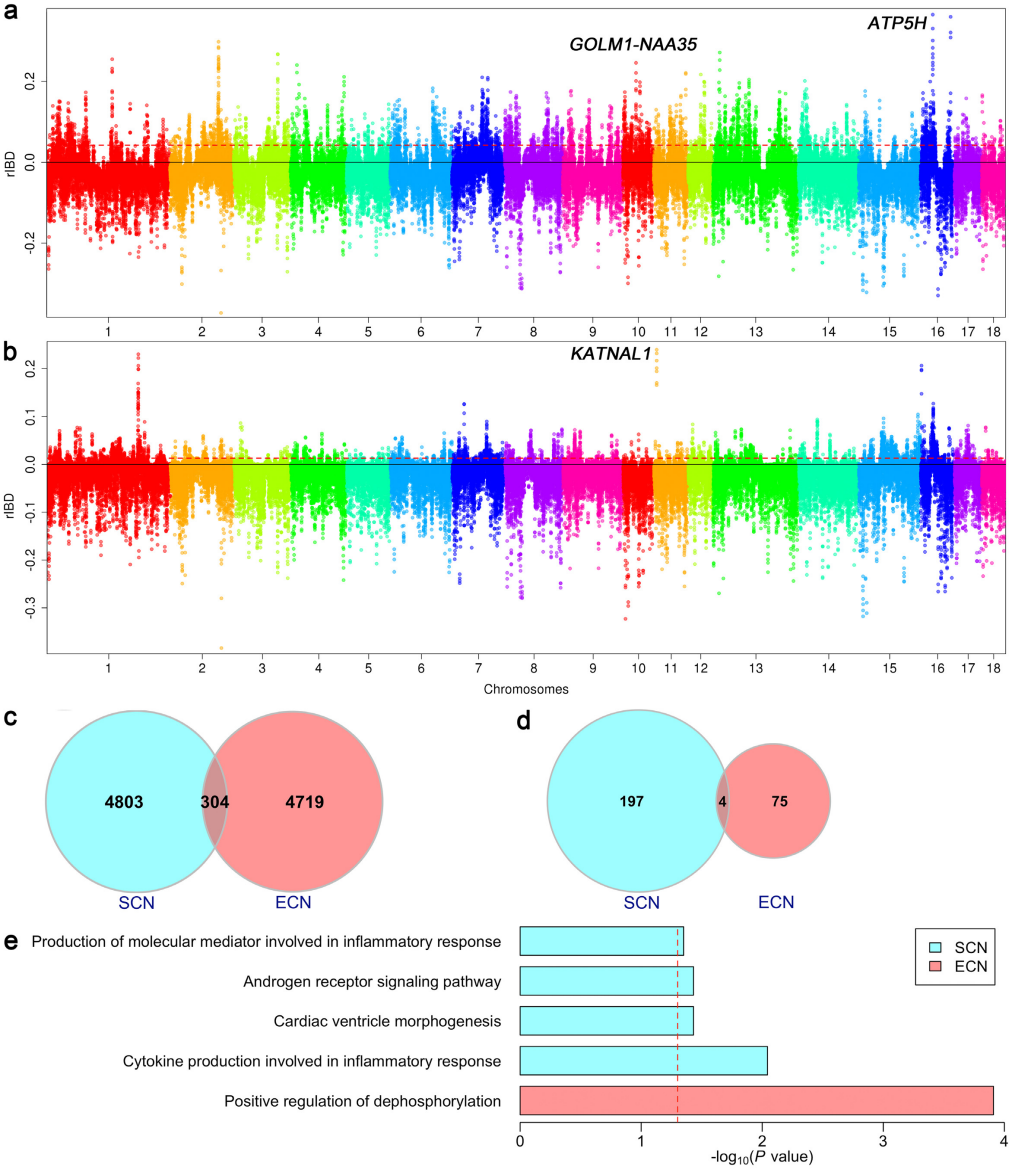
Introgressed Chinese haplotypes in French Large White (FLW) pigs. (**a**) Manhattan plot of rIBD values between FLW and South Chinese (SCN) pigs (positive value) or European wild boars (EWB) (negative value). The red dashed line indicates the top 5% significance threshold. (**b**) Manhattan plot of rIBD values between FLW and East Chinese (ECN) pigs (positive value) or EWB (negative value). (**c**) Venn diagram of introgressed DNA (50-kb windows) from SCN and ECN pigs in FLW pigs. (**d**) Venn diagram of genes in the introgressed regions from SCN and ECN pigs in FLW pigs. (**e**) Significantly enriched GO processes and KEGG pathways of introgressed genes in the introgressed regions from SCN and ECN pigs under selection in FLW pigs.

To determine whether SCN and ECN pigs were introduced into Europe via human-mediated transportation, we performed relative identity-by-descent (rIBD) analysis using whole-genome sequencing data (see Methods). We detected 5,107 and 5,024 50-kb regions with signatures of potential introgression from SCN (Supplementary Table S2) or ECN (Supplementary Table S3) pigs into FLW pigs, respectively (Fig. 2a and b, Supplementary Fig. S4). The introgressed DNA from SCN and ECN pigs differed greatly in FLW pigs, with an overlap of only 6.0% introgression regions (Fig. 2c) and 2.9% genes within these regions (Fig. 2d). We thus performed Gene Ontology (GO) and KEGG pathway enrichment analysis on the genes located in the introgressed regions. The genes within the regions of inferred introgression with SCN pigs and ECN pigs were enriched in the immune-related signaling and fertility pathways, respectively (Fig. 2e). We further used ALDER software [13] to estimate the time of admixture between FLW and SCN or ECN pigs, which yielded an estimate of 53 $\pm $ 9 (265 $\pm $ 45 years) and 54 $\pm $ 9 (270 $\pm $ 45 years) generations ago, respectively. This estimate was consistent with historical records stating that SCN pigs were deliberately transported to England at the onset of the first Industrial Revolution and contributed to the breeding of LW pigs [11]. In addition, these results supported our speculation that ECN pigs were also introduced into Europe to improve the productivity of local pigs between 1685 and 1757.

### The introgressed *GOLM1-NAA35* haplotype from SCN pigs has been under selection to enhance the disease resistance of FLW pigs

We detected 7 genomic regions with strong signatures of introgression from SCN pigs in the genomes of FLW pigs (rIBD value > 0.2; Supplementary Table S4). Two adjacent genes (3,511 bp apart), *GOLM1* and *NAA35*, were located in 1 of these 7 regions. The *GOLM1* gene encodes a type II Golgi transmembrane protein, which is mainly synthesized in the rough endoplasmic reticulum, assists in processing proteins in the Golgi, and is responsive to viral infections [14]. In 2016, Li et al. [15] reported that the *GOLM1*-*NAA35* locus markedly modulated the cytokine interleukin 6 (IL-6) production by human immune cells in response to multiple pathogens. Given the important role of the *GOLM1*-*NAA35* locus in disease resistance, we chose this locus for further study.

**Figure 3:**
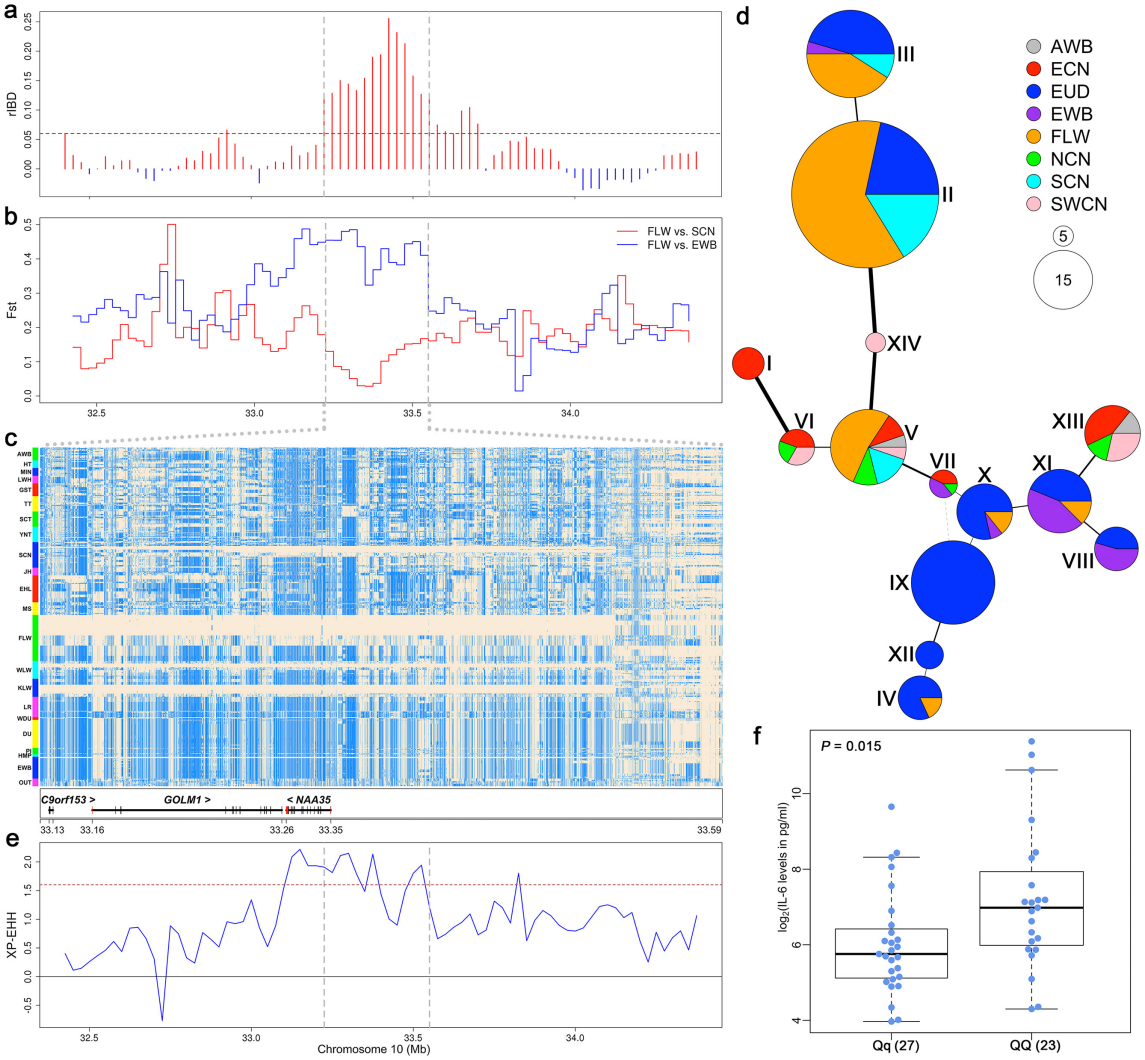
Introgression at the *GOLM1-NAA35* locus. (**a**) rIBD values in a 2-Mb region harboring the *GOLM1*-*NAA35*gene. The brown dashed line indicates the 5% threshold line, and the *GOLM1-NAA35* region is indicated by grey dashed lines. (**b**) Genetic differentiation index (F_ST_) between French Large White (FLW) and European wild boar (EWB) or South Chinese (SCN) pigs. (**c**) Haplotype heat map in the *GOLM1-NAA35* region. Major and minor alleles in FLW pigs are indicated by beige and light blue, respectively. (**d**) Haplotype network in the *GOLM1-NAA35* region. Each circle represents a haplotype, and the size of the circle is proportional to the haplotype frequency. The line width and length represent the difference between haplotypes. Different colors represent pigs from different geographical regions. AWB: Asian (Chinese) wild boars; ECN: East Chinese pig; EUD: European domestic pig; NCN: North Chinese pig; SWCN: Southwest Chinese pig. (**e**) Selection signals in the *GOLM1-NAA35* region by XP-EHH analysis between FLW and other Large White pigs. The brown dashed line indicates the 5% threshold line. (**f**) Serum interleukin 6 (IL-6) contents of FLW pigs homozygous (*QQ*) or heterozygous (*Qq*) for the introgressed *GOLM1-NAA35* haplotypes. The Student *t*-test was used to compute the *P-*value (*P* = 0.015).

We first made a close examination of the rIBD results for a 2-Mb region encompassing the *GOLM1-NAA35* locus (SSC10: 33.20–33.58 Mb on Sscrofa10.2 and 29.15–29.50 Mb on Sscrofa11.1). We found that the frequency of shared IBD haplotypes between FLW and SCN pigs at the *GOLM1*-*NAA35* locus was significantly higher than those in the surrounding regions (Fig. 3a). Moreover, we observed remarkably elevated genetic differentiation (F_ST_) between FLW pigs and European wild boars (EWBs), in contrast to the particularly decreased F_ST_ between FLW and SCN pigs in the *GOLM1*-*NAA35* region we observed (Fig. 3b). In addition, there were 4 main *GOLM1-NAA35* haplotypes in FLW pigs. Most individuals (32 of 36) carried haplotypes similar to those of SCN pig (Fig. 3c).

Next, we used 3,447 SNPs in the *GOLM1*-*NAA35* region to construct an NJ tree (Supplementary Fig. S5). We found that most FLW pigs (n = 32) clustered with SCN pigs to form a branch that was separated from ECN pigs and European pigs, whereas only a small number of FLW pigs (n = 4) clustered with European pigs, which was in stark contrast to a genome-wide NJ tree (Fig. 1a). We further constructed a haplotype network using 298 SNPs at the *GOLM1*-*NAA35* locus (Fig. 3d). We clearly identified haplotype Ⅱ as being the main haplotype in FLW pigs, and this haplotype appeared 37 times in all populations, including 23 times in FLW pigs, 8 times in LW pigs, and 6 times in SCN pigs. The SCN-major haplotype III and haplotype II differed at only 4 different sites, whereas the unique haplotypes (VIII, X and XI) of EWBs and haplotype II differed at >190 sites (Supplementary Fig. S6). These results corroborate the historical introgression of SCN pigs into FLW pigs and illuminate that haplotype II at the *GOLM1*-*NAA35* locus in FLW pigs originated from SCN pigs.

We noted that the introgressed haplotype II was present in other LW pigs at low frequencies but was absent in other European domestic pigs. This was conceivable because all LW populations originated in England where SCN pigs were introduced during the first Industrial Revolution (early 19th century) [7]. Moreover, haplotype III appeared 1 time in EWBs. Considering the outdoor grazing of early European pigs, we believe that EWBs had admixture with European domestic pigs, after which this haplotype was introgressed from European domestic pigs into EWBs.

The haplotype heat map of the *GOLM1*-*NAA35* region shows that the SCN*-*originated haplotype II was frequently present in FLW pigs (Fig. 3c), which suggested that this haplotype may be selected for in FLW pigs. To verify this hypothesis, we first compared the linkage disequilibrium (LD) values (*r^2^*) of the *GOLM1*-*NAA35* region and an upstream (3 Mb) region with the same size as the *GOLM1*-*NAA35* locus. We found that the LD level in the *GOLM1*-*NAA35* region of the FLW population ($r_{0.3}^2\ $= 192.3 kb) was significantly higher than that of all other populations (Supplementary Fig. S7a), whereas the LD value ($r_{0.3}^2$) in the upstream region was only 17.3 kb, which was similar to most populations (Supplementary Fig. S7b). Subsequently, we performed LD analysis for 10,000 81.9-kb regions randomly sampled across the genomes of 36 FLW pigs (Supplementary Fig. S7c). We found that the LD value (*r^2^*) in the *GOLM1*-*NAA35* region ranked in the top 2.6% of the 10,000 bootstrap results, which was a significant outlier (*P* = 0.02) and suggested that the introgressed *GOLM1*-*NAA35* haplotype likely underwent a preference selection in FLW pigs, resulting in a local increase of LD level in this target region. XP-EHH(Cross-population Extended Haplotype Homozygosity) analysis also showed evidence of selection at the *GOLM1*-*NAA35* region in FLW pigs but not in other LW pigs (Fig. 3e).

To examine whether the *GOLM1*-*NAA35* haplotypes were associated with serum IL-6 content in FLW pigs, we collected venous blood from 54 healthy adult FLW sows at the same physiological stage and determined the IL-6 levels in the serum of each individual using an enzyme-linked immunoassay (ELISA) (Supplementary Table S5). Meanwhile, we defined the *GOLM1*-*NAA35* haplotypes for each individual using 2 tag SNPs and then tested the association between these haplotypes and IL-6 content. We found that individuals homozygously carrying the introgressed haplotype (*QQ*) had significantly higher IL-6 concentrations than heterozygotes individuals (*Qq*) (*P* = 0.015; Fig. 3f). Altogether, a sensible explanation for the introgression at the *GOLM1*-*NAA35* locus is that the *GOLM1*-*NAA35* haplotype was historically introgressed from SCN pigs into LW pigs and then has been under preferential selection to improve the effective production of IL-6 in response to pathogens and consequently enhance the resistance to infectious disease of FLW pigs.

Historically, South China was renowned as a land of plague with a humid and stuffy environment. It was notorious for local infectious diseases, including malignant malaria that caused high transmission and mortality rates before the Southern Song Dynasty (1127–1279 AD). This hostile environment imposed severe physiological challenges on the inhabitants of South China [7]. Native inhabitants such as humans and pigs are believed to have evolved the adaptive mechanisms to address this harsh environment, likely via selection of immune-related genes during the long history of colonization of this area. It is thus conceivable that those genes, including *GOLM1-NAA35* within the introgression regions from SCN pigs, are enriched in immune-related signaling pathway genes. Interestingly, a recent genomic analysis unraveled a list of genes related to immune response under selection in southern Han Chinese, including *G6DP* associated with resistance to malaria [16].

### The introgressed *KATNAL1* haplotype from ECN pigs has been preferentially selected to increase the fertility of FLW boars

In FLW pigs, a 200-kb region on chromosome 11 (6.68–6.88 Mb on Sscrofa10.2 and 6.92–7.12 Mb on Sscrofa11.1) showed the strongest (the highest rIBD value) signal of admixture with ECN pigs, and it contained only 1 gene, *KATNAL1. KATNAL1* regulates microtubule dynamics in testicular support cells, affecting the separation and binding of microtubules. Promoting the rapid reorganization of testicular support cell microtubule arrays is an essential process for spermatogenesis and male fertility [17]. Thus, *KATNAL1* plays an important role in spermatogenesis. Given the top introgression signal at the *KATNAL1* locus and the role of *KATNAL1* in boar fertility, we conducted an in-depth analysis focusing on the *KATNAL1* region using the same method as used for the *GOLM1*-*NAA35* locus.

**Figure 4:**
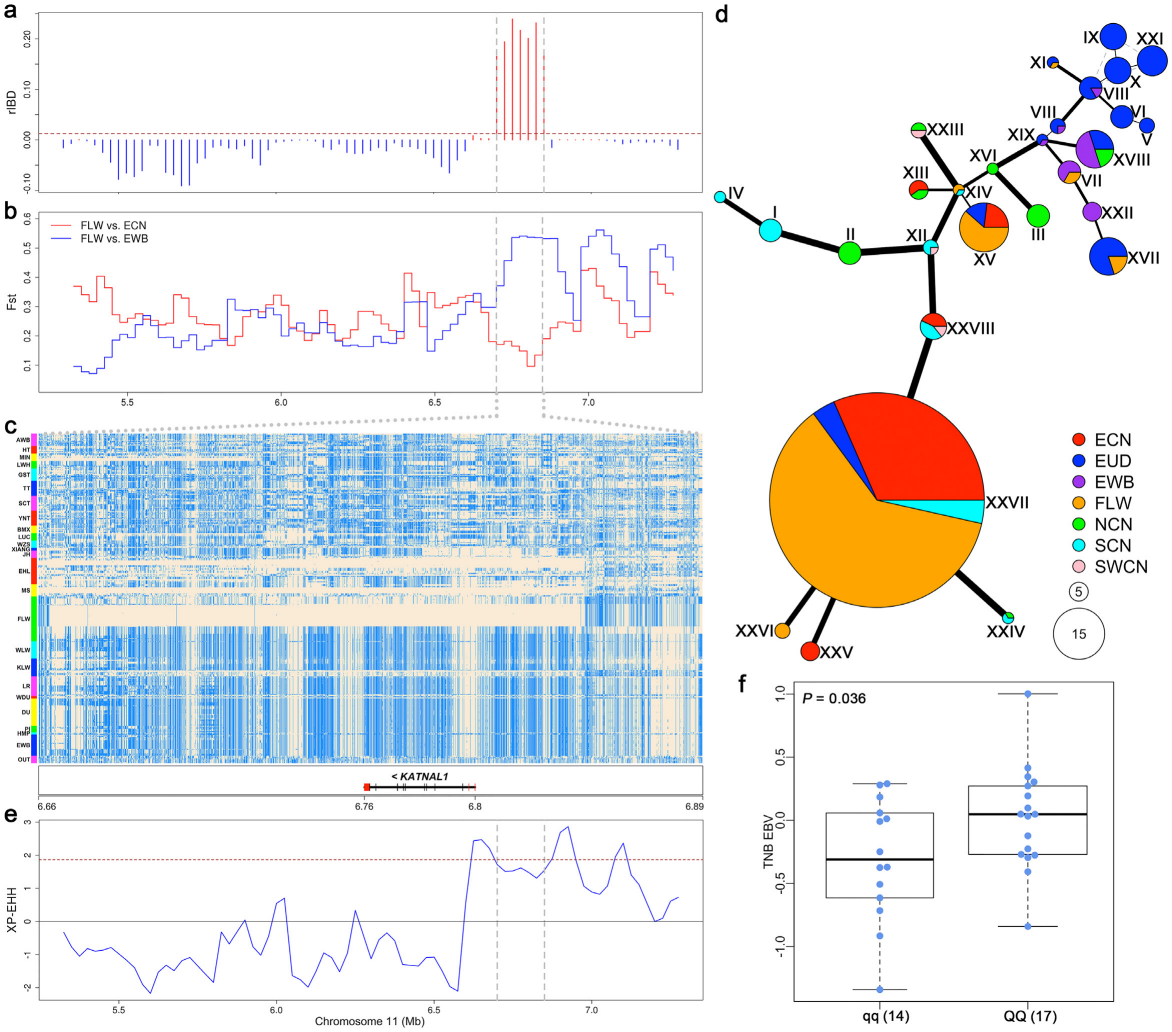
Introgression at the *KATNAL1* locus. (**a**) rIBD values in a 2-Mb region encompassing the *KATNAL1* gene. The brown dashed line indicates the 5% threshold line, and the *KATNAL1* region is indicated by grey dashed lines. (**b**) Genetic differentiation index (F_ST_) between French Large White (FLW) and European wild boar (EWB) or East Chinese (ECN) pigs. (**c**) Haplotype heat map of the *KATNAL1* region. Major and minor alleles in FLW pigs are indicated by beige and light blue, respectively. (**d**) Haplotype network in the *KATNAL1* region. Each circle represents a haplotype, and the size of the circle is proportional to the haplotype frequency. The line width and length represent the difference between haplotypes. Different colors represent pigs from different geographical regions. EUD: European domestic pig; FLW: French Large White; EWB: European wild boar; NCN: North Chinese pig; SCN: South Chinese pig; SWCN: Southwest Chinese pig. (**e**) Selection signals by XP-EHH analysis between FLW and other Large White pigs. The brown dashed line indicates the 5% threshold line. (**f**) Estimated breeding values for total number of piglets born (EBV_TNB) of FLW sows that mated with FLW boars homozygous (*QQ*) or heterozygous (*Qq*) for the introgressed haplotypes. The Student *t*-test was used to compute the *P*-value (*P* = 0.036). The interquartile range and median are indicated by box and bold horizontal line. The top horizontal bar represents the largest value within 1.5 times interquartile range above 75th percentile, and the botton horizontal bar represents the smallest value within 1.5 times interquartile range below 25th percentile. The blue points indicate the EBV_TNB of the samples.

We found that the frequency of the shared IBD haplotype between FLW and ECN pigs in the *KATNAL1* region was particularly higher than that in the surrounding segments (Fig. 4a). There was a remarkable local increase of F_ST_ between FLW pigs and EWBs and a significant decrease of F_ST_ between FLW pigs and ECN pigs in the *KATNAL1* region (Fig. 4b). FLW pigs had 4 main haplotypes in this region. Most individuals (30 of 36) carried haplotypes highly similar to the ECN haplotypes, and the others were similar to EWBs and European domestic pigs (Fig. 4c). Additionally, 30 FLW pigs and ECN pigs were clustered into 1 large clade while only 6 FLW pigs were grouped with European pigs in an NJ tree that was constructed with 529 SNPs in the *KATNAL1* gene (Supplementary Fig. S8). Meanwhile, we constructed a haplotype network using these 529 SNPs (Fig. 4d) and analyzed the nucleotide differences among different haplotypes (Supplementary Fig. S9). The most frequent haplotype (XXVII) appeared 57 times in the 266 tested individuals, including 35 FLW pigs, 18 ECN pigs, 2 ALW pigs, and 2 SCN pigs. This haplotype and its closest ECN haplotype (XXV, at 5 different sites; Supplementary Fig. S9) were divergent from the European pig haplotype groups (Fig. 4d). These results further demonstrated that the *KATNAL1* haplotypes were introgressed from ECN pigs into FLW pigs.

We performed LD bootstrap sampling and XP-EHH analysis to detect evidence of selection at the *KATNAL1* locus in FLW pigs. First, we compared the LD value (*r^2^*) of the *KATNAL1* region and those of 10,000 randomly selected genomic regions with the same size as the *KATNAL1* gene (43.4 kb). We found that the LD level in the *KATNAL1* region ( $r_{0.3}^2$= 437.5 kb) was a significant (*P* = 0.02) outlier, ranking in the top 2.5% of 10,000 bootstrap results (Supplementary Fig. S10). We also detected a significant selection signal at the *KATNAL1* locus in FLW pigs but not in other LW pigs using XP-EHH (Fig. 4e). These results suggest that the introgressed *KATNAL1* haplotype from ECN pigs was preferentially selected for in FLW pigs.

Given the important role of *KATNAL1* in male fertility, the fecundity of ECN pigs, and historical selection for fecundity in FLW pigs, we speculated that the introgressed *KATNAL1* haplotype could contribute to the improvement of male reproductive performance and thus underwent selection in FLW pigs since introgression. To test this hypothesis, we analyzed the association between the *KATNAL1* haplotypes and FLW boar fertility that was represented by the average estimated breeding value (EBV) for TNB of mating sows. We detected a significant difference in boar fertility between 17 homozygous carriers of the introgressed haplotype (*QQ*) and 14 carriers of non-ECN pig haplotypes (*qq*) (*P =* 0.036; Fig. 4f). The EBV for TNB (EBV_TNB) of *QQ* individuals was 0.018, with a difference of 0.32 (which equates to an increase of 0.32 piglets born per parity) compared with *qq* individuals. Because TNB is a complex multi-locus trait, an increase of 0.32 piglets born is substantial for current pig breeding programs. This indicated that the introgressed *KATNAL1* haplotype has been favored and intensively selected by breeders, contributing to the formation of excellent reproductive traits in FLW pigs.

### *AHR* haplotypes that associate with increased litter size were likely introgressed from ECN pigs into LW pigs

**Figure 5:**
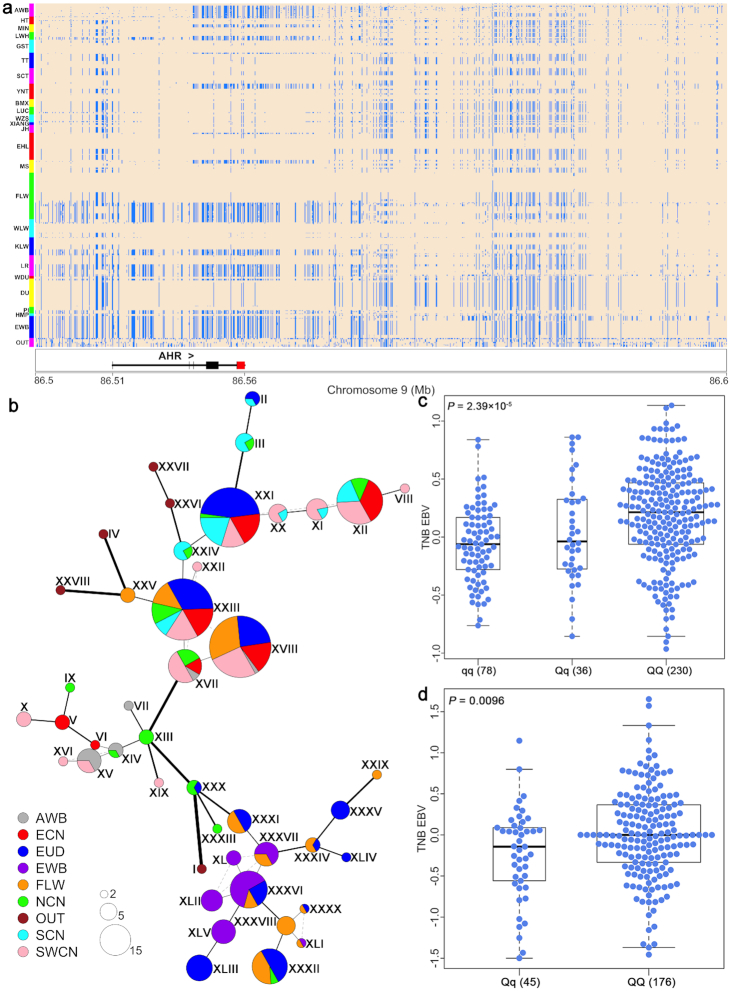
Human-mediated introgression at the *AHR* locus. (**a**) Haplotype heat map of a 100-kb region encompassing the *AHR* gene on chromosome 9 (86.5–86.6 Mb on Sscrofa11.1 and 95.4–95.56 Mb on Sscrofa10.2). Major and minor alleles in French Large White (FLW) pigs are indicated by beige and light blue, respectively. (**b**) *AHR* haplotype network. Each pie chart represents 1 unique haplotype, and the radius of the pie chart is proportional to 2 × log_2_ (number of chromosomes with that haplotype). The width and length of the edges are proportional to log_2_ (number of pairwise differences between the joined haplotypes) + 1, and the thinnest edge represents a difference of 1 mutation. (**c**) FLW sows carrying the homozygous archaic *AHR* haplotype show significantly (*P* = 2.39 × 10^−5^) lower estimated breeding values for total number born EBV (EBV_TNB), compared with those who do not carry the archaic haplotype. (**d**) Erhualian sows homozygously carrying the archaic haplotype (*QQ*) have higher (*P* = 0.0096) EBV_TNB than heterozygous carriers (*qq*). The interquartile range and median are indicated by box and bold horizontal line. The top horizontal bar represents the largest value within 1.5 times interquartile range above 75th percentile, and the botton horizontal bar represents the smallest value within 1.5 times interquartile range below 25th percentile. The blue points indicate the EBV_TNB of FLW (**c**) and EHL (**d**).AWB: Asian wild boar; BMX: Bamaxiang pig; DU: Duroc; EHL: Erhualian pig; EWB: European wild boar; GST: Tibetan pig (gansu); HMP: Hampshire; HT: Hetao pig; JH: Jinhua pig; KLW: Korea Large White pig; LR: Landrace; LUC: Luchuan pig; LWH: Laiwu pig; MIN: Min pig; MS: Meishan pig; OUT: outgroup; PI: Pietrain; SCT: Tibetan pig (Sichuan); TT: Tibetan pig (Tibet); WDU: White Duroc; WLW: Dutch Large White pig; WZS: Wuzhishan pig; XIANG: Xiang pig; YNT: Tibetan pig (Yunnan).

In 2014, Bosse et al. [11] found that Chinese haplotypes in a 6.8-Mb region on chromosome 9 containing the *AHR* gene were introgressed into European pigs and were preferentially selected to increase fertility during the development of LW pigs. We also conducted a shared haplotype test (rIBD) between 121 Chinese pigs and 64 LW pigs in this 6.8-Mb region. We confirmed the presence of Chinese-derived haplotypes in European pigs including FLW pigs, with a strong introgression signal at the *AHR* locus (SSC9: 92.25–97.45 Mb in Sscrofa10.2 and 83.90–88.40 Mb in Sscrofa11.1) (Supplementary Fig. S11). To explore the geographic origin of the introgressed Chinese *AHR* haplotypes, we first constructed a phylogenetic tree of all sequenced individuals around the *AHR* region, and surprisingly found that most domestic pigs were clustered together with small genetic distance but were divergent from European and Asian wild boars (Supplementary Fig. S12a). We further reconstructed and visualized haplotypes around the *AHR* gene (86.5–86.6 Mb on Sscrofa11.1 and 95.4–95.56 Mb on Sscrofa10.2) and found that most haplotypes of LW pigs were highly similar to those of Chinese EHL pigs and Tibetan pigs (Fig. 5a). In an NJ tree of this region, 15 FLW pigs gathered with EHL pigs and Tibetan pigs, defining a branch distinct from other Chinese breeds (Supplementary Fig. S12b). Moreover, the most frequent haplotype (XVIII) appeared 99 times in all 266 sequenced individuals, including 30 FLW pigs, 24 other LW pigs, 17 EHL pigs, 26 Tibetan pigs, and 2 Asian wild boars (Fig. 5b). The nucleotide difference between this haplotype (XVIII) and Chinese haplotype XVII was only 6, in contrast to 70 between this haplotype and EWB haplotype XLII (Supplementary Fig. S13). In addition, FLW pigs and EHL pigs had the smallest F_ST_ values with the exception of Tibetan pigs and other LW pigs (Supplementary Fig. S12c). Given the geographic distance between Tibet and Europe and the lack of any historical records describing the importation of Tibetan pigs into Europe, we argue that Chinese-derived *AHR* haplotypes in FLW pigs were most likely introgressed from ECN pigs such as EHL pigs through human-mediated transportation ∼200–300 years ago.

We noted that the introgressed haplotype XVIII was desirable for increasing the EBV_TNB of both FLW pigs (Fig.   5c) and EHL pigs (Fig. 5d). By genotyping the haplotype tag SNPs and 1-way analysis of variance (see Methods), we found that homozygous carriers of the introgressed *AHR* haplotype (XVIII) had 0.24 higher EBV_TNB than heterozygous carriers ( *P* = 0.001, Supplementary Table S6) in EHL pigs. Moreover, the introgressed *AHR* haplotype was significantly associated with increased EBV_TNB in FLW sows, with an additive effect value of 0.25 ( *P* = 2.39 e^−05^; Fig. 5c, Supplementary Table S7), which was in agreement with the report of Bosse et al. [11]. Similar to the *KATNAL1* and *GOLM1*-*NAA35* regions, the LD value of FLW pigs at the *AHR* gene region ranked in the top 7% (significant outlier) of all 10,000 bootstrap values (*P* = 0.03; Supplementary Fig. S14). We also detected a significant iHS selection signal within the FLW pig population (Supplementary Fig. S15). These findings enabled us to conclude that the introgressed *AHR* haplotype had been under a preferential selection to improve the fertility of FLW pigs.

## Discussion

### Introgression of both SCN and ECN pig DNA contributed to the genetic improvement of European modern pig breeds

European and Asian domestic pigs were independently domesticated from European and Asian wild boars, respectively, nearly 10,000 years ago [3, 5, 6]. In this study, population genetics analyses confirmed striking genetic differences between Chinese and European domestic pigs and uncovered obvious genetic differentiation between SCN and ECN pigs, which represent 2 ancestral lineages of Chinese pigs. Of note, we identified Chinese haplotypes in FLW pigs, which were introgressed from both SCN and ECN pigs. We inferred that the introgression events occurred 220–310 years ago, which was in accordance with historical records that SCN pigs were transported to England through the Guangzhou port during the first Industrial Revolution [7]. Our results also supported the speculation that ECN pigs were introduced into Europe, likely through the Shanghai and Ningbo ports, in the decades before the Qing Dynasty imposed a ban on the sea in 1757. Thus, we believe that both SCN and ECN pigs were introduced to Europe to improve the production performance of local breeds, contributing to the development of modern European commercial pig breeds. Taking the *GOLM1-NAA35* and *KATNAL1* loci as examples, the introgressed *GOLM1-NAA35* haplotype from SCN pigs was beneficial for improving disease resistance in FLW pigs, and the introgressed *KATNAL1* haplotype from ECN pigs was favorable for boar fertility and provided genetic variations for the development of high-fecundity FLW pigs. In addition, we show that the *AHR* haplotype associated with increased sow litter size was introduced from ECN pigs into European pigs, such as the Large White breed, through human-mediated transportation and hybridization some 200–300 years ago. It has further experienced preferential selection, presumably during the past decades, and is present at high frequency in FLW pigs, contributing to the improvement of the reproductive performance of this breed. It shows that human-driven crossbreeding plays important roles in the development of global pig breeds, illustrating a complex breeding history of domestic pigs. These findings not only advance our understanding of the breeding history of modern European commercial pig breeds but also provide insights into the genetic mechanisms underlying economically important traits in pigs.

## Methods

### Samples

All procedures used for this study and involving animals were in compliance with guidelines for the care and use of experimental animals established by the Ministry of Agriculture of China. The ethics committee of Jiangxi Agricultural University approved this study. This study used genome-wide resequencing data from 266 animals (Supplementary Table S1), of which 153 pigs were resequenced for this study and 113 genome sequence data sets were downloaded from public databases (Registration Nos. PRJEB1683 [18], PRJEB9922 [19], PRJNA260763 [20], PRJNA398176 [21], PRJNA213179 [22], and PRJNA488327 [23]). Among the 153 pigs, 36 were FLW sows and were collected from the Guangdong WENS Food Company (24 individuals) and Jiangxi Lvhuan Animal Husbandry Company (12 individuals). The 36 FLW sows were selected according to the following criteria. First, we calculated the relationship coefficients of all individuals in the nucleus populations of the 2 companies using DMU software [24] and pedigree records. Then we selected sows with a small relationship coefficient and excellent litter sizes (TNB >16). Finally, we chose 36 prolific individuals with distant kinship according to a phylogenetic relationship network constructed by Cytoscape v3.2.1 (Cytoscape, RRID:SCR_003032) [25] (Supplementary Fig. S1). In total, there were 27 wild boars from China and Europe, 7 outgroup individuals, 121 pigs from Chinese indigenous breeds, and 111 pigs from European commercial breeds. According to the geographic distribution, Chinese domestic pigs were divided into ECN (37) pigs, SCN (20) pigs, SWCN (36) pigs, and NCN (28) pigs (see Supplementary Table S1 for details). In addition, whole-genome sequence data of 28 LW pigs was downloaded from public databases, with 14 individuals submitted by Seoul National University [20] and another 14 individuals submitted by Wageningen University [18]. To identify the source of these 28 LW pigs, we downloaded the Illumina 60 K chip SNP data set of 76 LW pigs [26], including 20 NLW, 16 Danish Large White pigs (DLW), 20 Chinese Large White pigs (CLW), and 20 American Large White pigs (ALW). Next, we retrieved the same 60 K chip SNPs from the whole-genome sequence data sets of the 28 LW pigs. We filtered out SNPs with a major allele frequency (MAF) <0.05, a call rate <90%, and an LD (*r^2^*) value >0.3 using PLINK v1.9 (PLINK, RRID:SCR_001757) [27], and we performed PCA and NJ tree analyses using the remaining SNPs to identify the origin of the 28 LW pigs (Supplementary Fig. S2).

### Whole-genome sequencing and SNP calling

We extracted genomic DNA from the ear tissues of 153 pigs using a routine phenol/chloroform protocol, and eligible samples were delivered to the Novogene company (Beijing, China). Sequencing was performed on Hiseq 2000 or 2500 instruments (Illumina HiSeq 2500 System, RRID:SCR_016383). The sequencing libraries were constructed with 125-bp paired ends (PE125), a 500-bp average insert fragment size, and a fragment size <800 bp. The genome sequencing coverage of each individual was ≥20×, with a minimum data of 60 G.

#### Quality control

We obtained the raw sequencing data from the Hiseq sequencing platform using raw image data. We obtained clean data for performing downstream analysis after performing the following steps: (i) removal of the linker sequence, (ii) retention of reads with Q20 of >90% (probability of base recognition correct rate >99%) and Q30 of >85% (probability of base recognition correct rate >99.9%) [28], (iii) culling of short repeat DNA segments, and (ⅳ) filtering reads with 3 consecutive “N”.

#### Mutation detection

We established a reference genome index of Sscrofa 10.2 [6] using the index function in BWA v0.7.12 (BWA, RRID:SCR_010910) [29]. We blasted paired-end reads against the index using an algorithm from BWA and obtained binary bam files from sam files by means of SAMtools v1.4 (SAMTOOLS, RRID:SCR_002105) [30]. We used samblaster v0.1.22 (SAMBLASTER, RRID:SCR_000468) [31] to reject redundancy information and calculated the alignment rate between resequencing data and the reference genome, as well as coverage and sequencing depth. We sorted binary bam files via GATK v3.7 (GATK, RRID:SCR_001876) [32]. We used the HaplotypeCaller function for mutation detection across each chromosome of each individual and obtained an SNP data set of the 266 individuals by deleting insertion and deletion information. We filtered out SNPs with MAF <0.01 and call rate <90% using PLINK v1.9 [27]. We used the remaining 32.7 million SNPs in the data set for subsequent statistical analysis.

### Population genetic analysis

First, we generated an SNP data set with MAF >0.05 and call rate >90% from autosomal SNPs from 259 pigs (*S. scrofa*) excluding 7 OUT individuals. Second, we pruned SNPs with an LD (*r^2^*) decay of >0.3 in each window with 50 SNPs using the command indep-pairwise (50 10 0.3) in PLINK v1.9 [27]. Then 4 principal components of each individual were estimated using the –pca command in GCTA software [33]. The average shared allele (1-Dst) distance matrix between individuals was constructed using the command –distance-matrix in PLINK v1.9. A rootless NJ tree was constructed via phylip v3.69 (PHYLIP, RRID:SCR_006244) [34] and was visualized with FigTree v1.42 (FigTree, RRID:SCR_008515). We also explored the unbiased estimation method proposed by Weir and Cockerham [35] to calculate the genetic differentiation (F_ST_) matrix between 14 Chinese pig breeds and 6 European pig breeds using the –fst command in PLINK v1.9 [27]. Then, we constructed an interbreed NJ tree using phylip v3.69 [34]. ADMIXTURE (ADMIXTURE, RRID:SCR_001263) [36] was used to estimate the ancestral lineage composition under default parameters. First, we removed the OUT group and populations with <5 individuals. Then we randomly selected 6 individuals from the remaining 21 populations and filtered out SNPs with an MAF of <0.05, an LD (*r^2^*) of >0.3, and call rates <90%. Finally, we used a data set with 125 individuals and 658,601 SNPs to analyze the ancestral lineage composition patterns. In addition, we used TreeMix v1.12 [37] to infer the genetic differentiation among populations. We set OUT as the outgroup population, excluding populations with <6 samples and SNPs with MAF <0.05 and call rate <90%. We used the data set with 19,282,590 SNPs to estimate genetic differentiation among 21 populations under no migration events via TreeMix v1.12 [37].

### Introgression analysis

We detected the introgression signals between Chinese pigs (ECN and SCN pigs) and FLW pigs using an IBD sharing approach [11]. First, we used a data set with 266 individuals and ∼20 million SNPs to phase haplotypes using the fastPhase function [38] in Beagle v4.0 and to detect IBD fragments in each individual using the fastIBD function [39]. Then we divided the whole genome into numbers of 50-kb windows (25-kb sliding) and calculated the shared IBD haplotype numbers between 2 populations (FLW vs EWB, FLW vs ECN, and FLW vs SCN) in each window. We phased the haplotypes and detected the IBD regions independently 10 times and then normalized the IBD values (nIBD). The nIBD values ranged from 0 (no shared IBD detected) to 1 (all individuals shared the IBD haplotype). Finally, we used the rIBD (relative frequency of IBD) statistic to measure the shared IBD between FLW pigs and SCN or ECN pigs, respectively (rIBD_FLW/SCN_ = nIBD_FLW/SCN_ − nIBD_FLW/EWB_, rIBD_FLW/ECN_ = nIBD_FLW/ECN_ − nIBD_FLW/EWB_), where a positive rIBD indicated potential introgression and 5% empirical distribution in the far right tail was set as the significance threshold. For genomic regions showing strong rIBD introgression signals in FLW pigs, we further estimated F_ST_ between FLW pigs and EWBs, as well as FLW pigs and Chinese pigs (SCN pigs or ECN pigs), respectively. We also constructed haplotype networks using SNPs with MAF >0.05 and call rates >90% at the *GOLM1-NAA3* (298 SNPs) and *KATNAL1* (529 SNPs) loci, and using all SNPs (217 SNPs) that were observed at least twice in the 266 resequenced individuals at the *AHR* locus. We explored the fastPhase function with 1,000 iterations in Beagle v4.0 (BEAGLE, RRID:SCR_001789) [39] to phase haplotypes and used the “haploNet” command in the R package “pegas” [40] to calculate the pairwise differences between haplotypes. We selected SNPs with MAF >0.05, call rate >90%, and LD (*r^2^*) <0.3 using PLINK v1.9 [27] and then explored the selected SNPs to estimate the admixture time between populations by means of ALDER v1.0.3 under default parameters [13]. In short, we used the “convert” function in EIGENSTRAT [41] to convert the data format. We set FLW as a mixed population, EWB and SCN as 1 reference population, EWB and ECN as another reference population, and 5 years as 1 generation.

### Signature of selection

We used the data set that excluded SNPs with an MAF of <0.05 and a call rate <90% in the whole-genome SNP data set of 36 FLW pigs to calculate the correlation coefficient (*r^2^*) of each SNP pair in a target region using the commands --r2 inter-chr --ld-window-r2 0 in PLINK v1.9 [40], and we used the average *r^2^* as the LD value in the region. Meanwhile, we randomly selected 10,000 regions with the same size as the target region across the genome, and we calculated the average *r^2^* of each region in the 36 FLW pigs. Finally, we visualized the density curve of the 10,000 bootstrap values using R. Furthermore, we used commands --ihs [42] and --xpehh [43] under default parameters in selscan [44] software to detect the signatures of selection in 50-kb windows with a step size of 25 kb in FLW pigs.

### Haplotype association analysis

#### The *GOLM1-NAA35* locus

We detected the serum IL-6 levels in 54 mature FLW sows at an age of 2–2.5 years from the same farm using the Porcine IL-6 ELISA Kit (Shanghai Keshun Biological Technology, China). The concentration of each individual was determined from the averaged repeat of 3 trials per individual. Meanwhile, we selected 2 tag SNPs to distinguish the introgressed haplotypes (II and III) from the other haplotype in the *GOLM1-NAA35* region in FLW pigs (Fig. 3e). The tag SNPs were genotyped by Sanger sequencing PCR products amplified with specific primers (Supplementary Table S5). A Student *t-*test was used to detect the association between haplotypes and the serum IL-6 concentrations (log_2_ (IL-6 values)).

#### The *KATNAL1* locus

We collected 765 FLW sows and 31 FLW boars from the Jiangxi Lvhuan Farming Group. First, we filtered parities with litter size <5 piglets. Then we set estrus, year, season, parity, and pregnancy duration as fixed effects, and mating boars and random sow effects as random effects. We then estimated the EBV for TNB of 765 FLW pigs via DMU software [24] and pedigree information. Next, we genotyped 8 tagged SNPs to distinguish each *KATNAL1* haplotype in the 31 FLW boars by PCR amplification and Sanger sequencing with primers listed in Supplementary Table S8. We denoted the introgressed XXVII haplotype from ECN pigs as *Q* (Fig. 4e) and the other haplotypes as *q* (Supplementary Table S9). Finally, we used a Student *t*-test to test the association between *KATNAL1* haplotypes and the average EBV_TNB of mating sows of the 31 FLW boars.

#### The *AHR* locus

We genotyped 2 tagged SNPs representing the *AHR* haplotypes for 344 FLW sows by PCR amplification and Sanger sequencing with primers listed in Supplementary Table S6. We identified 230 *QQ* sows homozygous for the introgressed haplotype, 36 *Qq* sows, and 78 *qq* sows who were missing the introgressed haplotypes (Supplementary Table S6). Then we tested the association between the *AHR* haplotypes and the EBV_TNB of the 344 sows using single-factor analysis of variance. Furthermore, we collected 221 Erhualian sows with multiparity records from Jiangsu Province and calculated the EBV_TNB of these sows using DMU software and pedigree information as mentioned above. We genotyped a tag SNP in the *AHR* region by Sanger sequencing PCR products with specific primers (Supplementary Table S7). We detected 176 *QQ* sows homozygous for the introgressed haplotype and 45 heterozygous (*Qq*) sows. We used a Student *t*-test to examine the association between *AHR* haplotypes and EBV_TNB in Erhualian sows.

## Availability of Supporting Data and Materials

On top of the public data sets used, previously unpublished raw sequencing is available via NCBI Bioproject PRJNA550237. All other supporting data and materials are available in the *GigaScience* GigaDB database [45].

## Additional Files

**Supplementary Figure 1:** Pedigree-based relationship network among individuals constructed by Cytoscape v3.2.1. (a) Relationship network of French Large White sows from the WENS company in Guangdong Province. Twenty-four resequenced individuals are highlighted by red dots. (b) Relationship network of French Large White sows from the Lvhuan company in Jiangxi Province. Twelve resequenced individuals are highlighted by red dots. Yellow dots indicate unsequenced individuals. Larger dots indicate sows with higher litter size. The longer and thicker line represents more distant relationship.

**Supplementary Figure 2:** Genetic relationships of Large White pigs from different countries. (a) Principal component analysis. (b) Neighbor-joining clustering tree. ALM, American Large White pig; CLW, Chinese Large White pig; DLW, Danish Large White pig; FLW, French Large White pig; KLW, Korea Large White pig; LR, Landrace; NLW/WLW, Dutch Large White pig.

**Supplementary Figure 3:** Genetic relationships among 21 Chinese and Western pig populations inferred using the TreeMix program without migration edges. OUT was used as an outgroup to root the tree. The length of the branch is proportional to the drift of each population. The scale bar shows 10 times the average standard error (s.e.) of the entries in the sample covariance matrix. The colored dashed lines in the phylogenetic tree represent different genetic groups. Cyan, red, pink, green, and blue dashed lines represent SCN, ECN, SWCN, NCN, and EUD, respectively. The abbreviations of these 21 pig population designations are expanded in the legend of Fig. 1.

**Supplementary Figure 4:** Distribution curves of rIBD values. (a) Distribution curve of rIBD values between FLW and SCN (positive value) or EWB (negative value). The red dashed line indicates the top 5% significance threshold; (b) Distribution curve of rIBD values between FLW and ECN (positive value) or EWB (negative value). The red dashed line indicates the top 5% significance threshold.

**Supplementary Figure 5:** Neighbor-joining tree in the *GOLM1-NAA35* region. The red arc indicates the major clade that includes French Large White (FLW) pigs, other Large White pigs, Luchuan (LUC), Wuzhishan (WZS), and Bamaxiang (BMX) pigs from South China. Red: ECN; green: NCN; cyan: SCN; pink: SWCN; blue: EUD; grey: AWB; purple: EWB; brown: OUT; orange: FLW.

**Supplementary Figure 6:** Haplotype difference at the *GOLM1-NAA35* locus.

**Supplementary Figure 7:** Linkage disequilibrium (LD) analysis for *GOLM1-NAA35* haplotypes. (a) LD decay in the *GOLM1-NAA35* region. LD values were estimated using whole-genome sequence data of 6 individuals randomly selected from each population. The y-axis indicates the physical distance, the ordinate indicates the predicted LD(*r^2^*) value, and the horizontal dashed line indicates the threshold line (*r^2^*= 0.3); (b) LD decay in an upstream (3 Mb) genomic region of the same size as the *GOLM1-NAA35* region; (c) density curve of LD (*r^2^*) bootstrap values for 10,000 regions of the same size as the *GOLM1-NAA35* region in French Large White pigs. The red dashed line represents the LD (*r^2^*) value in the *GOLM1-NAA35* region.

**Supplementary Figure 8:** Neighbor-joining phylogenetic tree of the tested individuals in the *KATNAL1* region. The red arc represents the major clade that includes French Large White (FLW) pigs, other Large White pigs, Erhualian (EHL), and Meishan (MS) pigs from East China. Red: ECN; green: NCN; cyan: SCN; pink: SWCN; blue: EUD; grey: AWB; purple: EWB; brown: OUT; orange: FLW.

**Supplementary Figure 9:** Haplotype difference in the *KATNAL1* gene.

**Supplementary Figure 10:** Linkage disequilibrium (LD) at the *KATANL1* locus in French Large White pigs. This figure shows the density curve of LD (*r^2^*) bootstrap values for 10,000 regions with the same size as *KATNAL1* that were randomly selected across the whole genome in French Large White pigs. The red dashed line represents the LD (*r^2^*) value of the *KATNAL1* gene.

**Supplementary Figure 11:** rIBD and selection signals around the *AHR* region in Large White pigs. (a) rIBD between Large White pigs and Chinese domesticated pigs in the SSC9: 90–100 Mb region (Sscrofa10.2, 81.8–90.6 Mb on Sscrofa11.1). The brown dotted line indicates the 5% threshold line. (b) Selection signals detected by the XP-EHH analysis between Large White pigs and European wild boars. (c) Selection signals within Large White pigs revealed by the iHS analysis. The brown dashed line represents the genome-wide 5% threshold line, and the introgression region is indicated by 2 grey dashed lines. The pink shaded area represents the *AHR* gene region.

**Supplementary Figure 12:** Genetic relationships between French Large White pigs and other pig breeds in the *AHR* region. (a) Neighbor-joining tree in the *AHR* gene. (b) Neighbor-joining tree in the SSC9: 86.5–86.6 Mb (Sscrofa11.1, 95.4–95.6 Mb on Sscrofa10.2) region encompassing the *AHR* gene. The red arc represents the major clade of French Large White (FLW) pigs. (c) Box plot of genetic differentiation between FLW pigs and other pig breeds in the *AHR* region (SSC9: 86.5–86.6 Mb). Different colors represent pig breeds from different geographical regions. Grey represents the genetic differentiation index between FLW pigs and Asian wild boars (AWB). Red, cyan, pink, green, blue, and purple boxes represent the genetic differentiation index (F_ST_) between FLW and ECN, SCN, SWCN, NCN, EUD, and EWB, respectively.

**Supplementary Figure 13:** Haplotype difference at the *AHR* locus.

**Supplementary Figure 14:** Linkage disequilibrium (LD) at the *AHR* locus in French Large White pigs. This figure shows the density curve of LD (*r^2^*) bootstrap values for 10,000 randomly selected regions with the same size as the *AHR* gene in French Large White pigs. The red dashed line represents the LD (*r^2^*) value in the *AHR* gene.

**Supplementary Figure 15:** Selection signals in the *AHR* region in French Large White pigs. The signals were detected by the iHS analysis. The *AHR* gene region is indicated by 2 vertical dashed lines.

**Supplementary Table 1:** Animals and their whole-genome sequencing information

**Supplementary Table 2:** The 50-kb regions of potential introgression from South China pigs into French Large White pigs

**Supplementary Table 3:** The 50-kb regions of potential introgression from East China pigs into French Large White pigs

**Supplementary Table 4:** Strong candidate regions encompassing introgressed Chinese haplotypes in the genomes of French Large White pigs

**Supplementary Table 5:** *GOLM1*-*NAA35* haplotypes and serum interleukin 6 concentrations of 54 French Large White sows

**Supplementary Table 6:** Estimated breeding values for total number of piglets born (EBV_TNB) and *AHR* haplotypes of 224 Erhualian sows

**Supplementary Table 7:** Estimated breeding values for total number of piglets born (EBV_TNB) and *AHR* haplotypes of 344 French Large White sows

**Supplementary Table 8:** Primers for amplification of 8 tag SNPs for identifying *KATNAL1* haplotypes

**Supplementary Table 9:** *KATNAL1* haplotypes and fertility of 31 French Large White boars

giaa014_GIGA-D-19-00160_Original_Submission

giaa014_GIGA-D-19-00160_Revision_1

giaa014_GIGA-D-19-00160_Revision_2

giaa014_Response_to_Reviewer_Comments_Original_Submission

giaa014_Response_to_Reviewer_Comments_Revision_1

giaa014_Reviewer_1_Report_Original_Submission Mirte Bosse -- 6/17/2019 Reviewed

giaa014_Reviewer_1_Report_Revision_1 Mirte Bosse -- 10/22/2019 Reviewed

giaa014_Reviewer_1_Report_Revision_2 Mirte Bosse -- 12/6/2019 Reviewed

giaa014_Reviewer_2_Report_Original_Submission Marina Sanchez -- 8/19/2019 Reviewed

giaa014_Supplemental_Figures_and_Tables

## Abbreviations

ALW: American Large White; bp: base pairs; BWA: Burrows-Wheeler Aligner; DLW: Danish Large White; EBV: estimated breeding value; ECN: Eastern Chinese; EHL: Erhualian; ELISA: enzyme-linked immunoassay; FLW: French Large White; GATK: Genome Analysis Toolkit; GO: Gene Ontology; IL-6: interleukin 6; JH: Jinhua; kb: kilobase pairs; KEGG: Kyoto Encyclopedia of Genes and Genomes; LD: linkage disequilibrium; LUC: Luchuan; LW: Large White; MAF: major allele frequency; Mb: megabase pairs; NCBI: National Center for Biotechnology Information; NJ: neighbor joining; NLW: Dutch Large White; OUT: outgroup; PCA: principal component analysis; rIBD: relative identity-by-descent; SCN: Southern Chinese; SNPs: single-nucleotide polymorphism; TNB: total number born.

## Competing Interests

The authors declare that they have no competing interests.

## Funding

This study is supported by the Natural Science Foundation of China (31525023) and the National Key Research Project of China (2016ZX08006-5).

## Authors' Contributions

J.R. and L.H. designed the study and analyzed the data. J.R., H.C., and L.H. wrote the paper. H.C., M.H., and B.Y. performed the bioinformatic analyses. H.C., M.H., Z.D., Z.W., and Y.H. collected data and performed sequencing and genotyping experiments.
