# Supplementary material for: Introgression of Eastern Chinese and Southern Chinese haplotypes contributes to the improvement of fertility and immunity in European modern pigs
Source: Gigascience. 2020 Mar 6;9(3):giaa014. doi: 10.1093/gigascience/giaa014 (PMC7059266; doi:10.1093/gigascience/giaa014)
Supplement: giaa014_GIGA-D-19-00160_Original_Submission [file giaa014_giga-d-19-00160_original_submission.pdf]

GigaScience

Population genomic data reveal origin and phenotypic effect of Chinese haplotypes introgressed into European modern pigs

--Manuscript Draft--

**Manuscript Number:**	GIGA-D-19-00160
**Full Title:**	Population genomic data reveal origin and phenotypic effect of Chinese haplotypes introgressed into European modern pigs
**Article Type:**	Research
**Funding Information:**	Natural Science Foundation of China (31525023) — Prof. Jun Ren<br>National Key Research Project of China (2016ZX08006-5) — Prof. Jun Ren
**Abstract:**	Pigs were domesticated independently from European and Asian wild boars nearly 10,000 years ago. Chinese indigenous pigs were historically introduced to improve Europe local pigs. However, the geographic origin and biological functions of introgressed Chinese genes in modern European pig breeds remain largely unknown. Here we explored whole-genome sequencing data from 266 Eurasian wild boars and domestic pigs to produce a fine-scale map of introgression between French Large White (FLW) and Chinese pigs. We show that FLW pigs had historical admixture with both South Chinese (SCN) and East Chinese (ECN) pigs 200–300 years ago. A set of SCN haplotypes are beneficial for improving disease resistance and those of ECN haplotypes are favorable for better reproductive performance in FLW pigs. Intriguingly, we found both human mediated and archaic introgression events at the AHR locus, at which the archaic haplotype contribute to increased fertility in both ECN and FLW pigs. This study advances our understanding of the development history of global domestic pigs and highlights the importance of artificial hybridization and natural archaic introgression in the formation of phenotypic characteristics in domestic animals.
**Corresponding Author:**	Jun Ren<br><br>CHINA
**Corresponding Author Secondary Information:**	
**Corresponding Author's Institution:**	
**Corresponding Author's Secondary Institution:**	
**First Author:**	Hao Chen
**First Author Secondary Information:**	
**Order of Authors:**	Hao Chen<br>Min Huang<br>Bin Yang<br>Zhongping Wu<br>Zheng Deng<br>Yong Hou<br>Jun Ren<br>Lusheng Huang
**Order of Authors Secondary Information:**	
**Additional Information:**	
**Question**	**Response**

Are you submitting this manuscript to a special series or article collection?	No
**Experimental design and statistics**<br><br>Full details of the experimental design and statistical methods used should be given in the Methods section, as detailed in our Minimum Standards Reporting Checklist. Information essential to interpreting the data presented should be made available in the figure legends.<br><br>Have you included all the information requested in your manuscript?	Yes
**Resources**<br><br>A description of all resources used, including antibodies, cell lines, animals and software tools, with enough information to allow them to be uniquely identified, should be included in the Methods section. Authors are strongly encouraged to cite Research Resource Identifiers (RRIDs) for antibodies, model organisms and tools, where possible.<br><br>Have you included the information requested as detailed in our Minimum Standards Reporting Checklist?	Yes
**Availability of data and materials**<br><br>All datasets and code on which the conclusions of the paper rely must be either included in your submission or deposited in publicly available repositories (where available and ethically appropriate), referencing such data using a unique identifier in the references and in the "Availability of Data and Materials" section of your manuscript.<br><br>Have you have met the above requirement as detailed in our Minimum	No

Powered by Editorial Manager® and ProduXion Manager® from Aries Systems Corporation

Standards Reporting Checklist?	
If not, please give reasons for any omissions below.<br><br>as follow-up to "**Availability of data and materials**<br><br>All datasets and code on which the conclusions of the paper rely must be either included in your submission or deposited in publicly available repositories (where available and ethically appropriate), referencing such data using a unique identifier in the references and in the "Availability of Data and Materials" section of your manuscript.<br><br>Have you have met the above requirement as detailed in our Minimum Standards Reporting Checklist?<br><br>"	All sequence data will be submitted to a publicly available repositories when this manuscript is acceptable for publication in GigaScience.

Powered by Editorial Manager® and ProduXion Manager® from Aries Systems Corporation

Manuscript
Click here to download Manuscript Introgressed Chinese haplotypes in French Large White_6.docx

Population genomic data reveal origin and phenotypic effect of Chinese haplotypes introgressed into European modern pigs

Hao Chen[§], Min Huang[§], Bin Yang, Zhongping Wu, Zheng Deng, Yong Hou, Jun Ren[*†], Lusheng Huang[*]

State Key Laboratory of Pig Genetic Improvement and Production Technology, Jiangxi Agricultural University, Nanchang, P.R. China.

[§] Both authors contributed equally to this study and should be considered co-first authors

[†]Current address: College of Animal Science, South China Agricultural University, 510642, Guangzhou, P.R. China

Correspondence author: Jun Ren, Lusheng Huang

State Key Laboratory of Pig Genetic Improvement and Production Technology

Jiangxi Agricultural University

Nanchang 330045, P. R. China.

Phone: 0086-791-83805967; Fax: 0086-791-83900189;

E-mail: renjunjxau@hotmail.com; lushenghuang@hotmail.com

Abstract

Pigs were domesticated independently from European and Asian wild boars nearly 10,000 years ago. Chinese indigenous pigs were historically introduced to improve Europe local pigs. However, the geographic origin and biological functions of introgressed Chinese genes in modern European pig breeds remain largely unknown. Here we explored whole-genome sequencing data from 266 Eurasian wild boars and domestic pigs to produce a fine-scale map of introgression between French Large White (FLW) and Chinese pigs. We show that FLW pigs had historical admixture with both South Chinese (SCN) and East Chinese (ECN) pigs 200–300 years ago. A set of SCN haplotypes are beneficial for improving disease resistance and those of ECN haplotypes are favorable for better reproductive performance in FLW pigs. Intriguingly, we found both human mediated and archaic introgression events at the *AHR* locus, at which the archaic haplotype contribute to increased fertility in both ECN and FLW pigs. This study advances our understanding of the development history of global domestic pigs and highlights the importance of artificial hybridization and natural archaic introgression in the formation of phenotypic characteristics in domestic animals.

Introduction

Integrated genomic and archaeological evidence have illuminated that wild boar (*Sus scrofa*) originated from the Islands of Southeast Asia about 5 million years ago and then dispersed throughout Eurasia. Approximately 1 million years ago, geographic isolation caused by glacial events hampered the continuous gene flow among Eurasian wild boars, causing European and Asian wild boars to differentiate from each other [1-4]. About 10,000 years ago, European and Asian wild boars were domesticated independently in the Near East and China, respectively [3, 5, 6]. After long-term artificial selection and natural selection, abundant genetic resources of domestic pigs appeared in China, accounting for about one-third of global breeds [7, 8]. Chinese pigs are distributed in diverse geographic regions and have different breed features. For example, Erhualian (EHL) and Meishan pigs in East China are known for their prolificacy, with a litter size of more than 15, and for their thick skin. Luchuan (LUC) and Bama pigs in South China have inferior reproductive performance (8–10 piglets per parity) and have thin skin and excellent heat resistance [7]. These pig breeds not only play a critical role in the Chinese pig industry but also have contributed to the development of international commercial breeds, such as the Large White (LW) [9, 10].

Over hundreds of years, Chinese pigs were introduced to Europe, mainly during three historical periods [7]. From 1685 to 1757, the Qing Dynasty set up four foreign trade ports: two in East China (Shanghai and Ningbo) and two (Zhangzhou and Guangzhou) in South China. Europe (especially England) had frequent trade with China through the four ports mainly via the East India Company. This raises the possibility that East Chinese (ECN) and South Chinese (SCN) pigs may have been transported to European countries during this period. From 1757 to 1841, only the Guangzhou port in South China was permitted for foreign trade, and a ban was imposed on maritime trade or intercourse with foreign countries in 1757. It is well documented that SCN pigs had been introduced to England for the

hybridization of local pigs during this period, contributing to the formation of Berkshire [9] and LW pigs [10]. In 1978, the Chinese government launched the reform and open-door policy. Since then, ECN pigs, including Meishan, Jinhua, and Jiaxing Black, have been introduced into France, America, and Japan for the development of prolific synthetic lines [7].

Recently, whole-genome re-sequencing analysis confirmed the human-mediated translocation of Chinese pigs into Europe, which provided genetic variations for the selective breeding of modern commercial LW pigs [11]. However, it remains unknown if SCN or ECN pigs or both were introduced to Europe, because previous studies used a limited number of Chinese pigs from different locations as a whole population. French Large White (FLW) pigs are known for their excellent reproductive performance. A remarkable genetic improvement of litter size has been witnessed in FLW pigs over the past decades, but the molecular mechanisms underlying the fecundity remain unclear, although the fecundity is speculated to be related to the recent introgression of highly prolific Chinese pigs such as ECN pigs [7]. Further studies are required to test this speculation.

In this study, we explored whole-genome sequencing data of 266 Eurasian pigs to show that both SCN and ECN haplotypes were introgressed into LW pigs ~200-300 years ago. Some of the introgressed haplotypes have been under preferential selection to improve fertility and immunity in FLW pigs. Interestingly, the prolificacy-associated *AHR* haplotype was likely introgressed from an archaic *Sus* population into ECN pigs via interspecies hybridization and was then introduced from ECN pigs into FLW pigs through human-driven transportation. These findings advance our understanding of the development history and genetic mechanisms underlying breed characteristics of global domestic pigs. Moreover, this study highlights the importance of artificial intraspecies crossbreeding and natural interspecies hybridization on the phenotypic characteristics of domestic animals.

Results

Whole-genome sequencing data

We obtained whole-genome sequencing data of 266 animals from 25 populations (**supplementary table S1**), including 36 highly prolific FLW pigs from the nucleus populations of two breeding companies. The 36 pigs were selected with their total number born (TNB) piglets of more than 19 and distant genetic relationship among each individual (**supplementary fig. S1**). High-depth re-sequencing was conducted on a Hiseq 2000 or 2500 sequencer (Illumina, USA). After filtering raw data (see Methods), we called 32.7 million single nucleotide polymorphisms (SNPs) from the 266 individuals. For the 28 LW pigs whose sequence data were retrieved from the public NCBI database (see Methods), we use the Illumina Porcine SNP60 chip [12] data set to identify their origin. We demonstrated that 14 individuals belonged to the American Large White (ALW) pig, and the other 14 individuals pertained to the Dutch Large White (DLW) pig (**supplementary fig.S2**).

Genetic differentiation between SCN and ECN pigs

Eurasian wild boars began to differentiate as early as ~ 1 million years ago [2, 3], and Chinese and European wild boars were independently domesticated about 10,000 years ago [1, 3]. The remarkable genetic differentiation between Chinese and Western pigs was reflected in the results of principal component analysis (PCA), phylogenetic analysis and admixture analysis (**fig. 1**). In the PCA analysis, the first principal component (PC1) accounted for 16.32% of the total eigenvalue (PC1 = 16.32%), which clearly separated the Chinese pig from the Western pig. The second principal component (PC2) showed the differentiation among Chinese pigs, especially between SCN and ECN pigs (PC2 = 3.78%, **fig. 1a**). In the neighbor joining tree between individuals (**fig. 1b**) and populations (**fig. 1c**),

Chinese and Western pigs defined two separate clades. For Chinese domestic pigs, SCN and ECN pigs formed two different branches. The clustering pattern was similar to the maximum likelihood tree revealed by the TreeMix analysis, in which two Sumatras wild boars, one *Sus barbatus*, one *Sus verrucosus*, one *Sus cebifrons*, one *Sus celebensis*, and one *Phacochoerus africanus* were treated as the outgroup (OUT), and the interpretation of the maximum likelihood tree reached 99.9% (**supplementary fig. S3**). In the admixture analysis, Chinese pigs and European pigs showed two distinct ancestral lineages when K = 2, although there were gene flows between the two groups, especially the North Chinese pig that clearly mixed with European pig lineages, whereas LW (including FLW) pigs showed signature of admixture with Chinese pigs. ECN pigs represented by Jinhua pigs and SCN pigs represented by Luchuan pigs appeared as the two ancestral lineages of Chinese pigs when K = 3 (**fig. 1d**). Altogether, these findings not only confirmed the independent domestication of Chinese and European pigs, but also unraveled that SCN pigs and ECN pigs have marked genetic differentiation and represent two ancient lineages of the Chinese domestic pig.

SCN and ECN pigs were introgressed into Europe between 220 and 310 years ago

To determine whether SCN and ECN pigs were introduced into Europe via human-mediated transportation, we performed relative identity-by-descent (rIBD) analysis using whole genome sequencing data (see Methods). We detected 5,107 and 5,024 50-kb regions with signatures of potential introgression from SCN or ECN pigs into FLW pigs, respectively (**figs. 2a, 2b, supplementary fig. S4**). The introgressed DNA from SCN and ECN pigs differed greatly in FLW pigs, with an overlap of only 6.0% introgression regions (**fig. 2c**) and 2.9% genes within the regions (**fig. 2d**). We performed Gene Ontology (GO) and Kyoto Encyclopedia of Genes and Genomes (KEGG) pathway enrichment analysis for the genes located in the introgressed regions. The genes within the regions of inferred introgression

with SCN pigs and ECN pigs were enriched in the immune-related signaling and fertility pathways, respectively (**fig. 2e**). We further used ALDER software [13] to estimate that the time of hybridization between FLW and SCN or ECN pigs was 220-310 years ago, which was consistent with the historical record stating that SCN pigs were deliberately transported into England at the onset of the first Industrial Revolution and contributed to the breeding of LW pigs [11]. In addition, these results support our hypothesis that ECN pigs were also introduced into Europe to improve productivity of local pigs between 1685 and 1757.

The introgressed *GOLM1-NAA35* haplotype from SCN pigs has been under selection to enhances the disease resistance of FLW pigs

We detected seven genomic regions with strong signature of introgression from SCN pigs in the genomes of FLW pigs (rIBD value >0.2; **supplementary table S2**). Two adjacent genes (3,511 bp apart), *GOLM1* and *NAA35*, were located in one of the seven regions (SSC10: 33.20–33.58 Mb). The *GOLM1* gene encodes a type II Golgi transmembrane protein, which is mainly synthesized in the rough endoplasmic reticulum, assists in processing proteins in the Golgi and is responsive to viral infections [14]. In 2016, Li *et al*. [15] reported that the *GOLM1-NAA35* locus markedly modulate the cytokine interleukin-6 (IL-6) production by human immune cells in response to multiple pathogens. Given the important role of the *GOLM1-NAA35* locus in disease resistance, we chose this locus for further study.

We first make a close examination on the rIBD results for a 2-Mb region encompassing the *GOLM1-NAA35* locus (SSC10: 33.20–33.58 Mb). We found that the frequency of shared IBD haplotypes between FLW and SCN pigs at the *GOLM1-NAA35* locus was significantly higher than those in the surrounding regions (**fig. 3a**). Moreover, we observed remarkably elevated genetic differentiation (F_{ST}) between FLW pigs and European wild boar in contrast to particularly decreased F_{ST} between FLW and SCN pigs in the *GOLM1-NAA35* region (**fig.**

3b). In addition, there were four main *GOLM1-NAA35* haplotypes in FLW pigs. Most individuals (32 out of 36) carried haplotypes similar to those of SCN pig. (**fig. 3c**).

Next, we used 3,447 SNPs in the *GOLM1-NAA35* region to construct the NJ tree (**supplementary fig. S5**). We found that most FLW pigs (n = 32) gathered with SCN pigs to form a branch that was separated from ECN pigs and European pigs, whereas only a small number of FLW pigs (n = 4) clustered with European pigs, which was in stark contrast to the genome-wide NJ-tree (**fig. 1a**). We further constructed a haplotype network using 298 SNPs at the *GOLM1-NAA35* locus (**fig. 3d**). We clearly identified the haplotype VII as being the main haplotype in the FLW pigs, which appeared 37 times in all populations, including 23 in FLW pigs, 8 in LW pigs, and 6 in SCN pigs. The SCN-major haplotype VIII and the haplotype VII differed by only four different sites, whereas the unique haplotypes (XIX, XXIII and X) of European wild boar and the haplotype VII differed by more than 180 sites (**supplementary fig. S6**). These results corroborate the historical introgression of SCN pigs into FLW pigs and illuminate that the haplotype VII at the *GOLM1-NAA35* locus in FLW pigs originated from SCN pigs.

We noted that the introgressed haplotype VII was present in other LW pigs at low frequencies but absent in other European domestic pigs. It is conceivable because all LW populations originated in England where SCN pigs were introduced during the first Industrial Revolution (early 19th century) [7]. Moreover, the introgressed haplotype appeared one time in European wild boars. Considering the outdoor grazing of early European pigs, we believe that European wild boars had admixture with European domestic pigs, after which this haplotype was introgressed from European domestic pigs into European wild boars.

The haplotype heatmap of the *GOLM1-NAA35* region showed that the SCN-originated haplotype VII was frequently present in FLW pigs (**fig. 3c**), which suggested that this haplotype may be selected in FLW pigs. To verify this hypothesis, we first compared the

linkage disequilibrium (LD) values (r^2) of the *GOLM1-NAA35* region and a upstream (3 Mb) region with the same size as the *GOLM1-NAA35* locus. We found that the LD level in the *GOLM1-NAA35* region of the FLW population ($r^2_{0.3}$= 192.3 kb) was significantly higher than that of all other populations (**supplementary fig. S7a)**, whereas the LD value ($r^2_{0.3}$) in the upstream region was only 17.3 kb, which was similar to most populations **(supplementary fig. S7b**). Subsequently, we performed LD analysis for 10,000 81.9-kb regions randomly sampled across the genomes of 36 FLW pigs (**supplementary fig. S7c**). We found that the LD value (r^2) in the *GOLM1-NAA35* region ranked in the top 2.6% of the 10,000 bootstrap results, which was a significant outlier (P = 0.02) and suggests that the introgressed *GOLM1-NAA35* haplotype likely underwent a preference selection in FLW pigs, resulting in a local increase of LD level in the target region. The XP-EHH analysis also showed the evidence of selection in the *GOLM1-NAA35* region in FLW pigs but not in other LW pigs (**fig. 3e**).

To examine whether the *GOLM1-NAA35* haplotypes are associated with serum IL-6 contents in FLW pigs, we collected venous blood from 54 healthy adult FLW sows at the same physiological stage and detected IL-6 levels in the serum of each individual using enzyme-linked immunoassay (ELISA) (**supplementary table S3**). Meanwhile, we defined the *GOLM1-NAA35* haplotypes for each individual using two tag SNPs and then tested the association between these haplotypes and IL-6 content. We found that individuals homozygously carrying the introgressed haplotype (*QQ*) had significantly higher IL-6 concentrations than heterozygotes (*Qq*) (P = 0.015, **fig. 3f**). Altogether, a sensible explanation for the introgression at the *GOLM1-NAA35* locus is that the *GOLM1-NAA35* haplotype were historically introgressed from SCN pigs into LW pigs and then have been under preferential selection to improve effective production of IL-6 levels in response to pathogens and consequently enhance resistance to infectious disease in FLW pigs.

Historically, South China was renowned as a land of plague with a humid and stuffy environment. It was popular for local infectious diseases including malignant malaria that caused high transmission and mortality rates before the Southern Song Dynasty (1127-1279 AD). The hostile environment imposed server physiological challenges on inhabits in South China [7]. Native inhabits like humans and pigs are believed to have evolved the adaptive mechanism to address the harsh environment likely via selection of immune-related genes during the long history of colonization. It is thus conceivable that those genes including *GOLM1-NAA35* within the introgression regions from SCN pigs are enriched in the immune-related signaling pathway. Interestingly, a recent genomic analyses unraveled a list of genes related to immune response under selection in southern Han Chinese, including *G6DP* associated with resistance to malaria [16].

The introgressed *KATNAL1* haplotype from ECN pigs is preferentially selected to increase the fertility of FLW boars

In FLW pigs, a 200-kb region on chromosome 11 (6.675-6.875 Mb) showed the strongest (the highest rIBD value) signal of admixture with ECN pigs, which contained only one gene, *KATNAL1*. *KATNAL1* regulates microtubule dynamics in testicular support cells, affecting the separation and binding of microtubules. Promoting the rapid reorganization of testicular support cell microtubule arrays is an essential process for spermatogenesis and male fertility [17]. Thus, *KATNAL1* plays an important role in spermatogenesis. Given the top introgression signal at the *KATNAL1* locus and the role of *KATNAL1* in boar fertility, we conducted an in-depth analysis focusing on the *KATNAL1* region using the same method as used for the *GOLM1-NAA35* locus.

We found that the frequency of the shared IBD haplotype between FLW and ECN pigs in the *KATNAL1* region was particularly higher than that in the surrounding segments (**fig.**

4a). There was a remarkable local increase of F_{ST} between FLW pigs and European wild boar and a particular decrease of F_{ST} between FLW pigs and ECN pigs in the *KATNAL1* region **(fig. 4b**). FLW pigs had four main haplotypes in this region. Most individuals (30 out of 36) carried haplotypes highly similar to the ECN haplotypes, and the others were similar to European wild boars and European domestic pigs (**fig. 4c**). Additionally, 30 FLW pigs and ECN pigs were clustered into one large clade while only six FLW pigs were grouped with European pigs in the NJ tree that was constructed with 529 SNPs in the *KATNAL1* gene (**supplementary fig. S8**). Meanwhile, we constructed a haplotype network using the 529 SNPs (**fig. 4d**) and analyzed nucleotide differences among different haplotypes (**supplementary fig. S9**). The most frequent haplotype (XXVII) appeared 57 times in the 266 tested individuals, including 35 in FLW pigs, 18 in ECN pigs, 2 in ALW pigs and 2 in SCN pigs. This haplotype and its closest ECN haplotype (XXV, five different sites, **supplementary fig. S9**) were divergent from the European pig haplotype groups (**fig. 4d**). These results further demonstrate that the *KATNAL1* haplotypes were introgressed from ECN pigs into FLW pigs.

We performed LD bootstrap sampling and XP-EHH analysis to detect the evidence of selection at the *KATNAL1* locus in FLW pigs. First, we compared the LD value (r^2) of the *KATNAL1* region and those of 10,000 randomly selected genomic regions with the same size of the *KATNAL1* gene (43.4 kb). We found that the LD level in the *KATNAL1* region ($r^2_{0.3}$= 437.5 kb) was a significant (P = 0.02) outlier, ranking in the top 2.5% of 10,000 bootstrap results (**supplementary fig. S10**). We also detected a significant selection signal at the *KATNAL1* locus in FLW pigs but not in other LW pigs using XP-EHH (**fig. 4e**). These results suggest that the introgressed *KATNAL1* haplotype from ECN pigs is preferentially selected in FLW pigs.

Given the important role of *KATNAL1* in male fertility, the fecundity of ECN pigs and historical selection for fecundity in FLW pigs, we speculated that the introgressed *KATNAL1* haplotype could contribute to the improvement of male reproductive performance and thus have underwent selection in FLW pigs since introgression. To test this hypothesis, we analyzed the association between the *KATNAL1* haplotypes and the FLW boar fertility that was represented by the average estimated breeding value (EBV) for TNB of mating sows. We detected a significant difference in boar fertility between 17 homozygous carriers of the introgressed haplotype (*QQ*) and 14 carriers of non-ECN pig haplotypes (*qq*) (P = 0.036; **fig. 4f**). The EBV for TNB (EBV-TNB) of *QQ* individuals was 0.018, with a difference of 0.32 (equates to an increase of 0.32 piglets born) compared with *qq* individuals. As TNB is a complex multi-locus trait, an increase of 0.32 piglets born is substantial for the current pig breeding programs. This indicates that the introgressed *KATNAL1* haplotype has been favored and intensively selected by breeders, contributing to the formation of excellent reproductive traits in FLW pigs.

AHR **haplotypes that associate with increased litter size were likely introgressed from ECN pigs into LW pigs**

In 2014, Bosse *et al.* [11] found that Chinese haplotypes in a 6.8-Mb region on chromosome 9 containing the *AHR* gene were introgressed into European pigs and were preferentially selected to increase fertility during the development of LW pigs. We also conducted a shared haplotype test (rIBD) between 121 Chinese pigs and 64 LW pigs in the 6.8-Mb region. We confirmed the presence of Chinese-derived haplotypes in European pigs including FLW pigs, with a strong introgression signal at the *AHR* locus (SSC9: 92.25–97.45 Mb) (**supplementary fig. S11**). To explore the geographic origin of the introgressed Chinese *AHR* haplotypes, we first constructed a phylogenetic tree of all sequenced individuals around the

AHR region, and surprisingly found that most of domestic pigs were clustered together with small genetic distance but were divergent from European and Asian wild boars (**supplementary fig. S12a**). We further reconstructed and visualized haplotypes around the *AHR* gene (95.5–95.65 Mb) and found that most haplotypes of LW pigs were highly similar to those of Chinese EHL pigs and Tibetan pigs (**fig. 5a**). In the NJ-tree of this region, 15 FLW pigs gathered with EHL pigs and Tibetan pigs, defining a branch distinct from other Chinese breeds (**supplementary fig. S12b**). In addition, FLW pigs and EHL pigs had the smallest F_{ST} value with the exception of other LW pigs (**supplementary fig. S12c**). Given the geographic distance between Tibet and Europe and the lack of any historical records describing the importation of Tibetan pigs into Europe, we argue that Chinese derived *AHR* haplotypes in FLW pigs were most likely introgressed from ECN pigs such as EHL pigs.

The *AHR* haplotype was introgressed into Chinese pigs via ancient interspecies hybridization

We noticed that the *AHR* haplotypes of most Chinese pigs were highly similar, but were distinct from those of Asian and European wild boars (**figs. 5a)**. Moreover, a large proportion of Chinese domestic pigs such as EHL pigs had a smaller nucleotide distance from the OUT population than from Asian wild boars in the *AHR* region (**supplementary fig. S13**); this was unexpected, as we know that these domestic pigs originated from wild boars. One possible explanation is that *AHR* haplotypes of many Chinese domestic pigs were not derived from Chinese wild boars but from another potentially extinct *Sus* species. To test this hypothesis, we constructed a haplotype network using 133 SNPs in the *AHR* gene (see Methods). Interestingly, we observed three distinct haplotype groups, one included haplotype of Asian wild boars and Chinese domestic pigs, one comprised those of European wild boars and European domestic pigs, and the other was defined by haplotypes of Eurasian domestic pigs

and OUT individuals (**fig. 5b**). In addition, the most frequent haplotype (XVI) appeared 100 times in all 266 sequenced individuals, including 30 in FLW pigs, 24 in other LW pigs, 18 in EHL pigs and 26 in Tibetan pigs. This haplotype had a close phylogenetic relationship with the OUT haplotype XII but was divergent from the major haplotypes (II and VII) of Eurasian wild boars (**fig. 5b**), a pattern expected under introgression. Note that the nucleotide difference between the haplotype XVI and the OUT haplotype XII was only 7, in contrast to 100 between haplotypes XVI and II and 93 between haplotypes XVI and VII (**fig. 5c**). These findings corroborate our assumption that the haplotype XVI was introgressed from a divergent archaic *Sus* population into the ancestors of Chinese domestic pigs via naturally occurring interspecies hybridization, then introduced from ECN pigs into European domestic pigs through human-mediated transportation about 200-300 years ago, and thus rarely existed in Eurasian wild boars.

To provide further evidence for the possible interspecies hybridization at the *AHR* locus, we selected 38 individuals from OUT, Asian wild boars (AWB), and ECN pigs (EHL as an example) to perform allele frequency difference analysis, and calculated the ratio of OUT SNPs in EHL pigs per window with a sliding window of 50 kb across the genome (see Methods). Of note, a total of 63 SNPs in the 50-kb window containing the *AHR* gene were potential archaic SNPs derived from the OUT population, accounting for 66% of total SNPs in this window. This ratio was the largest one in all 45,429 windows genome-wide (**fig. 5d**). Furthermore, we used an improved ABBA-BABA method (f_D) for gene flow analysis (see Methods). Interestingly, we detected an extreme outlier signal that the window containing the *AHR* gene had an f_D value greater than 0.8 corroborated by a significant outlier of the nucleotide distance (D_{xy}) between EHL pigs and AWB (**fig. 5e**). In addition, the nucleotide distance of EHL pigs versus AWB and FLW pigs versus European wild boars reached 2.05 and 2.48, respectively; whereas the nucleotide distance of EHL pigs versus OUT animals and

FLW pigs versus OUT animals were only 1.51 and 1.41, respectively (**supplementary fig. S13**). Altogether, our data strongly support the archaic introgression at the *AHR* locus.

We noted that the introgressed haplotype XVI was desirable for increasing the EBV-TNB of both FLW pigs (**fig. 5f**) and EHL pigs (**fig. 5g**). By genotyping the haplotype tag SNPs and one-way analysis of variance (see Methods), we found that homozygous carriers of the archaic *AHR* haplotype (XVI) had 0.24 higher EBV-TNB than heterozygous carriers (P = 0.001, **supplementary table S7**) in EHL pigs. Moreover, the introgressed archaic *AHR* haplotype was significantly associated with increased EBV-TNB of FLW sows with an additive effect value of 0.25 (P = 2.39e-05; **fig. 5f, supplementary table S6**), which was in agreement with the report of Bosse *et al.*[11]. Similar to *KATNAL1* and *GOLM1-NAA35* regions, the LD value of FLW pigs in the *AHR* gene region ranked in the top 7% (significant outlier) of all 10,000 bootstrap values (P = 0.03, **supplementary fig. S14**). We also detected a significant iHS selection signal within the FLW pig population (**supplementary fig. S15**). These findings enable us to conclude that the archaic *AHR* haplotype has been under a preferential selection to improve the fertility of FLW pigs.

Discussion

European and Asian domestic pigs were independently domesticated from European and Asian wild boars, respectively, nearly 10,000 years ago [3, 5, 6]. In this study, population genetics analyses confirmed striking genetic differences between Chinese and European domestic pigs and uncovered obvious genetic differentiation between SCN and ECN pigs, which represent two ancestral lineages of Chinese pigs. Of note, we identified Chinese haplotypes in FLW pigs, which were introgressed from both SCN and ECN pigs. We inferred that the introgression events occurred 220–310 years ago, which is in accordance with historical records that SCN pigs were transported to the England through the Guangzhou port

during the first Industrial Revolution [7]. Our results also supported the speculation that ECN pigs were introduced into Europe likely through the Shanghai and Ningbo ports in the decades before the Qing Dynasty imposed the ban on the sea in 1757. Thus, we believe that both SCN and ECN pigs were introduced to Europe to improve production performance of local breeds, contributing to the development of modern European commercial pig breeds. Taking the *GOLM1-NAA35* and *KATNAL1* loci as examples, the introgressed *GOLM1-NAA35* haplotype from SCN pigs is beneficial for improving disease resistance in FLW pigs, and the introgressed *KATNAL1* haplotype from ECN pigs is favorable for boar fertility and provides genetic variations for the development of high-fecundity FLW pigs. These findings not only advance our understanding of the breeding history of modern European commercial pig breeds but also shed insights into the genetic mechanisms underlying economically important traits in pigs.

In recent years, emerging reports have shown that interspecies hybridization played an important role in adaptive evolution of mammals. For example, the Denisova-like *EPAS1* haplotype help Tibetans to adapt to the high-altitude hypoxia environment [18]. Admixture with yak enabled Tibetan cattle to quickly obtain favorable *EGPN1* alleles for high-altitude adaptation [19]. We reported an archaic adaptive introgression on the X chromosome that contributed to the adaptation of North Chinese pigs to high-latitude cold environments [20]. Here, we show that the *AHR* haplotype associated with increased sow litter size was derived from an archaic population. It was first introgressed into Chinese pigs via interspecies hybridization. Then it was introduced from ECN pigs into European pigs such as Large White through human-mediated transportation and hybridization some 200–300 years ago. It has further experienced preferentially selection presumably during the past decades and is present at high frequency in FLW pigs, contributing to the improvement of the reproductive performance in this breed. Thus, this study provides another example of the archaic adaptive

introgression in domestic animals. It also shows that both naturally occurring interspecies hybridization and human-driven crossbreeding play important roles in the development of global pig breeds, illustrating a complex breeding history of domestic pigs.

Materials and Methods

Samples

All procedures used for this study and involving animals were in compliance with guidelines for the care and utility of experimental animals established by the Ministry of Agriculture of China. The ethics committee of Jiangxi Agricultural University approved this study. This study utilized genome-wide re-sequencing data from 266 animals (**supplementary table S1**), of which 153 pigs were re-sequenced for this study and 113 genome sequence data were downloaded from the public database (Registration Nos. ERP001813 [21], PRJEB9922 [22], and SRP047260 [23]). Among the 153 pigs, 36 were FLW sows and were collected from the Guangdong WENS Food Company (24 individuals) and Jiangxi Lvhuan Animal Husbandry Company (12 individuals). The 36 FLW sows were selected according to the following criteria. First, we calculated the relationship coefficients of all individuals in the nucleus populations of the two companies using the DMU software [24] and pedigree records. Then we selected sows with a small relationship coefficient and excellent litter sizes (TNB more than 16). Finally, we chose 36 prolific individuals with distant kinship according to the phylogenetic relationship network constructed by Cytoscape v3.2.1 [25] (**supplementary fig. S1**). In total, there were 27 wild boars from China and Europe, 7 outgroup individuals, 121 pigs from Chinese indigenous breeds, and 111 pigs from European commercial breeds. According to the geographic distribution, Chinese domestic pigs were divided into ECN (37) pigs, SCN (20) pigs, SWCN (36) pigs, and NCN (28) pigs (see **supplementary table S1** for details). In addition, whole-genome sequence data of 28 LW pigs were downloaded from the

public database; 14 individuals submitted by Seoul National University [23] and another 14 individuals submitted by Wageningen University [21]. To identify the source of these 28 LW pigs, we downloaded the Illumina 60K chip SNP data set of 76 LW pigs [26], including 20 Dutch Large White pigs (NLW), 16 Danish Large White pigs (DLW), 20 Chinese Large White pigs (CLW), and 20 American Large White pigs (ALW). Next, we retrieved the same 60K chip SNPs from the whole-genome sequence data sets of the 28 LW pigs. We filtered out SNPs with an MAF less than 0.05, a call rate less than 90%, and a LD (r^2) value more than 0.3 using PLINK v1.9 [27], and we performed PCA and NJ-tree analyses using the remained SNPs to identify the origin of the 28 LW pigs (**supplementary fig. S2**).

Whole-genome sequencing and SNP calling

We extracted genomic DNA from the ear tissues of 153 pigs using a routine phenol/chloroform protocol, and eligible samples were delivered to the Novogene company (Beijing, China). Sequencing was performed on Hiseq 2000 or 2500 instruments (Illumina, USA). The sequencing library was constructed with 125 bp paired ends (PE125), a 500 bp average insert fragment, and a fragment less than 800 bp. The genome sequencing coverage of each individual was at least 20× with a minimum data of 60 G.

Quality control: We generated the raw sequencing data from Hiseq sequencing platform using raw image data. We obtained clean data to perform a downstream analysis according to the following steps: (1) remove the linker sequence, (2) retain reads with Q20 more than 90% (the probability of base recognition correct rate higher than 99%) and Q30 more than 85% (the probability of base recognition correct rate higher than 99.9%)[28], (3) cull short repeat DNA segments, and (4) filter reads with three consecutive "N".

Mutation detection: We established the reference genome index of Sscrofa 10.2 [6] using an index function in BWA v0.7.12 [29]. We blasted paired-end reads against the index

using aln algorithm from BWA and obtained binary bam files from sam files by SAMtools v1.4 [30]. We used samblaster v0.1.22 [31] to reject redundancy information and calculated the alignment rate between re-sequencing data and the reference genome, as well as coverage and sequencing depth. We sorted binary bam files via GATK v3.7 [32]. We used the HaplotypeCaller function for mutation detection across each chromosome of each individual and obtained an SNP data set of the 266 individuals by deleting InDel information. We filtered out SNPs with an MAF less than 0.01 and a call rate less than 90% using PLINK v1.9 [27]. We used the remaining 32.7 million SNPs in the data set for subsequent statistical analysis.

Population genetic analysis

First, we generated the SNP data set with an MAF more than 0.05 and a call rate more than 90% from autosomal SNPs of 259 pigs (*Sus scrofa*) excluding seven OUT individuals. Second, we pruned SNPs with an LD (r^2) decay more than 0.3 in each window with 50 SNPs using command indep-pairwise (50 10 0.3) in PLINK v1.9 [27]. Then four principal components of each individuals were estimated using --pca command in the GCTA software [33]. Average shared allele (1-Dst) distance matrix among individuals was constructed using command --distance-matrix in PLINK v1.9. A rootless NJ tree was constructed through phylip v3.69 [34] and was visualized with FigTree v1.42. We also explored the unbiased estimation method proposed by Weir and Cockerham to calculate the genetic differentiation (F_{ST}[35]) matrix among 14 Chinese pig breeds and 6 European pig breeds using --fst command in PLINK v1.9 ([27]. Then, we constructed the interbreed NJ tree using phylip v3.69 [34]. ADMIXTURE [36] was used to estimate ancestral lineage composition under the default parameter. First, we removed OUT and a population with fewer than five individuals. Then we randomly selected six individuals from the remaining 21 populations and filtered

out SNPs with MAF less than 0.05, LD (r^2) more than 0.3, and call rate less than 90%. Finally, we used a data set with 125 individuals and 658,601 SNPs to analyze the ancestral lineage composition pattern. In addition, we utilized TreeMix v1.12 [37] to infer the genetic differentiation among populations. We set OUT as the outgroup population, excluding populations with fewer than six samples and SNPs with MAF less than 0.05 and a call rate less than 90%. We used the data set with 19,282,590 SNPs to estimate genetic differentiation among 21 populations under no migration events via TreeMix v1.12 [37].

Introgression analysis

We detected the introgression signals between Chinese pigs (ECN and SCN pigs) and FLW pigs by sharing IBD frequency proposed by Bosse *et al* [11]. First, we used the data set with 266 individuals and approximately 20 million SNPs to phase haplotypes using fastPhase function [38] in Beagle v4.0 and to detect IBD fragments in each individual by fastIBD function [39]. Then we divided the whole genome into numbers of 50-kb windows (25 kb sliding) and calculated shared IBD haplotype numbers between two populations (FLW vs. European wild boars (EWB), FLW vs. ECN, and FLW vs. SCN) in each window. We phased the haplotypes and detected the IBD regions independently for 10 times and then normalized the IBD values (nIBD). The nIBD values ranged from 0 (no shared IBD detected) to 1 (all individuals shared the IBD haplotype). Finally, we used the rIBD (relative frequency of IBD) statistic to measure the shared IBD between FLW pigs and SCN or ECN pigs, respectively ($rIBD_{FLW\text{-}SCN} = nIBD_{FLW\text{-}SCN} - nIBD_{FLW\text{-}EWB}$, $rIBD_{FLW\text{-}ECN} = nIBD_{FLW\text{-}ECN} - nIBD_{FLW\text{-}EWB}$), where a positive rIBD indicates potential introgression and 1% and 5% empirical distribution in the far right tail were set as the significance thresholds. For genomic regions showing strong rIBD introgression signal in FLW pigs, we further estimated F_{ST} between FLW pigs and European wild boars, as well FLW pigs and Chinese pigs (SCN pigs or ECN pigs),

respectively. We also constructed a haplotype network at the *GOLM1-NAA3*, *KATNAL1* and *AHR* loci using "haploNet" command in R package "pegas" [40]. We used ALDER v1.0.3 [13] to estimate admixture time between populations. In short, we used the "convert" function in EIGENSTRAT [41] to convert the data format. We set FLW as a mixed population, EWB and SCN as one reference population, and EWB and ECN as another reference population. We set five years as one generation to estimate admixture time between EWB and SCN as well between EWB and ECN.

Signature of selection

We used the data set that excluded SNPs with an MAF less than 0.05 and a call rate less than 90% in the whole-genome SNPs data set of 36 FLW pigs to calculate correlation coefficient (r^2) of each SNP pairs in a target region using command --r2 inter-chr --ld-window-r2 0 in PLINK v1.9 [40], and we used the average r^2 as the LD value in the region. Meanwhile, we randomly selected 10,000 regions with the same size of the target region across the genome, and we calculated the average r^2 of each region in the 36 FLW pigs. Finally, we visualized the density curve of 10,000 bootstrap values using the R language code. Furthermore, we used commands --ihs [42] and --xpehh [43] in the selscan [44] software to detect signatures of selection under 50-kb windows with a step size of 25 kb in FLW pigs.

Archaic introgression test

We used an improved f_D method [45] under ABBA-BABA statistic to detect the potential archaic introgression in the *AHR* region between the OUT population and EHL pigs. The calculation formulas was as follows:

$$C_{ABBA}(i) = (1 - p_{i1})p_{i2}p_{i3}(1 - p_{i4})$$

$$C_{BABA}(i) = (1 - p_{i1})p_{i2}(1 - p_{i3})p_{i4}$$

$$S(p_1, p_2, p_3, O) = \sum [C_{ABBA}(i) - C_{BABA}(i)]$$

$$f_D = \frac{S(p_1, p_2, p_3, O)}{S(p_1, p_D, p_D, O)}$$

where p_{ij} indicates the *ith* frequency of the derived allele in the *jth* population, S represents sum of difference between ABBAs and BABAs, and p_D means populations with higher frequency of the derived allele (P2 or P3). According to the NJ tree, Asian wild boars, EHL, and five OUT individuals (two Sumatras, one *Sus barbatus*, one *Sus verrucosus*, and one *Sus* cebifrons) and one OUT individual (*Phacochoerus africanus*) were set as P1, P2, P3, and O, respectively.

Nucleotide differences within (D_x) and among (D_{xy}) populations were calculated using follow formulas [20]:

$$D_x = \frac{2}{n_x(n_x - 1)l} \sum_{i=1}^{n_x - 1} \sum_{j=i+1}^{n_x - 1} k_{ij}$$

$$D_{xy} = \frac{2}{n_x n_y l} \sum_{i=1}^{n_x} \sum_{j=1}^{n_y} k_{ij}$$

where k_{ij} indicates difference number of haplotype alleles between the *ith* allele the *jth* allele in target region, n_x and n_y represent number of haplotypes in population *x* and *y*, and *l* indicates the number of bases that are valid in the target area (the number of bases other than N in the reference sequence). In addition, 38 individuals were selected from OUT (7), Asian wild boar (10), and Erhualian populations (21) and pruned SNPs with MAF less than 0.05 and call rate less than 90%, leaving 14,333,796 SNPs. We used 50-kb windows with a sliding size of 25 kb filtering windows with less than 10 SNPs to calculate the allele ratio ($r_D = n_i/n_o$) in each window, where n_o indicates the number of SNPs with an allele frequency more than 0.7 in each window in OUT, and n_i represents the number of SNPs

with an allele frequency more than 0.6 in EHL pigs as well less than 0.15 in Chinese wild boars. The r_D of the *AHR* region is shown using a probability density curve.

Haplotype association analysis

The *GOLM1-NAA35* locus: We detected the serum IL-6 levels in 54 mature FLW sows at the age of 2-2.5 years from the same farm using the Porcine IL-6 ELISA Kit (Shanghai Keshun Biological Technology, China). The concentration of each individual was determined from the averaged repeat of three trials per individual. Meanwhile, we selected two tag SNPs to distinguish the introgressed haplotypes (VII and VIII) from the other haplotype in the *GOLM1-NAA35* region in FLW pigs (**fig. 3e**). The tag SNPs were genotyped by Sanger sequencing PCR products amplified with specific primers (**supplementary table S3**). Student's *t*-test was used to detect the association between haplotypes and the serum IL-6 concentrations (log2 (IL-6 values)).

The *KATNAL1* locus: We collected 765 FLW sows and 31 FLW boars from Jiangxi Lvhuan Farming Group. First, we filtered parities with litter size less than five piglets. Then we set estrus, year, season, parity and pregnancy duration as fixed effect, and mating boars and random sow effects as random effects; and estimated EBV for TNB of 765 FLW pigs via the DMU software [24] and pedigree information. Next, we genotyped eight tagged SNPs to distinguish each *KATNAL1* haplotype in the 31 FLW boars by PCR amplification and Sanger sequencing with primers listed in **supplementary table S4**. We denoted the introgressed XXVII haplotype from ECN pigs as *Q* (**fig. 4e**) and the other haplotypes as *q* (**supplementary table S5**). Finally, we used Student's *t*-test to test the association between *KATNAL1* haplotypes and the average EBV-TNB of mating sows of the 31 FLW boars.

The *AHR* locus: We genotyped two tagged SNPs representing the *AHR* haplotypes for 344 FLW sows by PCR amplification and Sanger sequencing with primers listed in

supplementary table S6. We identified 230 *QQ* sows homozygous for the introgressed haplotype, 36 *Qq* sows and 78 *qq* sows absent from the introgressed haplotypes (**supplementary table S6**). Then we tested the association between the *AHR* haplotypes and EBV-TNB of the 344 sows using single-factor analysis of variance. Furthermore, we collected 221 Erhualian sows with multiparity records from Jiangsu Provence and calculated EBV-TNB of these sows using the DMU software and pedigree information as mentioned above. We genotyped a tag SNP in the *AHR* region by Sanger sequencing PCR products with specific primers (**supplementary table S7**). We detected 176 *QQ* sows homozygous for the introgressed haplotype and 45 heterozygous (Qq) sows. We used Student's *t*-test to examine the association between *AHR* haplotypes and EBV-TNB in Erhualian sows.

Author Contributions

J.R. and L.H. designed the study and analyzed data. J.R., H.C. and L.H. wrote the paper. H.C., M.H., and B.Y. performed bioinformatic analyses. H.C., M.H., Z.D. Z.W. and Y.H. collected data and performed sequencing and genotyping experiments.

Competing financial interests

The authors declare no competing financial interests

Acknowledgement

This study is supported by Natural Science Foundation of China (31525023) and National Key Research Project of China (2016ZX08006-5).

References

1. Giuffra E, Kijas JM, Amarger V, Carlborg O, Jeon JT and Andersson L. The origin of the domestic pig: independent domestication and subsequent introgression. Genetics. 2000;154 4:1785-91.
2. Kijas JM and Andersson L. A phylogenetic study of the origin of the domestic pig estimated from the near-complete mtDNA genome. Journal of Molecular Evolution. 2001;52 3:302-8.
3. Larson G, Dobney K, Albarella U, Fang M, Matisoosmith E, Robins J, et al. Worldwide phylogeography of wild boar reveals multiple centers of pig domestication. Science. 2005;307 5715:1618-21.
4. Frantz LA, Schraiber JG, Madsen O, Megens HJ, Bosse M, Paudel Y, et al. Genome sequencing reveals fine scale diversification and reticulation history during speciation in Sus. Genome Biol. 2013;14 9:R107. doi:10.1186/gb-2013-14-9-r107.
5. Frantz L, Meijaard E, Gongora J, Haile J, Groenen MAM and Larson G. The evolution of Suidae. Annual Review of Animal Biosciences. 2016;4 1:61.
6. Groenen MA, Archibald AL, Uenishi H, Tuggle CK, Takeuchi Y, Rothschild MF, et al. Analyses of pig genomes provide insight into porcine demography and evolution. Nature. 2012;491 7424:393.
7. Wang L, Chan YF, Russell P, Mauceli E, Johnson J, Swofford R, et al. Animal Genetic Resources in China: pigs (ed. China National Commission of Animal Genetic Resources). China Agriculture Press; 2011.
8. Rischkowsky B and Pilling D. The State of the World's Animal Genetic Resources for Food and Agriculture (Food and Agriculture Organization (FAO)). 2007.
9. Briggs HM and Briggs DM. Modern Breeds of Livestock. 4th ed. Macmillan Publishing Co.,Inc.; 1980.
10. Zhang W. Introduction to Large White pigs. SWINE PRODUCTION. 2011; 1:61-4.

11. Bosse M, Megens HJ, Frantz LA, Madsen O, Larson G, Paudel Y, et al. Genomic analysis reveals selection for Asian genes in European pigs following human-mediated introgression. Nat Commun. 2014;5:4392. doi:10.1038/ncomms5392.

12. Ramos AM, Crooijmans RP, Affara NA, Amaral AJ, Archibald AL, Beever JE, et al. Design of a high density SNP genotyping assay in the pig using SNPs identified and characterized by next generation sequencing technology. PLoS One. 2009;4 8:e6524. doi:10.1371/journal.pone.0006524.

13. Loh PR, Lipson M, Patterson N, Moorjani P, Pickrell JK, Reich D, et al. Inferring admixture histories of human populations using linkage disequilibrium. Genetics. 2013;193 4:1233.

14. Kladney RD, Bulla GA, Guo L, Mason AL, Tollefson AE, Simon DJ, et al. GP73, a novel Golgi-localized protein upregulated by viral infection. Gene. 2000;249 1–2:53-65.

15. Li Y, Oosting M, Deelen P, Ricañoponce I, Smeekens S, Jaeger M, et al. Inter-individual variability and genetic influences on cytokine responses to bacteria and fungi. Nature Medicine. 2016;22 10:1192.

16. Liu S, Huang S, Chen F, Zhao L, Yuan Y, Francis SS, et al. Genomic Analyses from Non-invasive Prenatal Testing Reveal Genetic Associations, Patterns of Viral Infections, and Chinese Population History. Cell. 2018;175 2:347-59 e14. doi:10.1016/j.cell.2018.08.016.

17. Smith LB, Milne L, Nelson N, Eddie S, Brown P, Atanassova N, et al. KATNAL1 Regulation of Sertoli Cell Microtubule Dynamics Is Essential for Spermiogenesis and Male Fertility. Plos Genetics. 2012;8 5:e1002697.

18. Huertasánchez E, Jin X, Bianba Z, Peter BM, Vinckenbosch N, Liang Y, et al. Altitude adaptation in Tibetans caused by introgression of Denisovan-like DNA. Nature. 2014;512 7513:194-7.

19. Wu DD, Ding XD, Wang S, Wójcik JM, Zhang Y, Tokarska M, et al. Pervasive introgression facilitated domestication and adaptation in the Bos species complex. Nature ecology & evolution. 2018.

20. Ai H, Fang X, Yang B, Huang Z, Chen H, Mao L, et al. Adaptation and possible ancient interspecies introgression in pigs identified by whole-genome sequencing. Nat Genet. 2015;47 3:217-25. doi:10.1038/ng.3199.

21. Rubin CJ, Megens HJ, Martinez Barrio A, Maqbool K, Sayyab S, Schwochow D, et al. Strong signatures of selection in the domestic pig genome. Proc Natl Acad Sci U S A. 2012;109 48:19529-36. doi:10.1073/pnas.1217149109.

22. Frantz LA, Schraiber JG, Madsen O, Megens HJ, Cagan A, Bosse M, et al. Evidence of long-term gene flow and selection during domestication from analyses of Eurasian wild and domestic pig genomes. Nature Genetics. 2015;47 10:1141.

23. Sunjin M, Tae-Hun K, Kyung-Tai L, Woori K, Taeheon L, Si-Woo L, et al. A genome-wide scan for signatures of directional selection in domesticated pigs. BMC Genomics,16,1(2015-02-25). 2015;16 1:1-12.

24. Madsen P, Sørensen P, Su G, Damgaard LH, Thomsen H and Labouriau R. DMU - a package for analyzing multivariate mixed models. In: *Proceedings of the 8th World Congress on Genetics Applied to Livestock Production, Belo Horizonte, Minas Gerais, Brazil, 13-18 August, 2006* 2014, pp.27-11.

25. Shannon P, Markiel A, Ozier O, Baliga NS, Wang JT, Ramage D, et al. Cytoscape: A Software Environment for Integrated Models of Biomolecular Interaction Networks. Routledge; 2003.

26. Yang B, Cui L, Perez-Enciso M, Traspov A, Crooijmans R, Zinovieva N, et al. Genome-wide SNP data unveils the globalization of domesticated pigs. Genet Sel Evol. 2017;49 1:71. doi:10.1186/s12711-017-0345-y.

27. Chang CC, Chow CC, Tellier LC, Vattikuti S, Purcell SM and Lee JJ. Second-generation PLINK: rising to the challenge of larger and richer datasets. Gigascience. 2015;4:7. doi:10.1186/s13742-015-0047-8.

28. Ewing B and Green P. Base-calling of automated sequencer traces using phred. II. Error probabilities. Genome Res. 1998;8 3:186-94.

29. Li H and Durbin R. Fast and accurate short read alignment with Burrows-Wheeler transform. Bioinformatics. 2009;25 14:1754-60. doi:10.1093/bioinformatics/btp324.

30. Li H. A statistical framework for SNP calling, mutation discovery, association mapping and population genetical parameter estimation from sequencing data. Bioinformatics. 2011;27 21:2987-93. doi:10.1093/bioinformatics/btr509.

31. Faust GG and Hall IM. SAMBLASTER: fast duplicate marking and structural variant read extraction. Bioinformatics. 2014;30 17:2503-5. doi:10.1093/bioinformatics/btu314.

32. McKenna A, Hanna M, Banks E, Sivachenko A, Cibulskis K, Kernytsky A, et al. The Genome Analysis Toolkit: a MapReduce framework for analyzing next-generation DNA sequencing data. Genome Res. 2010;20 9:1297-303. doi:10.1101/gr.107524.110.

33. Yang J, Lee SH, Goddard ME and Visscher PM. GCTA: a tool for genome-wide complex trait analysis. Am J Hum Genet. 2011;88 1:76-82. doi:10.1016/j.ajhg.2010.11.011.

34. Cummings MP. PHYLIP (phylogeny inference package). Dictionary of Bioinformatics and Computational Biology. 2004.

35. Weir BS and Cockerham CC. Estimating F-Statistics for the Analysis of Population Structure. Evolution. 1984;38 6:1358-70. doi:10.1111/j.1558-5646.1984.tb05657.x.

36. Alexander DH, Novembre J and Lange K. Fast model-based estimation of ancestry in unrelated individuals. Genome Res. 2009;19 9:1655-64. doi:10.1101/gr.094052.109.

37. Pickrell JK and Pritchard JK. Inference of population splits and mixtures from genome-wide allele frequency data. PLoS Genet. 2012;8 11:e1002967. doi:10.1371/journal.pgen.1002967.

38. Browning SR and Browning BL. Rapid and accurate haplotype phasing and missing-data inference for whole-genome association studies by use of localized haplotype clustering. Am J Hum Genet. 2007;81 5:1084-97. doi:10.1086/521987.

39. Browning BL and Browning SR. A fast, powerful method for detecting identity by descent. Am J Hum Genet. 2011;88 2:173-82. doi:10.1016/j.ajhg.2011.01.010.

40. Paradis E. pegas: an R package for population genetics with an integrated-modular approach. Bioinformatics. 2010;26 3:419-20. doi:10.1093/bioinformatics/btp696.

41. Price AL, Patterson NJ, Plenge RM, Weinblatt ME, Shadick NA and Reich D. Principal components analysis corrects for stratification in genome-wide association studies. Nat Genet. 2006;38 8:904-9. doi:10.1038/ng1847.

42. Voight BF, Kudaravalli S, Wen X and Pritchard JK. A map of recent positive selection in the human genome. PLoS Biol. 2006;4 3:e72. doi:10.1371/journal.pbio.0040072.

43. Sabeti PC, Varilly P, Fry B, Lohmueller J, Hostetter E, Cotsapas C, et al. Genome-wide detection and characterization of positive selection in human populations. Nature. 2007;449 7164:913-8. doi:10.1038/nature06250.

44. Szpiech ZA and Hernandez RD. selscan: An Efficient Multithreaded Program to Perform EHH-Based Scans for Positive Selection. Molecular Biology & Evolution. 2014;31 10:2824-7.

45. Martin SH, Davey JW and Jiggins CD. Evaluating the use of ABBA-BABA statistics to locate introgressed loci. Mol Biol Evol. 2015;32 1:244-57. doi:10.1093/molbev/msu269.

Figure legends

Fig. 1. Population relationship and structure. (**a**) Neighbor-joining (NJ) tree based on an identity-by-state matrix among individuals. (**b**) Principal component analysis (PCA) of Chinese and European pigs. ECN, East Chinese pigs; NCN, North Chinese pigs; SCN, South Chinese pigs; SWCN, Southwest Chinese pigs; EUD, European domestic pigs. (**c**) NJ tree based on a Fst matrics among populations. (**d**) Population structure of Chinese and European pigs revealed by the ADMIXTURE analysis. MIN, Min pigs; HT, Hetao pigs; LWH, Laiwu pigs; EHL, Erhualian pigs; MS, Meishan pigs; JH, Jinhua pigs; GST, Tibetan pigs (gansu)； SCT, Tibetan pigs (Sichuan); YNT, Tibetan pigs (Yunnan); TT, Tibetan pigs (Tibet); WZS, Wuzhishan pigs; LUC, Luchuan pigs; BMX, Bamaxiang pigs; XIANG, Xiang pigs; AWB, Asian wild boars; OUT, outgroup; EWB, European wild boars; HMP, Hampshire; DU, Duroc; LR, Landrace; PI, Pietrain; WDU, White Duroc; WLW, Dutch Large White pigs; KLW, Korea Large White pigs; FLW, French Large White pigs.

Fig. 2. Introgressed Chinese haplotypes in French Large White pigs. (**a**) Manhattan plot of rIBD values between French Large White (FLW) and South Chinese (SCN) pigs (positive value) or European wild boars (EWB) (negative value). The red dashed line indicates the top 5% significance threshold. (**b**) Manhattan plot of rIBD values between FLW and East Chinese (ECN) pigs (positive value) or EWB (negative value). (**c**) Venn diagram of introgressed DNA (50 Kb windows) from SCN and ECN pigs in FLW pigs. (**d**) Venn diagram of genes in the introgressed regions from SCN and ECN pigs in FLW pigs. (**e**) Significantly enriched GO processes and KEGG pathways of introgressed genes in the introgressed regions from SCN and ECN pigs under selection in FLW pigs.

Fig. 3. Introgression at the *GOLM1-NAA35* locus. (**a**) rIBD values in a 2-Mb region harboring the *GOLM1-NAA35 g*ene. The brown dashed line indicates the 5% threshold line, and the *GOLM1-NAA35* region is indicated by grey dashed lines. (**b**) Genetic differentiation index (F_{ST}) between French Large White (FLW) and European wild boar (EWB) or South Chinese (SCN) pigs. (**c**) Haplotype heatmap in the *GOLM1-NAA35* region. Major and minor alleles in FLW pigs are indicated by beige and light blue, respectively. (**d**) Haplotype network in the *GOLM1-NAA35* region. Each circle represents a haplotype, and the size of the circle is proportion to the haplotype frequency. The line width and length represent the difference between haplotypes. Different colors represent pigs from different geographical regions. OUT, outgroup; SWCN, Southwest Chinese pigs; NCN, North Chinese pigs; AWB, Asian (Chinese) wild boars; ECN, East Chinese pigs; EUD, European domestic pigs. (**e**) Selection signals in the *GOLM1-NAA35* region unraveled by the XP-EHH analysis between FLW and other Large White pigs. The brown dashed line indicates the 5% threshold line. (**f**) Serum interleukin 6 (IL-6) contents of FLW pigs homozygous (QQ) or heterozygous (Qq) for the introgressed *GOLM1-NAA35* haplotypes. Student's t-test was employed to compute the *P*-value ($P = 0.015$).

Fig. 4. Introgression at the *KATNAL1* locus. (**a**) rIBD values in a 2-Mb region encompassing the *KATNAL1* gene. The brown dashed line indicates the 5% threshold line, and the *KATNAL1* region is indicated by grey dashed lines. (**b**) Genetic differentiation index (F_{ST}) between French Large White (FLW) and European wild boar (EWB) or East Chinese (ECN) pigs. (**c**) Haplotype heatmap in the *KATNAL1* region. Major and minor alleles in FLW pigs are indicated by beige and light blue, respectively. (**d**) Haplotype network in the *KATNAL1* region. The legend is the same as in Figure 3. (**e**) Selection signals unraveled by the XP-EHH analysis between FLW and other Large White pigs. The brown dashed line

indicates the 5% threshold line. (**f**) Estimated breeding values for total number of piglets born (TNB EBV) of FLW sows that mated with FLW boars homozygous (QQ) or heterozygous (Qq) for the introgressed haplotypes. Student's t-test was employed to compute the *P*-value (*P* = 0.036).

Fig. 5. Archaic introgression at the *AHR* locus. (**a**) Haplotype heatmap in a 150-kb region on chromosome 9 (SSC9: 95.5-95.65 Mb). The *AHR* region is indicated by a red box. Major and minor alleles in FLW pigs are indicated by beige and light blue, respectively. (**b**) *AHR* haplotype network. Each pie chart represents one unique haplotype, and the radius of the pie chart is proportional to the five times of $\log_{10}$(number of chromosomes with that haplotype). The width and length of the edges are proportional to the $\log_2$(number of pairwise differences between the joined haplotypes) plus one, and the thinnest edge represents a difference of one mutation. Three different background colors represent three different haplotype groups. Different colors represent pigs from different geographical regions. The full names of pig codes are given in the legend of Figure 3. **(c**) Haplotype difference between each *AHR* haplotype. (**d**) Distribution of the potential archaic SNPs. At these SNPs, the frequency difference between Erhualian and Chinese wild boars is greater than 0.45, and that between Erhualian pigs and outgroup animals is less than 0.1. The x-axis shows the ratio of the potential archaic SNPs in each 50-kb window, and the y-axis indicates the number of windows. The red line marks the the ratio of the potential archaic SNPs in the window harboring the *AHR* gene. (**e**) Distribution of gene flow (f_D) and nucleotide distance (Dxy) statistics within nonoverlapping 50-kb windows across the genome. Dxy values between Erhualian pigs and Chinese wild boars are shown in the x-axis and f_D in the y-axis. The red dot, an extreme outlier, represents the window in which the *AHR* gene is located. (**f**) French Large White sows carrying the homozygous archaic *AHR* haplotype show significantly (*P* =

2.39×10^{-5}) lower estimated breeding values for total number born EBV (TNB_EBV), compared with those who do not carry the archaic haplotype. (**g**) Erhualian sows homozygously carrying the archaic haplotype (QQ) have higher (P = 0.0096) TNB_EBV than heterozygous carriers (qq).

Figure 1

Click here to download Figure Figure 1.jpg

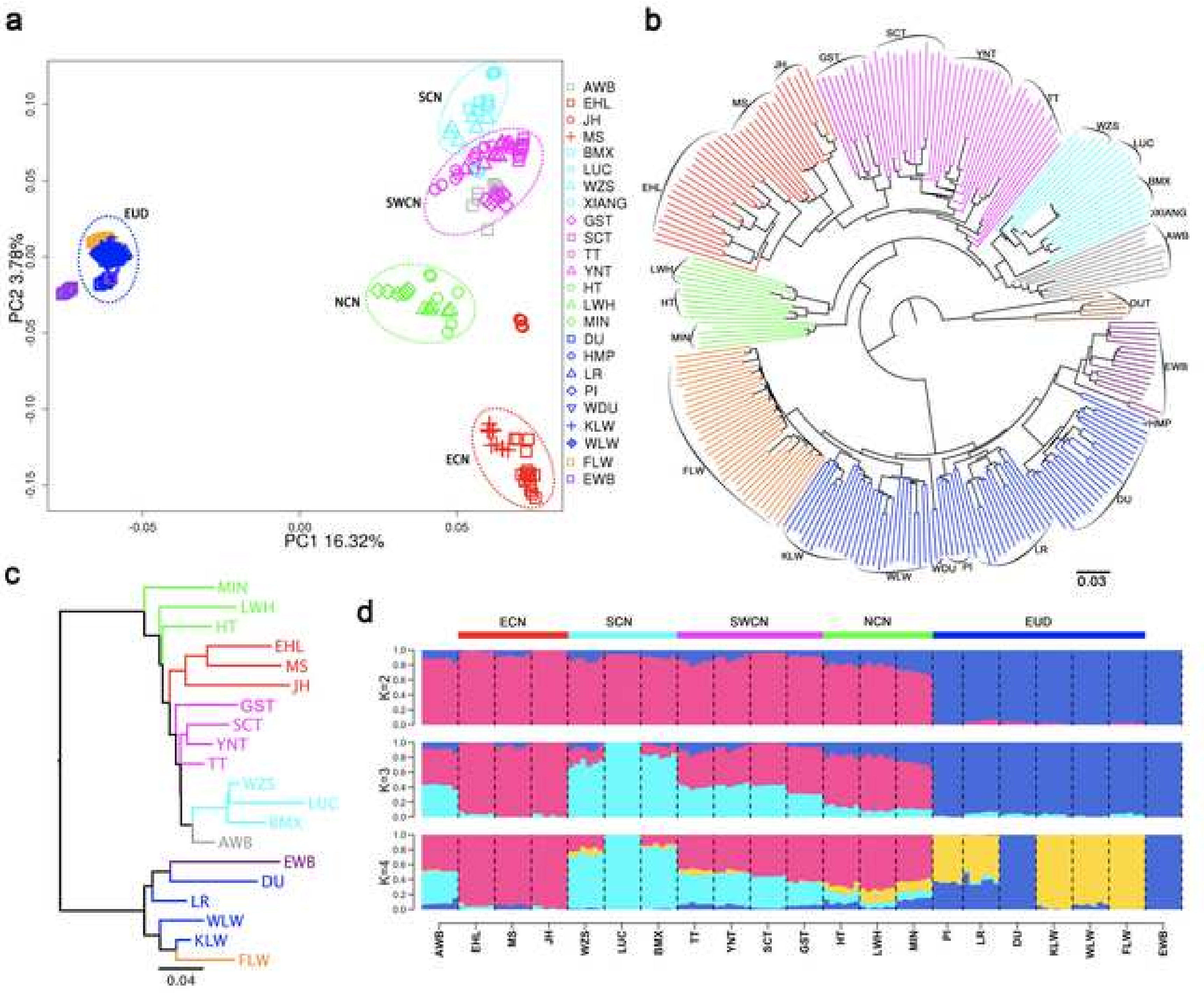

Figure 2

Click here to download Figure Figure 2.jpg

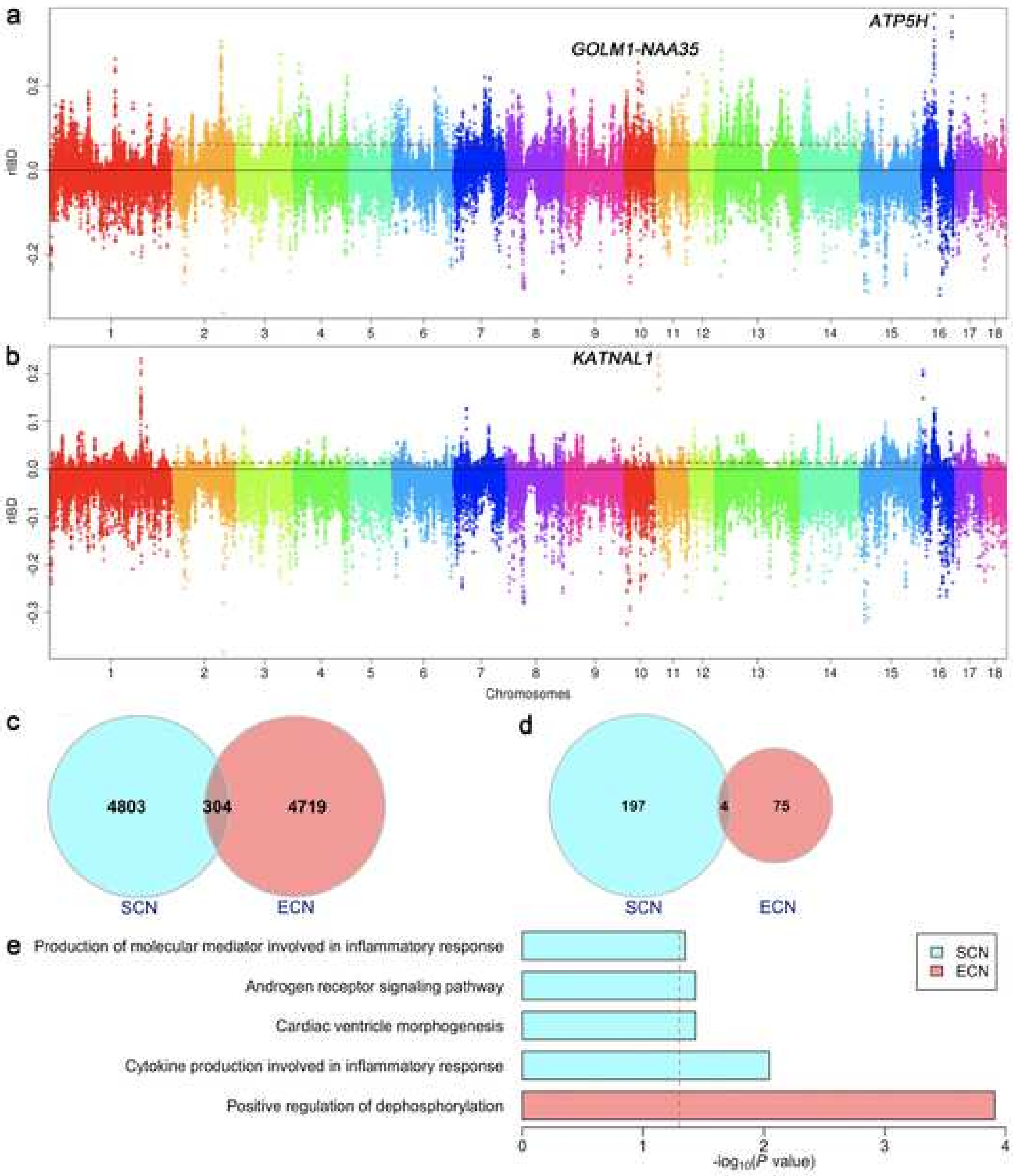

Figure 3

Click here to download Figure Figure 3.jpg

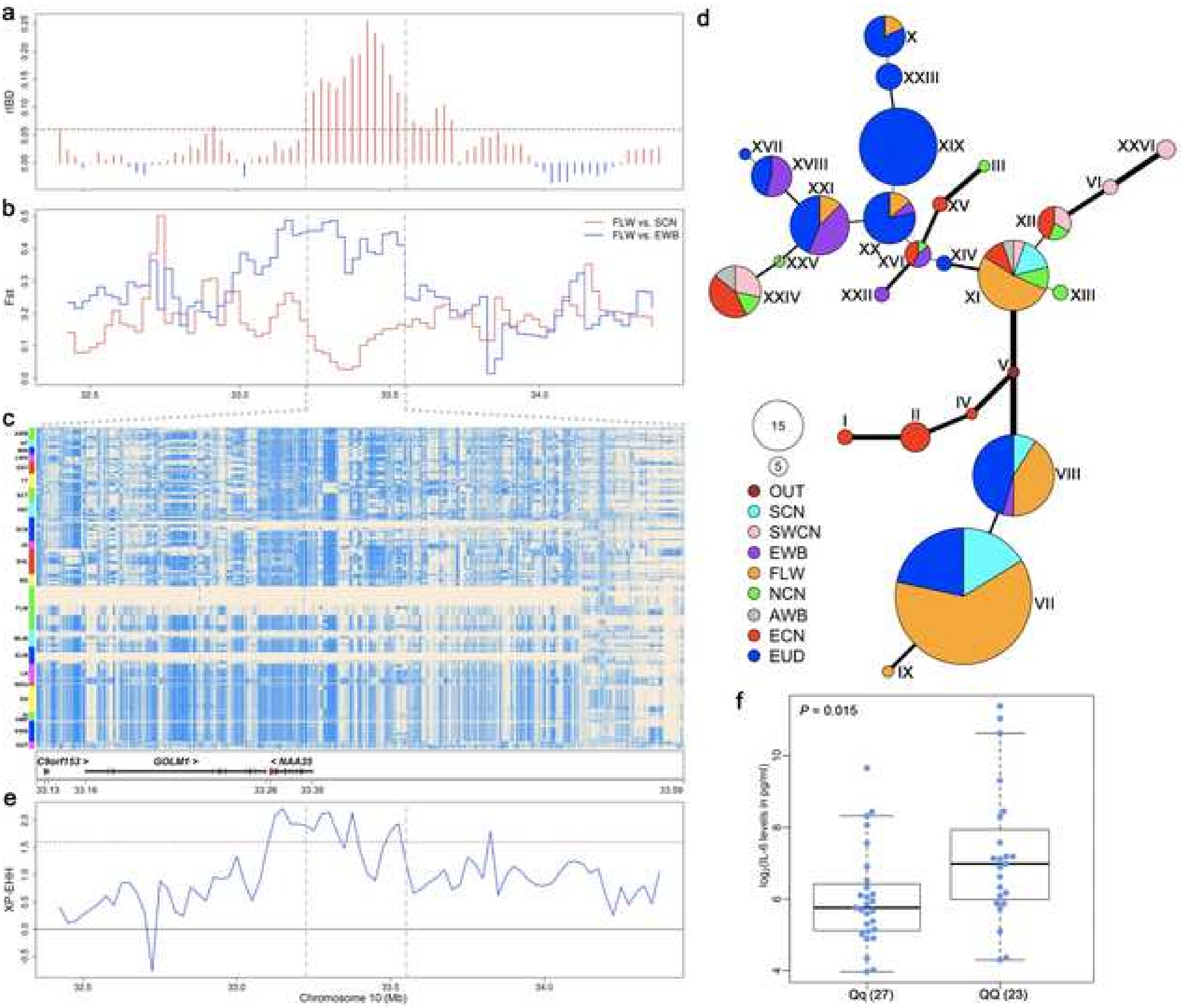

Figure 4

Click here to download Figure Figure 4.jpg

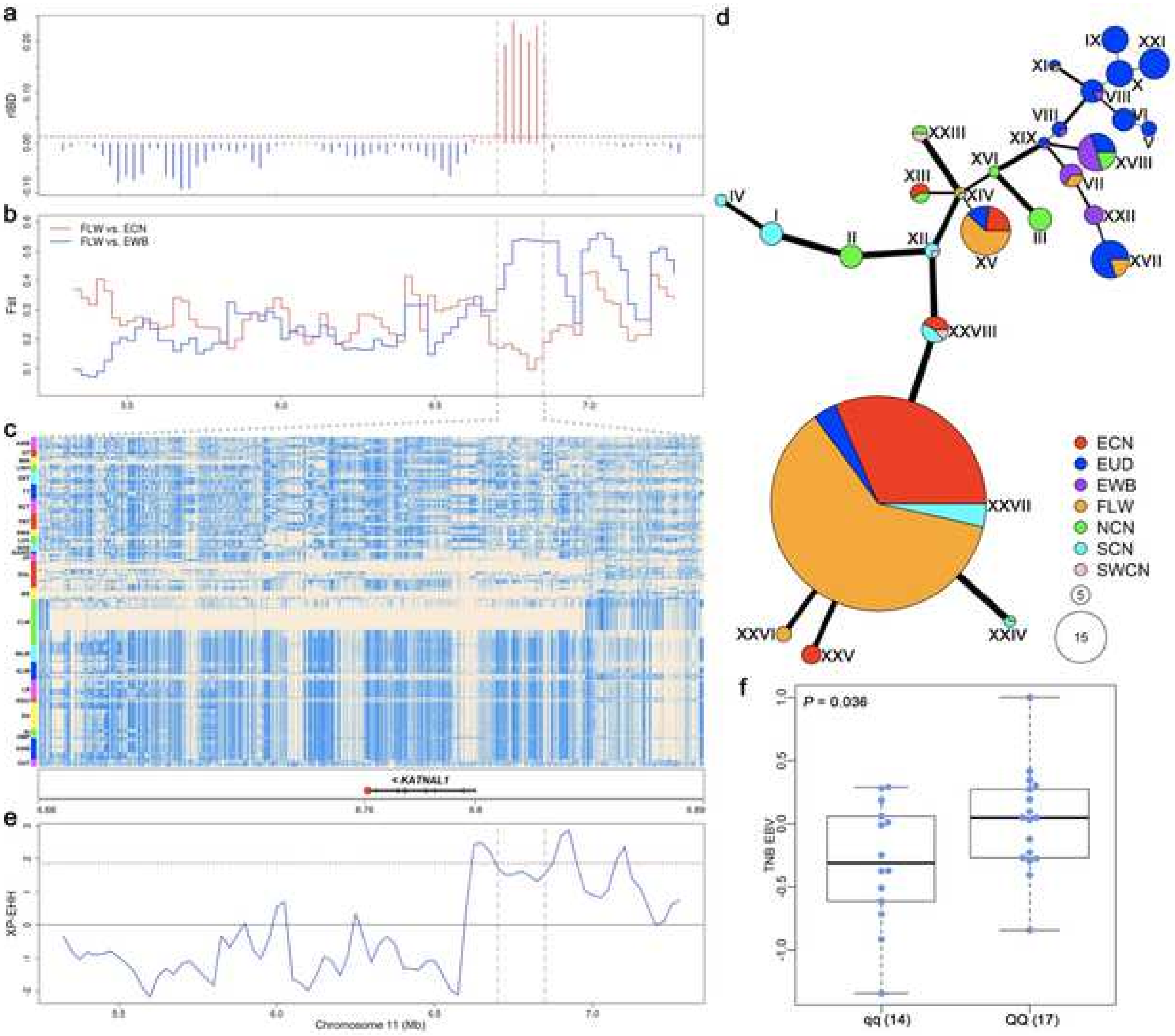

Figure 5

Click here to download Figure Figure 5.jpg

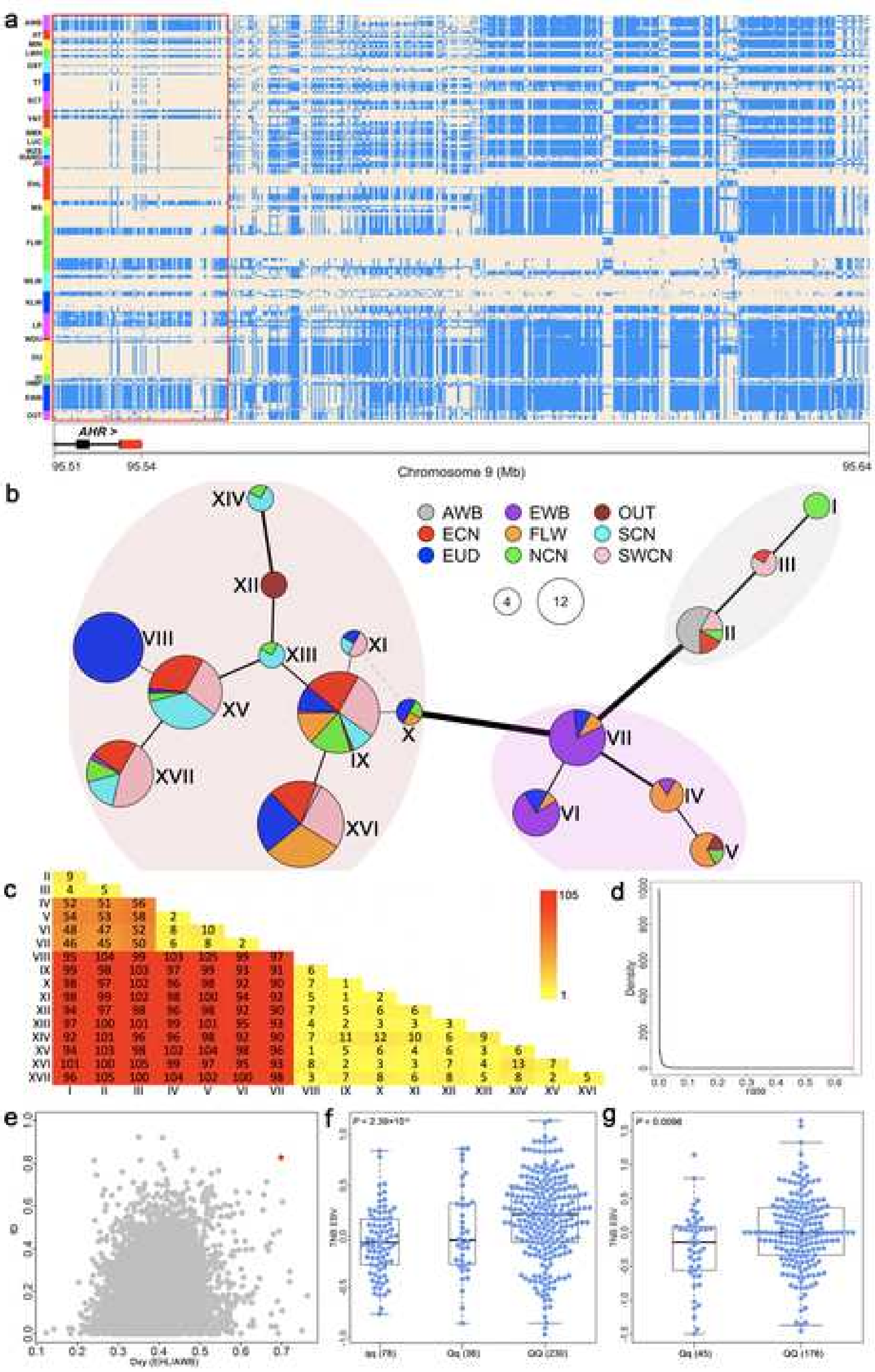

Click here to access/download
Supplementary Material
Supplementary Information.docx

Supplementary Table S6

Click here to access/download
Supplementary Material
Supplementary Table 6.xlsx

Supplementary Table S7

Click here to access/download
Supplementary Material
Supplementary Table 7.xlsx
