# Supplementary material for: Introgression of Eastern Chinese and Southern Chinese haplotypes contributes to the improvement of fertility and immunity in European modern pigs
Source: Gigascience. 2020 Mar 6;9(3):giaa014. doi: 10.1093/gigascience/giaa014 (PMC7059266; doi:10.1093/gigascience/giaa014)
Supplement: giaa014_GIGA-D-19-00160_Revision_1 [file giaa014_giga-d-19-00160_revision_1.pdf]

GigaScience

Introgression of Eastern Chinese and Southern Chinese haplotypes contributes to the improvement of fertility and immunity in European modern pigs

--Manuscript Draft--

**Manuscript Number:**	GIGA-D-19-00160R1
**Full Title:**	Introgression of Eastern Chinese and Southern Chinese haplotypes contributes to the improvement of fertility and immunity in European modern pigs
**Article Type:**	Research
**Funding Information:**	National Natural Science Foundation of China (31525023) — Prof. Jun Ren<br>National Key Research Project of China (2016ZX08006-5) — Prof. Jun Ren
**Abstract:**	Pigs were domesticated independently from European and Asian wild boars nearly 10,000 years ago. Chinese indigenous pigs have been historically introduced to improve Europe local pigs. However, the geographic origin and biological functions of introgressed Chinese genes in modern European pig breeds remain largely unknown. Here we explored whole-genome sequencing data from 266 Eurasian wild boars and domestic pigs to produce a fine-scale map of introgression between French Large White (FLW) and Chinese pigs. We show that FLW pigs had historical admixture with both Southern Chinese (SCN) and Eastern Chinese (ECN) pigs approximately 200–300 years ago. Moreover, a set of SCN haplotypes was shown to be beneficial for improving disease resistance and those with ECN haplotypes are favorable for improved reproductive performance in FLW pigs. Intriguingly, we found both human-mediated and archaic introgression events at the AHR locus, at which the archaic haplotype contributes to increased fertility in both ECN and FLW pigs. This study advances our understanding of the breeding history of global domestic pigs and highlights the importance of artificial hybridization and natural archaic introgression in the formation of phenotypic characteristics in domestic animals.
**Corresponding Author:**	Jun Ren<br><br>CHINA
**Corresponding Author Secondary Information:**	
**Corresponding Author's Institution:**	
**Corresponding Author's Secondary Institution:**	
**First Author:**	Hao Chen
**First Author Secondary Information:**	
**Order of Authors:**	Hao Chen<br>Min Huang<br>Bin Yang<br>Zhongping Wu<br>Zheng Deng<br>Yong Hou<br>Jun Ren<br>Lusheng Huang
**Order of Authors Secondary Information:**	
**Response to Reviewers:**	Louis Bernatchez

Powered by Editorial Manager® and ProduXion Manager® from Aries Systems Corporation

Associate Editor, GigaScience
Oct 17, 2019
Dear Dr. Louis Bernatchez,

We are grateful to your and the reviewers' comments on our manuscript entitled "Population genomic data reveal origin and phenotypic effect of Chinese haplotypes introgressed into European modern pigs" (Manuscript ID: GIGA-D-19-00160). We greatly appreciate the opportunity to further revise this manuscript.

According to your suggestions, our article was polished by a professional editing company and we believe that the readability of the polished article has been greatly improved. In addition, we revised this paper on the basis of the two reviewers' comments. For details, please see our point-to-point response to these comments. All revisions are indicated in red in the manuscript. We have collected Authors' ORCIDs, including Hao Chen (0000-0002-5210-8924), Min Huang (0000-0001-9933-012X), Bin Yang (0000-0002-0689-0628), Zhongping Wu (0000-0001-5246-5190), Yong Hou (0000-0002-1351-9288) and Jun Ren (0000-0001-6664-3998). We did not develop new software and thus did not have RRIDs. According to your suggestion, we presented the 50-kb regions of introgression in supplementary tables 2 and 3.

We sincerely hope that the revised manuscript would satisfy you and the two reviewers and can be accepted for publication. Please do not hesitate to contact me if you have any other questions or comments.

Best regards,

Jun Ren
Reviewer #1
The paper describes interesting analyses on the origin and extend of Asian introgression into European commercial pig breeds. The amount of work is impressive and significantly contributes to our understanding of past introgression and persistence of haplotypes in European breeds. However, I had vaious questions about the methodology that I could not find the answer to in the paper. I also have some other issues that I think should be addressed before the manuscript can be considered for publication.
Response: Thank you for your positive comments. We have revised this paper on the basis of your comments.

The use of the English language is not always appropriate throughout the paper. Although it does not influence the overall readability and clarity of the message, I do think the manuscript will benefit from careful editing.
Response: According to this suggestion, our manuscript was polished by a professional editing company, LetPub (www.letpub.com).

The level of detail about the methodology is very inconsistent. Some analyses are described in much detail, whereas other analyses lack detail and cannot be reproduced. I think it is crucial to read through the methods again and add all details that will enable the readers to repeat the analysis. For example, details about how the haplotypes were constructed in the various haplotype plots using the pegas package should be provided. How many SNPs were used with which filtering, how was the phasing done, the distances calculated, etc.. Also, the results from the ALDER analyses should be shown somewhere (perhaps in the supplements?) because now it is too vague how the inference of 200-300 years was obtained.
Response: Thank you for your suggestion. We presented a more detailed description of the methodology in the revised manuscript. We also submitted all essential codes and files of the methodology to the GigaDB temporary ftp site (ftp://user14@parrot.genomics.cn, username = user14, password = JunCNPig). For details, please see the readme text file. We provided details of the methods for haplotype reconstruction and estimation of admixture time. For your convenience, we show the relevant sentences as below:
"We also constructed haplotype networks using SNPs with a MAF of greater than 0.05 and call rates of greater than 90% at the GOLM1-NAA3 (298 SNPs), KATNAL1 (529 SNPs) and AHR (68 SNPs) loci. We explored the fastPhase function with 1000 iterations in Beagle v4.0 [39] to phase haplotypes and used the "haploNet" command

in the R package "pegas" [40] to calculate the pairwise differences between haplotypes. We selected SNPs with an MAF of greater than 0.05, a call rate of greater than 90% and an LD (r2) < 0.3 using PLINK v1.9 [27], and then explored the selected SNPs to estimate the admixture time between populations via ALDER v1.0.3 under default parameters [13]. In short, we used the "convert" function in EIGENSTRAT [41] to convert the data format. We set FLW as a mixed population, EWB and SCN as one reference population, EWB and ECN as another reference population, and five years as one generation."

The reference genome assembly 10.2 was used, however the updated assembly 11.1 was produced in January 2017 already (manuscripthttps://www.biorxiv.org/content/10.1101/668921v1.). Especially the GOLM1-NAA35 and the AHR region occurred on multiple contigs and contained a gap nearby in 10.2, whereas 11.1 contains a single contig at that locus. I understand that redoing all the analyses on the new build requires a lot of work, but at some point we should move forward, especially if it improves the results. Perhaps a comparison could be made for specific loci?
Response: To address this concern, we mapped three representative regions including the GOLM1-NAA35, KATNAL1 and AHR loci to the Sscrofa11.1 reference genome assembly, and made a comparison of tag SNPs and interval positions at these loci on the two reference genome assemblies (10.2 vs. 11.1). For example, we present the positions of the three regions in both Sscrofa10.2 and Sscrofa11.1 in the main text. We also show the positions of tag SNPs for reconstructing GOLM1-NAA35 and KATNAL1 haplotypes in both Sscrofa10.2 and Sscrofa11.1 at the footnote of supplementary tables 6, 7, 8 and 9.

The rIBD in figure 2a seems to highly overestimate the proportion of Asia-derived haplotypes in the FLW. This seems impossible, given the admixture plot and PCA in figure 1. What were the settings for the fastIBD analysis? I could not find these in the methods. I assume the statement "We detected 5,107 and 5,024 50-kb regions with signatures of potential introgression from SCN or ECN pigs into FLW pigs, respectively" is therefore not correct, but these numbers rather reflect the bins in the far-right tail.
Response: As mentioned in the Method section, we conducted genome scans for regions of potential introgression using sliding windows of 50 kb with a step size of 25 kb. We calculated rIBD values for each window via IBD fragments that were iterated 10 times. As you assumed, the 5,107 and 5,024 50-kb regions are in the far-right tail of 5% empirical distribution. We defined these regions as the regions of potential introgression from SCN or ECN pigs into FLW pigs. Some of these regions are overlapped, corresponding to 4,803 and 4,719 non-overlapped regions, respectively. These regions are 240 Mb and 236 Mb in length, accounting for 8.9% and 8.7% of the total length of the pig genome (2.7G). The Asian fractions (8.9% and 8.7%) are roughly comparable to those in FLW as revealed by the ADMIXTURE analysis. Please see Figure 1d. Hence, the rIBD in figure 2a did not highly overestimate the proportion of Asia-derived haplotypes in the FLW.

Line 282: "with a strong introgression signal at the AHR locus (SSC9: 92.25-97.45 Mb)". I don't think this is a strong introgression signal, you have some peaks that stand out much stronger. What made you decide to focus on this locus?
Response: Boss et al. reported that the AHR region was one of the longest regions of introgression from Chinese pigs to Large White pigs, and an Asian-derived missense mutation in the AHR gene was significantly associated with litter size in Large White pigs (Nature Communication, 2014, doi: 10.1038/ncomms5392). Hence, we focused on this locus by using it as a proof of concept. We confirmed the significant association at this locus and then made a close examination on it, which enabled us to clarify the origin of the favorable haplotype for fertility.

Looking at the clustering, the sampled wild boar clearly represents the SCN lineage rather than ECN. Without this wild background leading to the ECN domestic lineage, it is impossible to assign the AHR haplotype to an external lineage such as the other Sus species.
Response: The clustering patterns indicate that the sampled wild boars (n = 10) have a closer genetic relationship with SCN pigs as compared to ECN pigs. Nevertheless, we did not think that these wild boars represent the SCN lineage. The ADMIXTURE

Powered by Editorial Manager® and ProduXion Manager® from Aries Systems Corporation

analysis show that ECN and SCN pigs represent two ancient lineages of Chinese indigenous pigs when K = 3 to 4. The wild boars show a mixture of the two ancient lineages. Please see Figure 1d. In fact, two and four of the 10 sequenced wild boars were sampled from Zhejiang province in Eastern China and Jiangxi province in Central China, respectively.

The clustering of one Chinese wild boar haplotype within the French Large white AHR haplotype group also suggests the occurrence of this haplotype in wild boar - although perhaps at low frequency.
Response: It is known that domestic pigs had recurrent hybridization with wild boars after domestication (Nature Genetics, 2015, doi: 0.1038/ng.3394). Given the very low frequency (1/20) of this haplotype in wild boars, we believe that the occurrence of this haplotype in wild boar is most likely due to gene flow between domestic pigs and wild boars.

You filtered out SNPs in 266 individuals with a MAF less than 0.01 -> this will remove many OUT-specific alleles. What would happen to the haplotype distances if you do include species-specific alleles, and how does that influence your conclusions?
Response: We agree with your opinion that a proportion of OUT-specific alleles will be removed under the filtering criteria of MAF less than 0.01. On the other side, this filtering criteria has been widely adopted to reduce the risk of sequencing errors especially when a limited number of samples were analyzed (Nature Genetics. doi:10.1038/s41588-018-0250-5 for example). We assume that haplotype distances between OUT and domestic pigs will be increased if we include species-specific alleles. When we analyzed the KATNAL1 locus, we did not include OUT haplotypes due to their low frequencies (Figure 4). To avoid potential bias, we removed OUT haplotypes at the GOLM1-NAA35 locus and present a new version of Figure 3 and Figure S6. From the two new figures, you can see that the deletion of the OUT haplotypes did not affect our result. At the AHR locus, the most frequent haplotype (XVI) appeared 100 times in all 266 sequenced individuals, including 30 FLW pigs, 24 other LW pigs, 18 EHL pigs and 26 in Tibetan pigs. This haplotype together with the OUT haplotype XII formed a major haplotype group, which was divergent from another two major haplotype groups from Eurasian wild boars and domestic pigs (fig. 5b). This pattern apparently conforms to the archaic introgression assumption. Altogether, our conclusion is not affected by filtering out SNPs with MAF of less than 0.01 in 266 individuals.

Regions of lower recombination can create outlier signals because of drift, this potential bias should be discussed
Response: We agree with this comment. Low recombination and genetic drift are two factors that cause reduced genetic variability at target regions, i.e. potential bias of selection signature.

The discussion section could better be read as some sort of conclusion, because lots of discussion is already provided in the results section (for example, line 211-221). I do like this structure (as in the discussion by section rather than discussion of all findings at the end) but it should be stated in the headers.
Response: Thank you for your suggestion. We added two headers for the two subsections of Discussion. One is entitled “Introgression of both SCN and ECN pig DNA contributed to the genetic improvement of European modern pig breeds” and another is entitled “Both naturally occurring interspecies hybridization and human-driven crossbreeding played important roles in the development of global pig breeds”.

Why is the highest peak, ATP5H, indicated in figure 2, but not discussed??
Response: As shown in Figure 2, the ATP5H region displayed the strongest signature of introgression from SCN pigs into FLW pigs. However, we did not detect significant selection signals in this region, neither between FLW and European wild boars (Figure 1a) nor between FLW and Large White pigs from other countries (Figure 1b) via the XP-EHH approach. This indicates that the ATP5H region did not experience directional selection after the introgression event and thus did not contribute to the selective breeding of FLW pigs. For this reason, we did not discuss about the ATP5H region.

Figure 1. Selection signals in the ATP5H region unraveled by the XP-EHH analysis. (a) Signals between FLW and European wild boars. (b) Signals between FLW and Large

Powered by Editorial Manager® and ProduXion Manager® from Aries Systems Corporation

White pigs from other countries. The red dashed line indicates the 5% threshold line. The pink shaded area represents the ATP5H region.

Different coloration in figS12a and figS12b is confusing (the European wild).
Response: Corrected.

Why does one EWB cluster with the EUD in fig 1a?
Response: In fact, two European wild boars cluster with the EUD in an overlapped way in the PC1 and PC2 plots (Fig. 1a). The two wild boars were sampled from the Samos island of Greek. We assume that they had a considerable proportion of genetic components of the EUD due to gene flow as mentioned above. We further conducted PC3 and PC4 analyses for all sampled pigs. In the PC3 and PC4 plots, the two individuals cluster with other European wild boars. For details, please see the below figure. In addition, the two individuals together with the other European wild boars define a branch separating from the EUD in the NJ tree (Fig 1b).

Figure 2. Principal component analysis (PCA) of Chinese and European pigs.

Was the t-test the most appropriate test for the haplotype association analysis? What about potential family relationships that could bias the outcome?
Response: We used EBV values of TNB as phenotypic traits for the association test at the KATNAL1 and AHR loci. The EBV values were calculated using the DMU software that treated family relationships, estrus, year, season, parity and pregnancy duration as fixed effects in the statistical model. For the association test at the GOLM1-NAA35 locus, we collected blood samples from unrelated Tibetan pigs that did not have common ancestors within three generations. Hence, we believe that potential family relationships did not bias the outcome.

How were the tagged SNPs for the haplotype association analyses selected?
Response: We explored the Beagle software to phase haplotypes at target loci and selected the least number of SNPs as tagged SNPs that can distinguish all haplotypes at the target loci through their genotypes. For instance, all KATNAL1 haplotypes in FLW pigs can be inferred using the genotypes of the eight tagged SNPs that we selected. For details, please see the figure shown below.

Figure 3. The eight tagged SNPs for phasing KATNAL1 haplotypes in FLW pigs.

Reviewer #2:
Chen et al. in their manuscript "Population genomic data reveal origin and phenotypic effect of Chinese haplotypes introgressed into European modern pigs" performed a thorough analysis explaining the introgression history of Chinese pigs from two distinc locations - Southern (SCN) and East (ECN) China - into an European breed, French Large White (FLW). The authors unravel introgression signatures from ECN and SCN and link them to immunity and reproductive traits respectively. They expand in two examples, the GOLM1-NAAR35 haplotype and the KATNAL1. Finally, they shed light in to the AHR intogressed haplotype introgressed form ECN pigs into Large-White pigs however showing that the AHR haplotype derived from ancient pig species. I consider this a very well written article of clear relevance to the field.
Response: We greatly appreciate your positive comments on our manuscript.

The authors mention Bosse et al. Nature Communications 2014 analysis as the pipeline used in their manuscript. In Bosse the authors use an independent method to verify gene flow between pig populations, namely D-statistics. Do the authors think that D-statistics in here would provide more robustness to their analysis?
Response: Yes, we agree that D-statistics would provide more robustness to our analysis. Bosse et al. also explored the rIBD approach to detect genomic signatures of introgression. We adopted this approach to uncover the introgression signals across the genome in this study. We further used complementary analyses including Fst, haplotype network and heatmap to obtain additional evidence for the introgression conclusion at the GOLM1-NAAR35, KATNAL1 and AHR loci

Page 6 Line 120: ECN pigs represented by Jinhua pigs and SCN pigs represented by Luchuan pigs appeared as the two ancestral lineages of Chinese pigs when K=3 (fig. 1d). Please consider adding … Jinghua pigs (JH) …

	Response: Corrected.<br><br>Page 6 Line 129: We detected 5107 and 5024 50-Kb regions with signatures of potential introgression from SCN or ENC pigs into FLW pigs.To help follow-up studies it would be convenient to report an excel table with the set of detected regions with potential signatures of introgression, and not only the seven with "strong singatures of introgression" (supplementary Table 2).<br>Response: According to your suggestion, we present two supplementary tables that provide details for the 5170 (supplementary Table 2) and 5024 (supplementary Table 3) 50-kb regions with signatures of potential introgression from SCN or ENC pigs into FLW pigs.<br><br>Page 30 Line 704: Fig1. Population relationship and structure. (a) Neighbour-joining (NJ) tree based on an identity-by-state matrix among individuals. (b) Principal component analysis (PCA) of Chinese and European pigs. The figure shows in panel (a) the PCA analysis and in panel (b) the NJ tree.<br>Response: Thank you for your reminding. We have corrected this error.<br><br>Supplementary Figure 3. The colored dashed in the phylogenetic tree represent different genetic groups. Some clarifications or legends on that would be much appreciated, SCN, ECN, NCN…<br>Response: According to this comment, we have added clarifications for SCN, ECN, SWCN, NCN and EUD in the new version of Supplementary Figure 3.<br><br>Page 7 Line 36. SCN pigs and ECN pigs were enriched in the immune-related signalling and fertility pathways, respectively (fig. 2e). ECN genes are related to "positive regulation of dephosphorilation", is that related to fertility?<br>Response: Yes.<br><br>Page 15 Line 335. Better clarification on FLW pigs versus OUT animals were only 1.51 and 1.41, respectively (supplementary fig S13).<br>Response: Done. Please see the new version of Supplementary Figure 13<br><br>Supplementary Figure 12 is not mentioned in the text.<br>Response: Added.
**Additional Information:**	
**Question**	**Response**
Are you submitting this manuscript to a special series or article collection?	No
**Experimental design and statistics**<br><br>Full details of the experimental design and statistical methods used should be given in the Methods section, as detailed in our Minimum Standards Reporting Checklist. Information essential to interpreting the data presented should be made available in the figure legends.<br><br>Have you included all the information requested in your manuscript?	Yes
**Resources**	Yes

Powered by Editorial Manager® and ProduXion Manager® from Aries Systems Corporation

A description of all resources used, including antibodies, cell lines, animals and software tools, with enough information to allow them to be uniquely identified, should be included in the Methods section. Authors are strongly encouraged to cite Research Resource Identifiers (RRIDs) for antibodies, model organisms and tools, where possible.<br><br>Have you included the information requested as detailed in our Minimum Standards Reporting Checklist?	
**Availability of data and materials**<br><br>All datasets and code on which the conclusions of the paper rely must be either included in your submission or deposited in publicly available repositories (where available and ethically appropriate), referencing such data using a unique identifier in the references and in the “Availability of Data and Materials” section of your manuscript.<br><br>Have you have met the above requirement as detailed in our Minimum Standards Reporting Checklist?	No
If not, please give reasons for any omissions below.<br><br>as follow-up to "**Availability of data and materials**<br><br>All datasets and code on which the conclusions of the paper rely must be either included in your submission or deposited in publicly available repositories (where available and ethically appropriate), referencing such data using a unique identifier in the references and in the “Availability of Data and Materials” section of your manuscript.	All sequence data will be submitted to a publicly available repositories when this manuscript is acceptable for publication in GigaScience.

Powered by Editorial Manager® and ProduXion Manager® from Aries Systems Corporation

Have you have met the above requirement as detailed in our Minimum Standards Reporting Checklist?<br><br>"	

Powered by Editorial Manager® and ProduXion Manager® from Aries Systems Corporation

Manuscript

Click here to access/download;Manuscript;Introgressed Chinese haplotypes in French Large White_8.docx

Click here to view linked References

Introgression of Eastern Chinese and Southern Chinese haplotypes contributes to the improvement of fertility and immunity in European modern pigs

Hao Chen[§], Min Huang[§], Bin Yang, Zhongping Wu, Zheng Deng, Yong Hou, Jun Ren[*†], Lusheng Huang[*]

State Key Laboratory of Pig Genetic Improvement and Production Technology, Jiangxi Agricultural University, Nanchang, P.R. China.

[§] Both authors contributed equally to this study and should be considered co-first authors

[†]Current address: College of Animal Science, South China Agricultural University, 510642, Guangzhou, P.R. China

Correspondence author: Jun Ren, Lusheng Huang

State Key Laboratory of Pig Genetic Improvement and Production Technology

Jiangxi Agricultural University

Nanchang 330045, P. R. China.

Phone: 0086-791-83805967; Fax: 0086-791-83900189;

E-mail: renjunjxau@hotmail.com; lushenghuang@hotmail.com

Abstract

Pigs were domesticated independently from European and Asian wild boars nearly 10,000 years ago. Chinese indigenous pigs have been historically introduced to improve Europe local pigs. However, the geographic origin and biological functions of introgressed Chinese genes in modern European pig breeds remain largely unknown. Here we explored whole-genome sequencing data from 266 Eurasian wild boars and domestic pigs to produce a fine-scale map of introgression between French Large White (FLW) and Chinese pigs. We show that FLW pigs had historical admixture with both Southern Chinese (SCN) and Eastern Chinese (ECN) pigs approximately 200–300 years ago. Moreover, a set of SCN haplotypes was shown to be beneficial for improving disease resistance and those with ECN haplotypes are favorable for improved reproductive performance in FLW pigs. Intriguingly, we found both human-mediated and archaic introgression events at the *AHR* locus, at which the archaic haplotype contributes to increased fertility in both ECN and FLW pigs. This study advances our understanding of the breeding history of global domestic pigs and highlights the importance of artificial hybridization and natural archaic introgression in the formation of phenotypic characteristics in domestic animals.

Introduction

Integrated genomic and archaeological evidence have illuminated the fact that the wild boar (*Sus scrofa*) originated in the Islands of Southeast Asia about 5 million years ago and then dispersed throughout Eurasia. Approximately 1 million years ago, geographic isolation caused by glacial events hampered the continuous gene flow among Eurasian wild boars, causing European and Asian wild boars to differentiate from each other [1-4]. About 10,000 years ago, European and Asian wild boars were domesticated independently in the Near East and China, respectively [3, 5, 6]. After long-term artificial selection and natural selection, abundant genetic resources of domestic pigs appeared in China, accounting for about one-third of global breeds [7, 8]. Chinese pigs are distributed in diverse geographic regions and have different breed features. For example, Erhualian (EHL) and Meishan pigs in East China are known for their prolificacy, with a litter size of more than 15, and for their thick skin. Luchuan (LUC) and Bama pigs in South China have inferior reproductive performance (8–10 piglets per parity), have thin skin and excellent heat resistance [7]. These pig breeds not only play a critical role in the Chinese pig industry, but also have contributed to the development of international commercial breeds, such as the Large White (LW) [9, 10].

Chinese pigs were introduced to Europe mainly during three historical periods [7]. From 1685 to 1757, the Qing Dynasty set up four foreign trade ports: two in East China (Shanghai and Ningbo) and two (Zhangzhou and Guangzhou) in South China. Europe (especially England) had frequent trade with China through these four ports, mainly via the East India Company. This raises the possibility that Eastern Chinese (ECN) and Southern Chinese (SCN) pigs may have been transported to European countries during this period. From 1757 to 1841, only the Guangzhou port in South China was permitted access to foreign trade, and a ban was imposed on maritime trade or intercourse with foreign countries in 1757. It is well documented that SCN pigs had been introduced to England for the hybridization of local pigs during this period,

contributing to the formation of Berkshire [9] and LW pigs [10]. In 1978, the Chinese government launched the reform and open-door policy. Since then, ECN pigs, including Meishan, Jinhua, and Jiaxing Black, have been introduced into France, America, and Japan for the development of prolific synthetic lines [7].

Recently, whole-genome re-sequencing analysis has confirmed the human-mediated translocation of Chinese pigs into Europe that provided genetic variations for the selective breeding of modern commercial LW pigs [11]. However, it remains unknown if SCN or ECN pigs or both were introduced to Europe, because previous studies used a limited number of Chinese pigs from different locations as a whole population. French Large White (FLW) pigs are known for their excellent reproductive performance. A remarkable genetic improvement of litter size has been witnessed in FLW pigs over the past decades, but the molecular mechanisms underlying the fecundity remain unclear, although the fecundity is speculated to be related to the recent introgression of highly prolific Chinese pigs such as ECN pigs [7]. Further studies are required to test this speculation.

In this study, we explored whole-genome sequencing data of 266 Eurasian pigs to show that both SCN and ECN haplotypes were introgressed into LW pigs ~200–300 years ago. Some of the introgressed haplotypes have been under preferential selection to improve fertility and immunity in FLW pigs. Interestingly, the prolificacy-associated *AHR* haplotype was likely introgressed from an archaic *Sus* population into ECN pigs via interspecies hybridization and was then introduced from ECN pigs to FLW pigs through human-driven transportation. These findings advance our understanding of the breeding history and genetic mechanisms underlying breed characteristics of global domestic pigs. Moreover, this study highlights the importance of artificial intraspecies crossbreeding and natural interspecies hybridization on the phenotypic characteristics of domestic animals.

Results

Whole-genome sequencing data

We obtained whole-genome sequencing data of 266 animals from 25 populations (**supplementary table S1**), including 36 highly prolific FLW pigs from the nucleus populations of two breeding companies. The 36 pigs were selected due to their total number born (TNB) piglets of more than 19 and distant genetic relationship between each individual (**supplementary fig. S1**). High-depth re-sequencing was conducted on a Hiseq 2000 or 2500 sequencer (Illumina, USA). After filtering raw data (see Methods), we called 32.7 million single nucleotide polymorphisms (SNPs) from the 266 individuals. For the 28 LW pigs whose sequence data were retrieved from the public NCBI database (see Methods), we used the Illumina Porcine SNP60 chip [12] data set to identify their origin. We demonstrated that 14 individuals belonged to the American Large White (ALW) lineage, and the other 14 individuals belonged to the Dutch Large White (DLW) line (**supplementary fig. S2**).

Genetic differentiation between SCN and ECN pigs

Eurasian wild boars began to differentiate as early as ~1 million years ago [2, 3], and Chinese and European wild boars were independently domesticated about 10,000 years ago [1, 3]. The remarkable genetic differentiation between Chinese and Western pigs was reflected in the results from principal component analysis (PCA), phylogenetic analysis and admixture analysis (**fig. 1**). In our PCA analysis, the first principal component (PC1) accounted for 16.32% of the total eigenvalue, which clearly separated the Chinese pig from the Western pig. The second principal component (PC2) showed the differentiation among Chinese pigs, especially between SCN and ECN pigs (PC2 = 3.78%, **fig. 1a**). In a neighbor joining tree between individuals (**fig. 1b**) and populations (**fig. 1c**), Chinese and Western pigs defined two separate clades. For Chinese domestic pigs, SCN and ECN pigs formed two different branches. The

clustering pattern was similar to the maximum likelihood tree revealed with TreeMix analysis, in which two Sumatras wild boars, one *Sus barbatus*, one *Sus verrucosus*, one *Sus cebifrons*, one *Sus celebensis*, and one *Phacochoerus africanus* were treated as an outgroup (OUT), and the interpretation of the maximum likelihood tree reached 99.9% (**supplementary fig. S3**). In an admixture analysis, Chinese pigs and European pigs showed two distinct ancestral lineages when K = 2, although there were gene flows between the two groups, especially the North Chinese pig, that clearly mixed with European pig lineages, whereas LW (including FLW) pigs showed signatures of admixture with Chinese pigs. ECN pigs represented by Jinhua (JH) pigs and SCN pigs represented by Luchuan pigs appeared as the two ancestral lineages of Chinese pigs when K = 3 (**fig. 1d**). Altogether, these findings not only confirmed the independent domestication of Chinese and European pigs, but also revealed that SCN pigs and ECN pigs have marked genetic differentiation and represent two ancient lineages of the Chinese domestic pig.

SCN and ECN pigs were introgressed into Europe between 220 and 310 years ago

To determine whether SCN and ECN pigs were introduced into Europe via human-mediated transportation, we performed relative identity-by-descent (rIBD) analysis using whole genome sequencing data (see Methods). We detected 5,107 and 5,024 50-kb regions with signatures of potential introgression from SCN (**supplementary table S2**) or ECN (**supplementary table S3**) pigs into FLW pigs, respectively (**figs. 2a and 2b, supplementary fig. S4**). The introgressed DNA from SCN and ECN pigs differed greatly in FLW pigs, with an overlap of only 6.0% introgression regions (**fig. 2c**) and 2.9% genes within these regions (**fig. 2d**). We thus performed Gene Ontology (GO) and Kyoto Encyclopedia of Genes and Genomes (KEGG) pathway enrichment analysis on the genes located in the introgressed regions. The genes within the regions of inferred introgression with SCN pigs and ECN pigs were enriched in the

immune-related signaling and fertility pathways, respectively (**fig. 2e**). We further used ALDER software [13] to estimate the time of admixture between FLW and SCN or ECN pigs, which yielded an estimate of 53 ± 9 (265 ± 45 years) and 54 ± 9 (270 ± 45 years) generations ago, respectively. This estimation was consistent with historical records stating that SCN pigs were deliberately transported to England at the onset of the first Industrial Revolution and contributed to the breeding of LW pigs [11]. In addition, these results supported our hypothesis that ECN pigs were also introduced into Europe to improve the productivity of local pigs between 1685 and 1757.

The introgressed *GOLM1-NAA35* haplotype from SCN pigs has been under selection to enhance the disease resistance of FLW pigs

We detected seven genomic regions with strong signatures of introgression from SCN pigs in the genomes of FLW pigs (rIBD value > 0.2; **supplementary table S4**). Two adjacent genes (3,511 bp apart), *GOLM1* and *NAA35*, were located in one of these seven regions. The *GOLM1* gene encodes a type II Golgi transmembrane protein, which is mainly synthesized in the rough endoplasmic reticulum, assists in processing proteins in the Golgi and is responsive to viral infections [14]. In 2016, Li *et al*. [15] reported that the *GOLM1-NAA35* locus markedly modulated the cytokine interleukin-6 (IL-6) production by human immune cells in response to multiple pathogens. Given the important role of the *GOLM1-NAA35* locus in disease resistance, we chose this locus for further study.

We first made a close examination of the rIBD results for a 2-Mb region encompassing the *GOLM1-NAA35* locus (SSC10: 33.20 – 33.58 Mb in Sscrofa10.2 and 29.15 - 29.50 Mb in Sscrofa11.1). We found that the frequency of shared IBD haplotypes between FLW and SCN pigs at the *GOLM1-NAA35* locus was significantly higher than those in the surrounding regions (**fig. 3a**). Moreover, we observed remarkably elevated genetic differentiation (F_{ST}) between

FLW pigs and European wild boars, in contrast to the particularly decreased F_{ST} between FLW and SCN pigs in the *GOLM1-NAA35* region we observed (**fig. 3b**). In addition, there were four main *GOLM1-NAA35* haplotypes in FLW pigs. Most individuals (32 out of 36) carried haplotypes similar to those of SCN pig (**fig. 3c**).

Next, we used 3,447 SNPs in the *GOLM1-NAA35* region to construct an NJ tree (**supplementary fig. S5**). We found that most FLW pigs (n = 32) clustered with SCN pigs to form a branch that was separated from ECN pigs and European pigs, whereas only a small number of FLW pigs (n = 4) clustered with European pigs, which was in stark contrast to a genome-wide NJ-tree (**fig. 1a**). We further constructed a haplotype network using 298 SNPs at the *GOLM1-NAA35* locus (**fig. 3d**). We clearly identified haplotype VII as being the main haplotype in FLW pigs, and this haplotype appeared 37 times in all populations, including 23 times in FLW pigs, 8 times in LW pigs, and 6 times in SCN pigs. The SCN-major haplotype VIII and haplotype VII differed at only four different sites, whereas the unique haplotypes (XIX, XXIII and X) of European wild boars and haplotype VII differed at more than 180 sites (**supplementary fig. S6**). These results corroborate the historical introgression of SCN pigs into FLW pigs and illuminate that haplotype VII at the *GOLM1-NAA35* locus in FLW pigs originated from SCN pigs.

We noted that the introgressed haplotype VII was present in other LW pigs at low frequencies but was absent in other European domestic pigs. This was conceivable because all LW populations originated in England where SCN pigs were introduced during the first Industrial Revolution (early 19th century) [7]. Moreover, this introgressed haplotype appeared one time in European wild boars. Considering the outdoor grazing of early European pigs, we believe that European wild boars had admixture with European domestic pigs, after which this haplotype was introgressed from European domestic pigs into European wild boars.

The haplotype heatmap of the *GOLM1-NAA35* region shows that the SCN-originated haplotype VII was frequently present in FLW pigs **(fig. 3c**), which suggested that this haplotype may be selected for in FLW pigs. To verify this hypothesis, we first compared the linkage disequilibrium (LD) values (r^2) of the *GOLM1-NAA35* region and an upstream (3 Mb) region with the same size as the *GOLM1-NAA35* locus. We found that the LD level in the *GOLM1-NAA35* region of the FLW population ($r^2_{0.3}$ = 192.3 kb) was significantly higher than that of all other populations (**supplementary fig. S7a)**, whereas the LD value ($r^2_{0.3}$) in the upstream region was only 17.3 kb, which was similar to most populations **(supplementary fig. S7b**). Subsequently, we performed LD analysis for 10,000 81.9-kb regions randomly sampled across the genomes of 36 FLW pigs (**supplementary fig. S7c**). We found that the LD value (r^2) in the *GOLM1-NAA35* region ranked in the top 2.6% of the 10,000 bootstrap results, which was a significant outlier (P = 0.02) and suggested that the introgressed *GOLM1-NAA35* haplotype likely underwent a preference selection in FLW pigs, resulting in a local increase of LD level in this target region. XP-EHH analysis also showed evidence of selection at the *GOLM1-NAA35* region in FLW pigs but not in other LW pigs (**fig. 3e**).

To examine whether the *GOLM1-NAA35* haplotypes were associated with serum IL-6 content in FLW pigs, we collected venous blood from 54 healthy adult FLW sows at the same physiological stage and determined the IL-6 levels in the serum of each individual using an enzyme-linked immunoassay (ELISA) (**supplementary table S5**). Meanwhile, we defined the *GOLM1-NAA35* haplotypes for each individual using two tag SNPs and then tested the association between these haplotypes and IL-6 content. We found that individuals homozygously carrying the introgressed haplotype (*QQ*) had significantly higher IL-6 concentrations than heterozygotes individuals (*Qq*) (P = 0.015, **fig. 3f**). Altogether, a sensible explanation for the introgression at the *GOLM1-NAA35* locus is that the *GOLM1-NAA35* haplotype was historically introgressed from SCN pigs into LW pigs and then has been under

preferential selection to improve the effective production of IL-6 in response to pathogens and consequently enhance the resistance to infectious disease of FLW pigs.

Historically, South China was renowned as a land of plague with a humid and stuffy environment. It was notorious for local infectious diseases, including malignant malaria that caused high transmission and mortality rates before the Southern Song Dynasty (1127–1279 AD). This hostile environment imposed severe physiological challenges on the inhabitants in South China [7]. Native inhabitants like humans and pigs are believed to have evolved the adaptive mechanisms to address this harsh environment, likely via selection of immune-related genes during the long history of colonization of this area. It is thus conceivable that those genes, including *GOLM1-NAA35* within the introgression regions from SCN pigs, are enriched in immune-related signaling pathway genes. Interestingly, a recent genomic analysis unraveled a list of genes related to immune response under selection in southern Han Chinese, including *G6DP* associated with resistance to malaria [16].

The introgressed *KATNAL1* haplotype from ECN pigs has been preferentially selected to increase the fertility of FLW boars

In FLW pigs, a 200 kb region on chromosome 11 (6.68 - 6.88 Mb in Sscrofa10.2 and 6.92 - 7.12 Mb in Sscrofa11.1) showed the strongest (the highest rIBD value) signal of admixture with ECN pigs, and it contained only one gene, *KATNAL1*. *KATNAL1* regulates microtubule dynamics in testicular support cells, affecting the separation and binding of microtubules. Promoting the rapid reorganization of testicular support cell microtubule arrays is an essential process for spermatogenesis and male fertility [17]. Thus, *KATNAL1* plays an important role in spermatogenesis. Given the top introgression signal at the *KATNAL1* locus and the role of *KATNAL1* in boar fertility, we conducted an in-depth analysis focusing on the *KATNAL1* region using the same method as used for the *GOLM1-NAA35* locus.

We found that the frequency of the shared IBD haplotype between FLW and ECN pigs in the *KATNAL1* region was particularly higher than that in the surrounding segments (**fig. 4a**). There was a remarkable local increase of F_{ST} between FLW pigs and European wild boars and a significant decrease of F_{ST} between FLW pigs and ECN pigs in the *KATNAL1* region **(fig. 4b**). FLW pigs had four main haplotypes in this region. Most individuals (30 out of 36) carried haplotypes highly similar to the ECN haplotypes, and the others were similar to European wild boars and European domestic pigs (**fig. 4c**). Additionally, 30 FLW pigs and ECN pigs were clustered into one large clade while only six FLW pigs were grouped with European pigs in an NJ tree that was constructed with 529 SNPs in the *KATNAL1* gene (**supplementary fig. S8**). Meanwhile, we constructed a haplotype network using these 529 SNPs (**fig. 4d**) and analyzed the nucleotide differences among different haplotypes (**supplementary fig. S9**). The most frequent haplotype (XXVII) appeared 57 times in the 266 tested individuals, including 35 FLW pigs, 18 ECN pigs, 2 ALW pigs and 2 SCN pigs. This haplotype and its closest ECN haplotype (XXV, at five different sites, **supplementary fig. S9**) were divergent from the European pig haplotype groups (**fig. 4d**). These results further demonstrated that the *KATNAL1* haplotypes were introgressed from ECN pigs into FLW pigs.

We performed LD bootstrap sampling and XP-EHH analysis to detect evidence of selection at the *KATNAL1* locus in FLW pigs. First, we compared the LD value (r^2) of the *KATNAL1* region and those of 10,000 randomly selected genomic regions with the same size as the *KATNAL1* gene (43.4 kb). We found that the LD level in the *KATNAL1* region ($r^2_{0.3}$= 437.5 kb) was a significant (P = 0.02) outlier, ranking in the top 2.5% of 10,000 bootstrap results (**supplementary fig. S10**). We also detected a significant selection signal at the *KATNAL1* locus in FLW pigs but not in other LW pigs using XP-EHH (**fig. 4e**). These results suggest that the introgressed *KATNAL1* haplotype from ECN pigs was preferentially selected for in FLW pigs.

Given the important role of *KATNAL1* in male fertility, the fecundity of ECN pigs and historical selection for fecundity in FLW pigs, we speculated that the introgressed *KATNAL1* haplotype could contribute to the improvement of male reproductive performance and thus underwent selection in FLW pigs since introgression. To test this hypothesis, we analyzed the association between the *KATNAL1* haplotypes and FLW boar fertility that was represented by the average estimated breeding value (EBV) for TNB of mating sows. We detected a significant difference in boar fertility between 17 homozygous carriers of the introgressed haplotype (*QQ*) and 14 carriers of non-ECN pig haplotypes (*qq*) (P = 0.036; **fig. 4f**). The EBV for TNB (EBV-TNB) of *QQ* individuals was 0.018, with a difference of 0.32 (which equates to an increase of 0.32 piglets born per parity) compared with *qq* individuals. As TNB is a complex multi-locus trait, an increase of 0.32 piglets born is substantial for current pig breeding programs. This indicated that the introgressed *KATNAL1* haplotype has been favored and intensively selected by breeders, contributing to the formation of excellent reproductive traits in FLW pigs.

AHR **haplotypes that associate with increased litter size were likely introgressed from ECN pigs into LW pigs**

In 2014, Bosse *et al.* [11] found that Chinese haplotypes in a 6.8-Mb region on chromosome 9 containing the *AHR* gene were introgressed into European pigs and were preferentially selected to increase fertility during the development of LW pigs. We also conducted a shared haplotype test (rIBD) between 121 Chinese pigs and 64 LW pigs in this 6.8-Mb region. We confirmed the presence of Chinese-derived haplotypes in European pigs including FLW pigs, with a strong introgression signal at the *AHR* locus (SSC9: 92.25–97.45 Mb in Sscrofa10.2 and 83.90–88.40 Mb in Sscrofa11.1) (**supplementary fig. S11**). To explore the geographic origin of the introgressed Chinese *AHR* haplotypes, we first constructed a phylogenetic tree of all sequenced individuals around the *AHR* region, and surprisingly found that most of domestic pigs were

clustered together with small genetic distance but were divergent from European and Asian wild boars (**supplementary fig. S12a**). We further reconstructed and visualized haplotypes around the *AHR* gene (95.5–95.65 Mb in Sscrofa10.2 and 86.47–86.65 Mb in Sscrofa11.1) and found that most haplotypes of LW pigs were highly similar to those of Chinese EHL pigs and Tibetan pigs (**fig. 5a**). In an NJ-tree of this region, 15 FLW pigs gathered with EHL pigs and Tibetan pigs, defining a branch distinct from other Chinese breeds (**supplementary fig. S12b**). In addition, FLW pigs and EHL pigs had the smallest F_{ST} values with the exception of other LW pigs (**supplementary fig. S12c**). Given the geographic distance between Tibet and Europe and the lack of any historical records describing the importation of Tibetan pigs into Europe, we argue that Chinese derived *AHR* haplotypes in FLW pigs were most likely introgressed from ECN pigs such as EHL pigs.

The *AHR* haplotype was introgressed into Chinese pigs via ancient interspecies hybridization

We noticed that the *AHR* haplotypes of most Chinese pigs were highly similar, but were distinct from those of Asian and European wild boars (**figs. 5a)**. Moreover, a large proportion of Chinese domestic pigs, such as EHL pigs, had a smaller nucleotide distance from the OUT population than from Asian wild boars in the *AHR* region (**supplementary fig. S13**). This was unexpected, as we know that these domestic pigs originated from wild boars. One possible explanation was that the *AHR* haplotypes of many Chinese domestic pigs were not derived from Chinese wild boars but from another potentially extinct *Sus* species. To test this hypothesis, we constructed a haplotype network using 133 SNPs in the *AHR* gene (see Methods). Interestingly, we observed three distinct haplotype groups, one including the haplotype of Asian wild boars and Chinese domestic pigs, one comprised of European wild boars and European domestic pigs, and another was defined by haplotypes of Eurasian

domestic pigs and OUT individuals (**fig. 5b**). In addition, the most frequent haplotype (XVI) appeared 100 times in all 266 sequenced individuals, including 30 FLW pigs, 24 other LW pigs, 18 EHL pigs and 26 Tibetan pigs. This haplotype had a close phylogenetic relationship with the OUT haplotype XII but was divergent from the major haplotypes (II and VII) of Eurasian wild boars (**fig. 5b**), a pattern expected under introgression. Note that the nucleotide differences between the haplotype XVI and the OUT haplotype XII were only 7, in contrast to the 100 or so between haplotypes XVI and II and 93 between haplotypes XVI and VII (**fig. 5c**). These findings corroborated our assumption that haplotype XVI was introgressed from a divergent archaic *Sus* population into the ancestors of Chinese domestic pigs via naturally occurring interspecies hybridization, then introduced from ECN pigs into European domestic pigs through human-mediated transportation about 200–300 years ago, and thus, rarely existed in Eurasian wild boars.

To provide further evidence for a possible interspecies hybridization at the *AHR* locus, we selected 38 individuals from OUT, Asian wild boars (AWB), and ECN pigs (EHL as an example) to perform allele frequency difference analysis, and calculated the ratio of OUT SNPs in EHL pigs per window with a sliding window of 50 kb across the entire genome (see Methods). Of note, a total of 63 SNPs in a 50-kb window containing the *AHR* gene were potential archaic SNPs derived from the OUT population, accounting for 66% of the total SNPs in this window. This ratio was the largest one in all 45,429 windows genome-wide (**fig. 5d**). Furthermore, we used an improved ABBA-BABA method (f_D) for gene flow analysis (see Methods). Interestingly, we detected an extreme outlier signal that the window containing the *AHR* gene had an f_D value greater than 0.8 corroborated by a significant outlier of the nucleotide distance (D_{xy}) between EHL pigs and AWB (**fig. 5e**). In addition, the nucleotide distance of EHL pigs versus AWB and FLW pigs versus European wild boars reached 2.05 and 2.64, respectively. In contrast, the nucleotide distance of EHL pigs versus OUT animals

and FLW pigs versus OUT animals were only 1.51 and 1.41, respectively (**supplementary fig. S13**). Altogether, our data strongly support archaic introgression at the *AHR* locus.

We noted that the introgressed haplotype XVI was desirable for increasing the EBV-TNB of both FLW pigs (**fig. 5f**) and EHL pigs (**fig. 5g**). By genotyping the haplotype tag SNPs and one-way analysis of variance (see Methods), we found that homozygous carriers of the archaic *AHR* haplotype (XVI) had 0.24 higher EBV-TNB than heterozygous carriers (*P* = 0.001, **supplementary table S6**) in EHL pigs. Moreover, the introgressed archaic *AHR* haplotype was significantly associated with increased EBV-TNB in FLW sows, with an additive effect value of 0.25 (P = 2.39 e^{-05}; **fig. 5f, supplementary table S7**), which was in agreement with the report of Bosse *et al*.[11]. Similar to the *KATNAL1* and *GOLM1-NAA35* regions, the LD value of FLW pigs at the *AHR* gene region ranked in the top 7% (significant outlier) of all 10,000 bootstrap values (*P* = 0.03, **supplementary fig. S14**). We also detected a significant iHS selection signal within the FLW pig population (**supplementary fig. S15**). These findings enabled us to conclude that the archaic *AHR* haplotype had been under a preferential selection to improve the fertility of FLW pigs.

Discussion

Introgression of both SCN and ECN pig DNA contributed to the genetic improvement of European modern pig breeds

European and Asian domestic pigs were independently domesticated from European and Asian wild boars, respectively, nearly 10,000 years ago [3, 5, 6]. In this study, population genetics analyses confirmed striking genetic differences between Chinese and European domestic pigs and uncovered obvious genetic differentiation between SCN and ECN pigs, which represent two ancestral lineages of Chinese pigs. Of note, we identified Chinese haplotypes in FLW pigs, which were introgressed from both SCN and ECN pigs. We inferred that the introgression

events occurred 220–310 years ago, which was in accordance with historical records that SCN pigs were transported to England through the Guangzhou port during the first Industrial Revolution [7]. Our results also supported the speculation that ECN pigs were introduced into Europe, likely through the Shanghai and Ningbo ports, in the decades before the Qing Dynasty imposed a ban on the sea in 1757. Thus, we believe that both SCN and ECN pigs were introduced to Europe to improve the production performance of local breeds, contributing to the development of modern European commercial pig breeds. Taking the *GOLM1-NAA35* and *KATNAL1* loci as examples, the introgressed *GOLM1-NAA35* haplotype from SCN pigs was beneficial for improving disease resistance in FLW pigs, and the introgressed *KATNAL1* haplotype from ECN pigs was favorable for boar fertility and provided genetic variations for the development of high-fecundity FLW pigs. These findings not only advance our understanding of the breeding history of modern European commercial pig breeds but also provides insights into the genetic mechanisms underlying economically important traits in pigs.

Both naturally occurring interspecies hybridization and human-driven crossbreeding played important roles in the development of global pig breeds

In recent years, emerging reports have shown that interspecies hybridization played an important role in adaptive evolution of mammals. For example, the Denisova-like *EPAS1* haplotype help Tibetans to adapt to the high-altitude hypoxia environments [18]. Admixture with the yak enabled Tibetan cattle to quickly obtain favorable *EGPN1* alleles for high-altitude adaptation [19]. We reported previously on an archaic adaptive introgression on the X chromosome that contributed to the adaptation of North Chinese pigs to high-latitude cold environments [20]. Here, we show that the *AHR* haplotype associated with increased sow litter size was derived from an archaic population. It was first introgressed into Chinese pigs via interspecies hybridization. Then it was introduced from ECN pigs into European pigs, such as

the Large White breed, through human-mediated transportation and hybridization some 200–300 years ago. It has further experienced preferential selection, presumably during the past decades, and is present at high frequency in FLW pigs, contributing to the improvement of the reproductive performance of this breed. Thus, this study provides another example of the archaic adaptive introgression of domestic animals. It also shows that both naturally occurring interspecies hybridization and human-driven crossbreeding play important roles in the development of global pig breeds, illustrating a complex breeding history of domestic pigs.

Materials and Methods

Samples

All procedures used for this study and involving animals were in compliance with guidelines for the care and utility of experimental animals established by the Ministry of Agriculture of China. The ethics committee of Jiangxi Agricultural University approved this study. This study utilized genome-wide re-sequencing data from 266 animals (**supplementary table S1**), of which 153 pigs were re-sequenced for this study and 113 genome sequence datasets were downloaded from public databases (Registration Nos. ERP001813 [21], PRJEB9922 [22], and SRP047260 [23]). Among the 153 pigs, 36 were FLW sows and were collected from the Guangdong WENS Food Company (24 individuals) and Jiangxi Lvhuan Animal Husbandry Company (12 individuals). The 36 FLW sows were selected according to the following criteria. First, we calculated the relationship coefficients of all individuals in the nucleus populations of the two companies using DMU software [24] and pedigree records. Then we selected sows with a small relationship coefficient and excellent litter sizes (TNB more than 16). Finally, we chose 36 prolific individuals with distant kinship according to a phylogenetic relationship network constructed by Cytoscape v3.2.1 [25] (**supplementary fig. S1**). In total, there were 27 wild boars from China and Europe, 7 outgroup individuals, 121 pigs from Chinese indigenous

breeds, and 111 pigs from European commercial breeds. According to the geographic distribution, Chinese domestic pigs were divided into ECN (37) pigs, SCN (20) pigs, SWCN (36) pigs, and NCN (28) pigs (see **supplementary table S1** for details). In addition, whole-genome sequence data of 28 LW pigs was downloaded from public databases, with 14 individuals submitted by Seoul National University [23] and another 14 individuals submitted by Wageningen University [21]. To identify the source of these 28 LW pigs, we downloaded the Illumina 60K chip SNP data set of 76 LW pigs [26], including 20 Dutch Large White pigs (NLW), 16 Danish Large White pigs (DLW), 20 Chinese Large White pigs (CLW), and 20 American Large White pigs (ALW). Next, we retrieved the same 60K chip SNPs from the whole-genome sequence data sets of the 28 LW pigs. We filtered out SNPs with an MAF less than 0.05, a call rate less than 90%, and an LD (r^2) value more than 0.3 using PLINK v1.9 [27], and we performed PCA and NJ-tree analyses using the remaining SNPs to identify the origin of the 28 LW pigs (**supplementary fig. S2**).

Whole-genome sequencing and SNP calling

We extracted genomic DNA from the ear tissues of 153 pigs using a routine phenol/chloroform protocol, and eligible samples were delivered to the Novogene company (Beijing, China). Sequencing was performed on Hiseq 2000 or 2500 instruments (Illumina, La Jolla, CA USA). The sequencing libraries were constructed with 125 bp paired ends (PE125), a 500 bp average insert fragment size, and a fragment size less than 800 bp. The genome sequencing coverage of each individual was at least 20×, with a minimum data of 60 G.

Quality control: We obtained the raw sequencing data from Hiseq sequencing platform using raw image data. We obtained clean data for performing downstream analysis after performing the following steps: (1) removal of the linker sequence, (2) retention of reads with Q20 of more than 90% (the probability of base recognition correct rate higher than 99%) and

Q30 of more than 85% (the probability of base recognition correct rate higher than 99.9%)[28], (3) culling of short repeat DNA segments, and (4) filtering reads with three consecutive "N".

Mutation detection: We established a reference genome index of Sscrofa 10.2 [6] using the index function in BWA v0.7.12 [29]. We blasted paired-end reads against the index using an algorithm from BWA and obtained binary bam files from sam files by SAMtools v1.4 [30]. We used samblaster v0.1.22 [31] to reject redundancy information and calculated the alignment rate between re-sequencing data and the reference genome, as well as coverage and sequencing depth. We sorted binary bam files via GATK v3.7 [32]. We used the HaplotypeCaller function for mutation detection across each chromosome of each individual and obtained a SNP data set of the 266 individuals by deleting InDel information. We filtered out SNPs with an MAF less than 0.01 and a call rate less than 90% using PLINK v1.9 [27]. We used the remaining 32.7 million SNPs in the data set for subsequent statistical analysis.

Population genetic analysis

First, we generated a SNP data set with an MAF more than 0.05 and a call rate more than 90% from autosomal SNPs from 259 pigs (*Sus scrofa*) excluding seven OUT individuals. Second, we pruned SNPs with an LD (r^2) decay of more than 0.3 in each window with 50 SNPs using the command indep-pairwise (50 10 0.3) in PLINK v1.9 [27]. Then four principal components of each individuals were estimated using --pca command in GCTA software [33]. The average shared allele (1-Dst) distance matrix between individuals was constructed using the command --distance-matrix in PLINK v1.9. A rootless NJ tree was constructed via phylip v3.69 [34] and was visualized with FigTree v1.42. We also explored the unbiased estimation method proposed by Weir and Cockerham to calculate the genetic differentiation (F_{ST}[35]) matrix between 14 Chinese pig breeds and 6 European pig breeds using the --fst command in PLINK v1.9 ([27]. Then, we constructed an interbreed NJ tree using phylip v3.69 [34]. ADMIXTURE [36] was

used to estimate the ancestral lineage composition under default parameters. First, we removed the OUT group and populations with fewer than five individuals. Then we randomly selected six individuals from the remaining 21 populations and filtered out SNPs with an MAF of less than 0.05, an LD (r^2) of more than 0.3, and call rates less than 90%. Finally, we used a data set with 125 individuals and 658,601 SNPs to analyze the ancestral lineage composition patterns. In addition, we utilized TreeMix v1.12 [37] to infer the genetic differentiation among populations. We set OUT as the outgroup population, excluding populations with fewer than six samples and SNPs with an MAF less than 0.05 and a call rate less than 90%. We used the data set with 19,282,590 SNPs to estimate genetic differentiation among 21 populations under no migration events via TreeMix v1.12 [37].

Introgression analysis

We detected the introgression signals between Chinese pigs (ECN and SCN pigs) and FLW pigs using an IBD sharing approach [11]. First, we used a data set with 266 individuals and approximately 20 million SNPs to phase haplotypes using the fastPhase function [38] in Beagle v4.0 and to detect IBD fragments in each individual using the fastIBD function [39]. Then we divided the whole genome into numbers of 50 kb windows (25 kb sliding) and calculated the shared IBD haplotype numbers between two populations (FLW vs. European wild boars (EWB), FLW vs. ECN, and FLW vs. SCN) in each window. We phased the haplotypes and detected the IBD regions independently 10 times and then normalized the IBD values (nIBD). The nIBD values ranged from 0 (no shared IBD detected) to 1 (all individuals shared the IBD haplotype). Finally, we used the rIBD (relative frequency of IBD) statistic to measure the shared IBD between FLW pigs and SCN or ECN pigs, respectively ($rIBD_{FLW\text{-}SCN} = nIBD_{FLW\text{-}SCN} - nIBD_{FLW\text{-}EWB}$, $rIBD_{FLW\text{-}ECN} = nIBD_{FLW\text{-}ECN} - nIBD_{FLW\text{-}EWB}$), where a positive rIBD indicated potential introgression and 5% empirical distribution in the far right tail were set as

the significance threshold. For genomic regions showing strong rIBD introgression signals in FLW pigs, we further estimated F_{ST} between FLW pigs and European wild boars, as well FLW pigs and Chinese pigs (SCN pigs or ECN pigs), respectively. We also constructed haplotype networks using SNPs with a MAF of greater than 0.05 and call rates of greater than 90% at the *GOLM1-NAA3* (298 SNPs), *KATNAL1* (529 SNPs) and *AHR* (68 SNPs) loci. We explored the fastPhase function with 1000 iterations in Beagle v4.0 [39] to phase haplotypes and used the "haploNet" command in the R package "pegas" [40] to calculate the pairwise differences between haplotypes. We selected SNPs with an MAF of greater than 0.05, a call rate of greater than 90% and an LD (r^2) < 0.3 using PLINK v1.9 [27], and then explored the selected SNPs to estimate the admixture time between populations via ALDER v1.0.3 under default parameters [13]. In short, we used the "convert" function in EIGENSTRAT [41] to convert the data format. We set FLW as a mixed population, EWB and SCN as one reference population, EWB and ECN as another reference population, and five years as one generation.

Signature of selection

We used the data set that excluded SNPs with an MAF of less than 0.05 and a call rate less than 90% in the whole-genome SNPs data set of 36 FLW pigs to calculate the correlation coefficient (r^2) of each SNP pair in a target region using the commands --r2 inter-chr --ld-window-r2 0 in PLINK v1.9 [40], and we used the average r^2 as the LD value in the region. Meanwhile, we randomly selected 10,000 regions with the same size as the target region across the genome, and we calculated the average r^2 of each region in the 36 FLW pigs. Finally, we visualized the density curve of the 10,000 bootstrap values using R. Furthermore, we used commands --ihs [42] and --xpehh [43] under default parameters in selscan [44] software to detect the signatures of selection in 50 kb windows with a step size of 25 kb in FLW pigs.

Archaic introgression test

We used an improved f_D method [45] via the ABBA-BABA statistic to detect potential archaic introgression in the *AHR* region between the OUT population and EHL pigs. The calculation formulas are as follows:

$$C_{ABBA}(i) = (1 - p_{i1})p_{i2}p_{i3}(1 - p_{i4}),$$

$$C_{BABA}(i) = (1 - p_{i1})p_{i2}(1 - p_{i3})p_{i4},$$

$$\mathrm{S}(p_1, p_2, p_3, O) = \sum[C_{ABBA}(i) - C_{BABA}(i)],$$

$$f_D = \frac{\mathrm{S}(p_1, p_2, p_3, O)}{\mathrm{S}(p_1, p_D, p_D, O)},$$

where p_{ij} indicates the *ith* frequency of the derived allele in the *jth* population, S represents the sum of the difference between ABBAs and BABAs, and $p_D$means populations with higher frequency of the derived allele (P2 or P3). According to the NJ tree, Asian wild boars, EHL, and five OUT individuals (two Sumatras, one *Sus barbatus*, one *Sus verrucosus*, and one *Sus* cebifrons), and one OUT individual (*Phacochoerus africanus*) were set as P1, P2, P3, and O, respectively.

Nucleotide differences within (D_x) and among (D_{xy}) populations were calculated using follow formulas [20]:

$$D_x = \frac{2}{n_x(n_x - 1)l} \sum_{i=1}^{n_x - 1} \sum_{j=i+1}^{n_x - 1} k_{ij},$$

$$D_{xy} = \frac{2}{n_x n_y l} \sum_{i=1}^{n_x} \sum_{j=1}^{n_y} k_{ij},$$

where k_{ij} indicates the difference number of haplotype alleles between the *ith* allele the *jth* allele in a target region, n_x and n_y represent the number of haplotypes in population *x* and *y*, and *l* indicates the number of bases that are valid in the target area (the number of bases other than N in the reference sequence). In addition, 38 individuals were selected from the OUT group (7), Asian wild boar (10), and Erhualian populations (21) and we pruned SNPs with an MAF of less than 0.05 and a call rate of less than 90%, leaving 14,333,796 SNPs. We used 50

kb windows with a sliding filter size of 25 kb with less than 10 SNPs to calculate the allele ratio ($r_D = n_i/n_o$) in each window, where n_o indicated the number of SNPs with an allele frequency of more than 0.7 in each window in the OUT group, and n_i represents the number of SNPs with an allele frequency more than 0.6 in EHL pigs as well less than 0.15 in Chinese wild boars. The r_D of the *AHR* region is shown using a probability density curve.

Haplotype association analysis

The *GOLM1-NAA35* locus: We detected the serum IL-6 levels in 54 mature FLW sows at an age of 2–2.5 years from the same farm using the Porcine IL-6 ELISA Kit (Shanghai Keshun Biological Technology, China). The concentration of each individual was determined from the averaged repeat of three trials per individual. Meanwhile, we selected two tag SNPs to distinguish the introgressed haplotypes (VII and VIII) from the other haplotype in the *GOLM1-NAA35* region in FLW pigs (**fig. 3e**). The tag SNPs were genotyped by Sanger sequencing PCR products amplified with specific primers (**supplementary table S5**). A Student's *t*-test was used to detect the association between haplotypes and the serum IL-6 concentrations (log2 (IL-6 values)).

The *KATNAL1* locus: We collected 765 FLW sows and 31 FLW boars from the Jiangxi Lvhuan Farming Group. First, we filtered parities with litter size less than five piglets. Then we set estrus, year, season, parity and pregnancy duration as fixed effects, and mating boars and random sow effects as random effects. We then estimated the EBV for TNB of 765 FLW pigs via DMU software [24] and pedigree information. Next, we genotyped eight tagged SNPs to distinguish each *KATNAL1* haplotype in the 31 FLW boars by PCR amplification and Sanger sequencing with primers listed in **supplementary table S8**. We denoted the introgressed XXVII haplotype from ECN pigs as Q (**fig. 4e**) and the other haplotypes as q (**supplementary**

table S9). Finally, we used Student’s *t*-test to test the association between *KATNAL1* haplotypes and the average EBV-TNB of mating sows of the 31 FLW boars.

The *AHR* locus: We genotyped two tagged SNPs representing the *AHR* haplotypes for 344 FLW sows by PCR amplification and Sanger sequencing with primers listed in **supplementary table S6**. We identified 230 *QQ* sows homozygous for the introgressed haplotype, 36 *Qq* sows and 78 *qq* sows who were missing the introgressed haplotypes (**supplementary table S6**). Then we tested the association between the *AHR* haplotypes and the EBV-TNB of the 344 sows using single-factor analysis of variance. Furthermore, we collected 221 Erhualian sows with multiparity records from Jiangsu Provence and calculated the EBV-TNB of these sows using DMU software and pedigree information as mentioned above. We genotyped a tag SNP in the *AHR* region by Sanger sequencing PCR products with specific primers (**supplementary table S7**). We detected 176 *QQ* sows homozygous for the introgressed haplotype and 45 heterozygous (Qq) sows. We used a Student's *t*-test to examine the association between *AHR* haplotypes and EBV-TNB in Erhualian sows.

Acknowledgements

This study is supported by the Natural Science Foundation of China (31525023) and the National Key Research Project of China (2016ZX08006-5).

Author Contributions

J.R. and L.H. designed the study and analyzed the data. J.R., H.C. and L.H. wrote the paper. H.C., M.H., and B.Y. performed the bioinformatic analyses. H.C., M.H., Z.D. Z.W. and Y.H. collected data and performed sequencing and genotyping experiments. We thank LetPub (www.letpub.com) for its linguistic assistance during the preparation of this manuscript.

Competing financial interests

The authors declare no competing financial interests

References

1. Giuffra E, Kijas JM, Amarger V, Carlborg O, Jeon JT and Andersson L. The origin of the domestic pig: independent domestication and subsequent introgression. Genetics. 2000;154 4:1785-91.
2. Kijas JM and Andersson L. A phylogenetic study of the origin of the domestic pig estimated from the near-complete mtDNA genome. Journal of Molecular Evolution. 2001;52 3:302-8.
3. Larson G, Dobney K, Albarella U, Fang M, Matisoosmith E, Robins J, et al. Worldwide phylogeography of wild boar reveals multiple centers of pig domestication. Science. 2005;307 5715:1618-21.
4. Frantz LA, Schraiber JG, Madsen O, Megens HJ, Bosse M, Paudel Y, et al. Genome sequencing reveals fine scale diversification and reticulation history during speciation in Sus. Genome Biol. 2013;14 9:R107. doi:10.1186/gb-2013-14-9-r107.
5. Frantz L, Meijaard E, Gongora J, Haile J, Groenen MAM and Larson G. The evolution of Suidae. Annual Review of Animal Biosciences. 2016;4 1:61.
6. Groenen MA, Archibald AL, Uenishi H, Tuggle CK, Takeuchi Y, Rothschild MF, et al. Analyses of pig genomes provide insight into porcine demography and evolution. Nature. 2012;491 7424:393.
7. Wang L, Chan YF, Russell P, Mauceli E, Johnson J, Swofford R, et al. Animal Genetic Resources in China: pigs (ed. China National Commission of Animal Genetic Resources). China Agriculture Press; 2011.
8. Rischkowsky B and Pilling D. The State of the World's Animal Genetic Resources for Food and Agriculture (Food and Agriculture Organization (FAO)). 2007.
9. Briggs HM and Briggs DM. Modern Breeds of Livestock. 4th ed. Macmillan Publishing Co.,Inc.; 1980.
10. Zhang W. Introduction to Large White pigs. SWINE PRODUCTION. 2011; 1:61-4.
11. Bosse M, Megens HJ, Frantz LA, Madsen O, Larson G, Paudel Y, et al. Genomic analysis reveals selection for Asian genes in European pigs following human-mediated introgression. Nat Commun. 2014;5:4392. doi:10.1038/ncomms5392.
12. Ramos AM, Crooijmans RP, Affara NA, Amaral AJ, Archibald AL, Beever JE, et al. Design of a high density SNP genotyping assay in the pig using SNPs identified and characterized by next generation sequencing technology. PLoS One. 2009;4 8:e6524. doi:10.1371/journal.pone.0006524.

13. Loh PR, Lipson M, Patterson N, Moorjani P, Pickrell JK, Reich D, et al. Inferring admixture histories of human populations using linkage disequilibrium. Genetics. 2013;193 4:1233.
14. Kladney RD, Bulla GA, Guo L, Mason AL, Tollefson AE, Simon DJ, et al. GP73, a novel Golgi-localized protein upregulated by viral infection. Gene. 2000;249 1–2:53-65.
15. Li Y, Oosting M, Deelen P, Ricañoponce I, Smeekens S, Jaeger M, et al. Inter-individual variability and genetic influences on cytokine responses to bacteria and fungi. Nature Medicine. 2016;22 10:1192.
16. Liu S, Huang S, Chen F, Zhao L, Yuan Y, Francis SS, et al. Genomic Analyses from Non-invasive Prenatal Testing Reveal Genetic Associations, Patterns of Viral Infections, and Chinese Population History. Cell. 2018;175 2:347-59 e14. doi:10.1016/j.cell.2018.08.016.
17. Smith LB, Milne L, Nelson N, Eddie S, Brown P, Atanassova N, et al. KATNAL1 Regulation of Sertoli Cell Microtubule Dynamics Is Essential for Spermiogenesis and Male Fertility. Plos Genetics. 2012;8 5:e1002697.
18. Huertasánchez E, Jin X, Bianba Z, Peter BM, Vinckenbosch N, Liang Y, et al. Altitude adaptation in Tibetans caused by introgression of Denisovan-like DNA. Nature. 2014;512 7513:194-7.
19. Wu DD, Ding XD, Wang S, Wójcik JM, Zhang Y, Tokarska M, et al. Pervasive introgression facilitated domestication and adaptation in the Bos species complex. Nature ecology & evolution. 2018.
20. Ai H, Fang X, Yang B, Huang Z, Chen H, Mao L, et al. Adaptation and possible ancient interspecies introgression in pigs identified by whole-genome sequencing. Nat Genet. 2015;47 3:217-25. doi:10.1038/ng.3199.
21. Rubin CJ, Megens HJ, Martinez Barrio A, Maqbool K, Sayyab S, Schwochow D, et al. Strong signatures of selection in the domestic pig genome. Proc Natl Acad Sci U S A. 2012;109 48:19529-36. doi:10.1073/pnas.1217149109.
22. Frantz LA, Schraiber JG, Madsen O, Megens HJ, Cagan A, Bosse M, et al. Evidence of long-term gene flow and selection during domestication from analyses of Eurasian wild and domestic pig genomes. Nature Genetics. 2015;47 10:1141.
23. Sunjin M, Tae-Hun K, Kyung-Tai L, Woori K, Taeheon L, Si-Woo L, et al. A genome-wide scan for signatures of directional selection in domesticated pigs. BMC Genomics,16,1(2015-02-25). 2015;16 1:1-12.
24. Madsen P, Sørensen P, Su G, Damgaard LH, Thomsen H and Labouriau R. DMU - a package for analyzing multivariate mixed models. In: *Proceedings of the 8th World Congress on Genetics Applied to Livestock Production, Belo Horizonte, Minas Gerais, Brazil, 13-18 August, 2006* 2014, pp.27-11.

25. Shannon P, Markiel A, Ozier O, Baliga NS, Wang JT, Ramage D, et al. Cytoscape: A Software Environment for Integrated Models of Biomolecular Interaction Networks. Routledge; 2003.
26. Yang B, Cui L, Perez-Enciso M, Traspov A, Crooijmans R, Zinovieva N, et al. Genome-wide SNP data unveils the globalization of domesticated pigs. Genet Sel Evol. 2017;49 1:71. doi:10.1186/s12711-017-0345-y.
27. Chang CC, Chow CC, Tellier LC, Vattikuti S, Purcell SM and Lee JJ. Second-generation PLINK: rising to the challenge of larger and richer datasets. Gigascience. 2015;4:7. doi:10.1186/s13742-015-0047-8.
28. Ewing B and Green P. Base-calling of automated sequencer traces using phred. II. Error probabilities. Genome Res. 1998;8 3:186-94.
29. Li H and Durbin R. Fast and accurate short read alignment with Burrows-Wheeler transform. Bioinformatics. 2009;25 14:1754-60. doi:10.1093/bioinformatics/btp324.
30. Li H. A statistical framework for SNP calling, mutation discovery, association mapping and population genetical parameter estimation from sequencing data. Bioinformatics. 2011;27 21:2987-93. doi:10.1093/bioinformatics/btr509.
31. Faust GG and Hall IM. SAMBLASTER: fast duplicate marking and structural variant read extraction. Bioinformatics. 2014;30 17:2503-5. doi:10.1093/bioinformatics/btu314.
32. McKenna A, Hanna M, Banks E, Sivachenko A, Cibulskis K, Kernytsky A, et al. The Genome Analysis Toolkit: a MapReduce framework for analyzing next-generation DNA sequencing data. Genome Res. 2010;20 9:1297-303. doi:10.1101/gr.107524.110.
33. Yang J, Lee SH, Goddard ME and Visscher PM. GCTA: a tool for genome-wide complex trait analysis. Am J Hum Genet. 2011;88 1:76-82. doi:10.1016/j.ajhg.2010.11.011.
34. Cummings MP. PHYLIP (phylogeny inference package). Dictionary of Bioinformatics and Computational Biology. 2004.
35. Weir BS and Cockerham CC. Estimating F-Statistics for the Analysis of Population Structure. Evolution. 1984;38 6:1358-70. doi:10.1111/j.1558-5646.1984.tb05657.x.
36. Alexander DH, Novembre J and Lange K. Fast model-based estimation of ancestry in unrelated individuals. Genome Res. 2009;19 9:1655-64. doi:10.1101/gr.094052.109.
37. Pickrell JK and Pritchard JK. Inference of population splits and mixtures from genome-wide allele frequency data. PLoS Genet. 2012;8 11:e1002967. doi:10.1371/journal.pgen.1002967.
38. Browning SR and Browning BL. Rapid and accurate haplotype phasing and missing-data inference for whole-genome association studies by use of localized haplotype clustering. Am J Hum Genet. 2007;81 5:1084-97. doi:10.1086/521987.
39. Browning BL and Browning SR. A fast, powerful method for detecting identity by descent. Am J Hum Genet. 2011;88 2:173-82. doi:10.1016/j.ajhg.2011.01.010.

40. Paradis E. pegas: an R package for population genetics with an integrated-modular approach. Bioinformatics. 2010;26 3:419-20. doi:10.1093/bioinformatics/btp696.
41. Price AL, Patterson NJ, Plenge RM, Weinblatt ME, Shadick NA and Reich D. Principal components analysis corrects for stratification in genome-wide association studies. Nat Genet. 2006;38 8:904-9. doi:10.1038/ng1847.
42. Voight BF, Kudaravalli S, Wen X and Pritchard JK. A map of recent positive selection in the human genome. PLoS Biol. 2006;4 3:e72. doi:10.1371/journal.pbio.0040072.
43. Sabeti PC, Varilly P, Fry B, Lohmueller J, Hostetter E, Cotsapas C, et al. Genome-wide detection and characterization of positive selection in human populations. Nature. 2007;449 7164:913-8. doi:10.1038/nature06250.
44. Szpiech ZA and Hernandez RD. selscan: An Efficient Multithreaded Program to Perform EHH-Based Scans for Positive Selection. Molecular Biology & Evolution. 2014;31 10:2824-7.
45. Martin SH, Davey JW and Jiggins CD. Evaluating the use of ABBA-BABA statistics to locate introgressed loci. Mol Biol Evol. 2015;32 1:244-57. doi:10.1093/molbev/msu269.

Figure Legends

Fig. 1. Population relationship and structure. (**a**) Principal component analysis of Chinese and European pigs. ECN, East Chinese pigs; NCN, North Chinese pigs; SCN, South Chinese pigs; SWCN, Southwest Chinese pigs; EUD, European domestic pigs. (**b**) Neighbor-joining (NJ) tree based on an identity-by-state matrix among individuals. (**c**) NJ tree based on an Fst matrix between populations. (**d**) Population structure of Chinese and European pigs revealed by ADMIXTURE analysis. MIN, Min pigs; HT, Hetao pigs; LWH, Laiwu pigs; EHL, Erhualian pigs; MS, Meishan pigs; JH, Jinhua pigs; GST, Tibetan pigs (gansu)； SCT, Tibetan pigs (Sichuan); YNT, Tibetan pigs (Yunnan); TT, Tibetan pigs (Tibet); WZS, Wuzhishan pigs; LUC, Luchuan pigs; BMX, Bamaxiang pigs; XIANG, Xiang pigs; AWB, Asian wild boars; OUT, outgroup; EWB, European wild boars; HMP, Hampshire; DU, Duroc; LR, Landrace; PI, Pietrain; WDU, White Duroc; WLW, Dutch Large White pigs; KLW, Korea Large White pigs; FLW, French Large White pigs.

Fig. 2. Introgressed Chinese haplotypes in French Large White pigs. (**a**) Manhattan plot of rIBD values between French Large White (FLW) and South Chinese (SCN) pigs (positive value) or European wild boars (EWB) (negative value). The red dashed line indicates the top 5% significance threshold. (**b**) Manhattan plot of rIBD values between FLW and East Chinese (ECN) pigs (positive value) or EWB (negative value). (**c**) Venn diagram of introgressed DNA (50 Kb windows) from SCN and ECN pigs in FLW pigs. (**d**) Venn diagram of genes in the introgressed regions from SCN and ECN pigs in FLW pigs. (**e**) Significantly enriched GO processes and KEGG pathways of introgressed genes in the introgressed regions from SCN and ECN pigs under selection in FLW pigs.

Fig. 3. Introgression at the *GOLM1-NAA35* locus. (**a**) rIBD values in a 2 Mb region harboring the *GOLM1-NAA35 g*ene. The brown dashed line indicates the 5% threshold line, and the *GOLM1-NAA35* region is indicated by grey dashed lines. (**b**) Genetic differentiation index (F_{ST}) between French Large White (FLW) and European wild boar (EWB) or South Chinese (SCN) pigs. (**c**) Haplotype heatmap in the *GOLM1-NAA35* region. Major and minor alleles in FLW pigs are indicated by beige and light blue, respectively. (**d**) Haplotype network in the *GOLM1-NAA35* region. Each circle represents a haplotype, and the size of the circle is proportional to the haplotype frequency. The line width and length represent the difference between haplotypes. Different colors represent pigs from different geographical regions. SWCN, Southwest Chinese pigs; NCN, North Chinese pigs; AWB, Asian (Chinese) wild boars; ECN, East Chinese pigs; EUD, European domestic pigs. (**e**) Selection signals in the *GOLM1-NAA35* region by XP-EHH analysis between FLW and other Large White pigs. The brown dashed line indicates the 5% threshold line. (**f**) Serum interleukin 6 (IL-6) contents of FLW pigs homozygous (QQ) or heterozygous (Qq) for the introgressed *GOLM1-NAA35* haplotypes. Student's *t*-test was employed to compute the *P*-value ($P = 0.015$).

Fig. 4. Introgression at the *KATNAL1* locus. (**a**) rIBD values in a 2 Mb region encompassing the *KATNAL1* gene. The brown dashed line indicates the 5% threshold line, and the *KATNAL1* region is indicated by grey dashed lines. (**b**) Genetic differentiation index (F_{ST}) between French Large White (FLW) and European wild boar (EWB) or East Chinese (ECN) pigs. (**c**) Haplotype heatmap of the *KATNAL1* region. Major and minor alleles in FLW pigs are indicated by beige and light blue, respectively. (**d**) Haplotype network in the *KATNAL1* region. The legend is the same as in Figure 3. (**e**) Selection signals by XP-EHH analysis between FLW and other Large White pigs. The brown dashed line indicates the 5% threshold line. (**f**) Estimated breeding values for total number of piglets born (TNB EBV) of FLW sows that mated with FLW boars

homozygous (QQ) or heterozygous (Qq) for the introgressed haplotypes. Student's *t*-test was employed to compute the *P*-value (P = 0.036).

Fig. 5. Archaic introgression at the *AHR* locus. (**a**) Haplotype heatmap of a 180 kb region on chromosome 9 (SSC9: 95.5 - 95.65 Mb on Sscrofa10.2 and 86.47 - 86.65 Mb on Sscrofa11.1). The *AHR* region is indicated by a red box. Major and minor alleles in FLW pigs are indicated by beige and light blue, respectively. (**b**) *AHR* haplotype network. Each pie chart represents one unique haplotype, and the radius of the pie chart is proportional to the five times of $\log_{10}$ (number of chromosomes with that haplotype). The width and length of the edges are proportional to the $\log_2$ (number of pairwise differences between the joined haplotypes) plus one, and the thinnest edge represents a difference of one mutation. Three different background colors represent the three different haplotype groups. Different colors represent pigs from different geographical regions. The full names of pig codes are given in the legend of Figure 3. (**c**) Haplotype difference between each *AHR* haplotype. (**d**) Distribution of the potential archaic SNPs. At these SNPs, the frequency difference between Erhualian and Chinese wild boars was greater than 0.45, and that between Erhualian pigs and outgroup animals was less than 0.1. The x-axis shows the ratio of the potential archaic SNPs in each 50 kb window, and the y-axis indicates the number of windows. The red line marks the ratio of the potential archaic SNPs in the window harboring the *AHR* gene. (**e**) Distribution of gene flow (f_D) and nucleotide distance (Dxy) statistics within nonoverlapping 50 kb windows across the genome. Dxy values between Erhualian pigs and Chinese wild boars are shown in the x-axis and f_D in the y-axis. The red dot, an extreme outlier, represents the window in which the *AHR* gene is located. (**f**) French Large White sows carrying the homozygous archaic *AHR* haplotype show significantly ($P = 2.39 \times 10^{-5}$) lower estimated breeding values for total number born EBV (TNB_EBV), compared with those who do not carry the archaic haplotype. (**g**)

Erhualian sows homozygously carrying the archaic haplotype (QQ) have higher (P = 0.0096) TNB_EBV than heterozygous carriers (qq).

Figure 1

Click here to access/download;Figure;Figure 1.tif

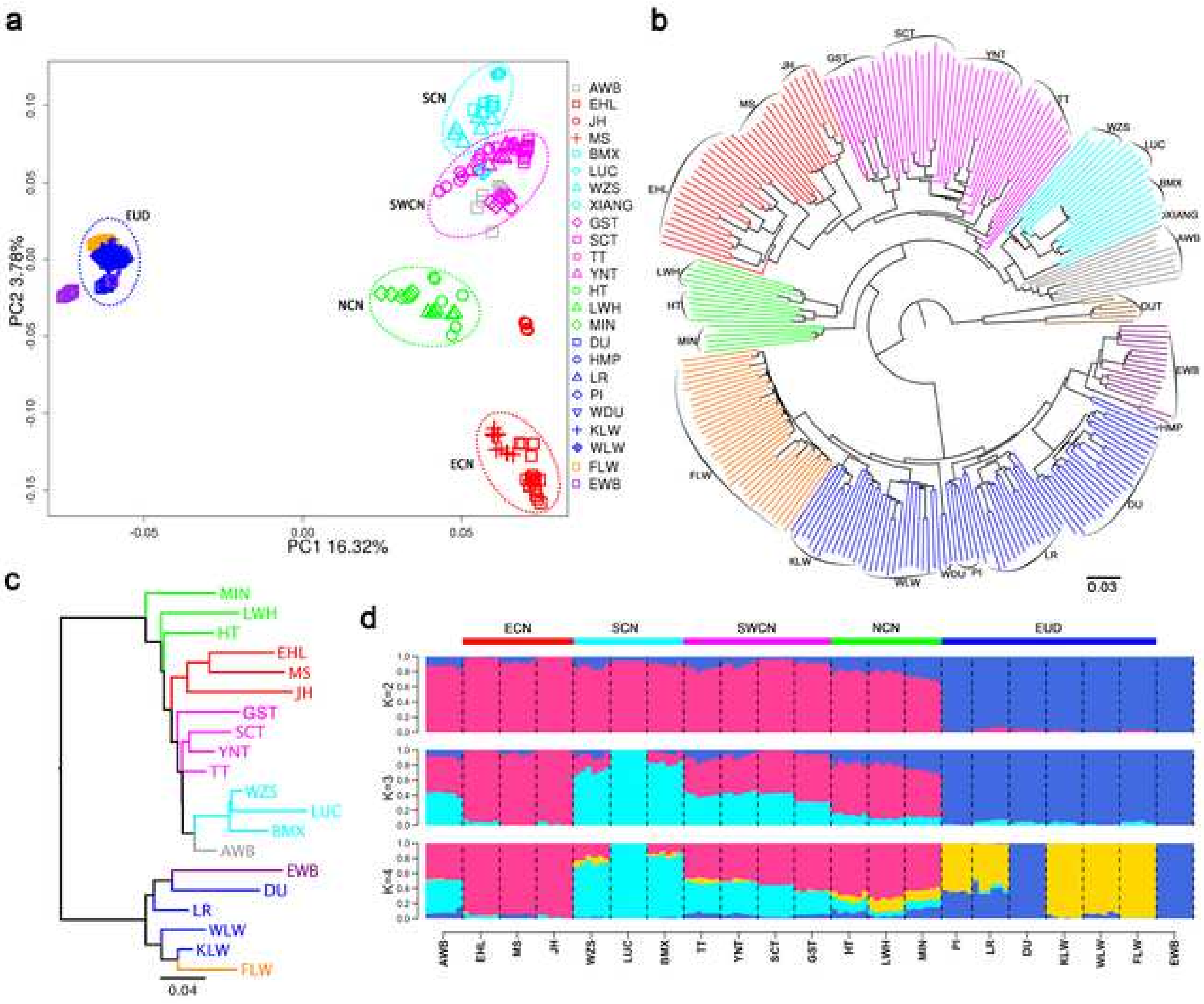

Figure 2

Click here to access/download;Figure;Figure 2.tif

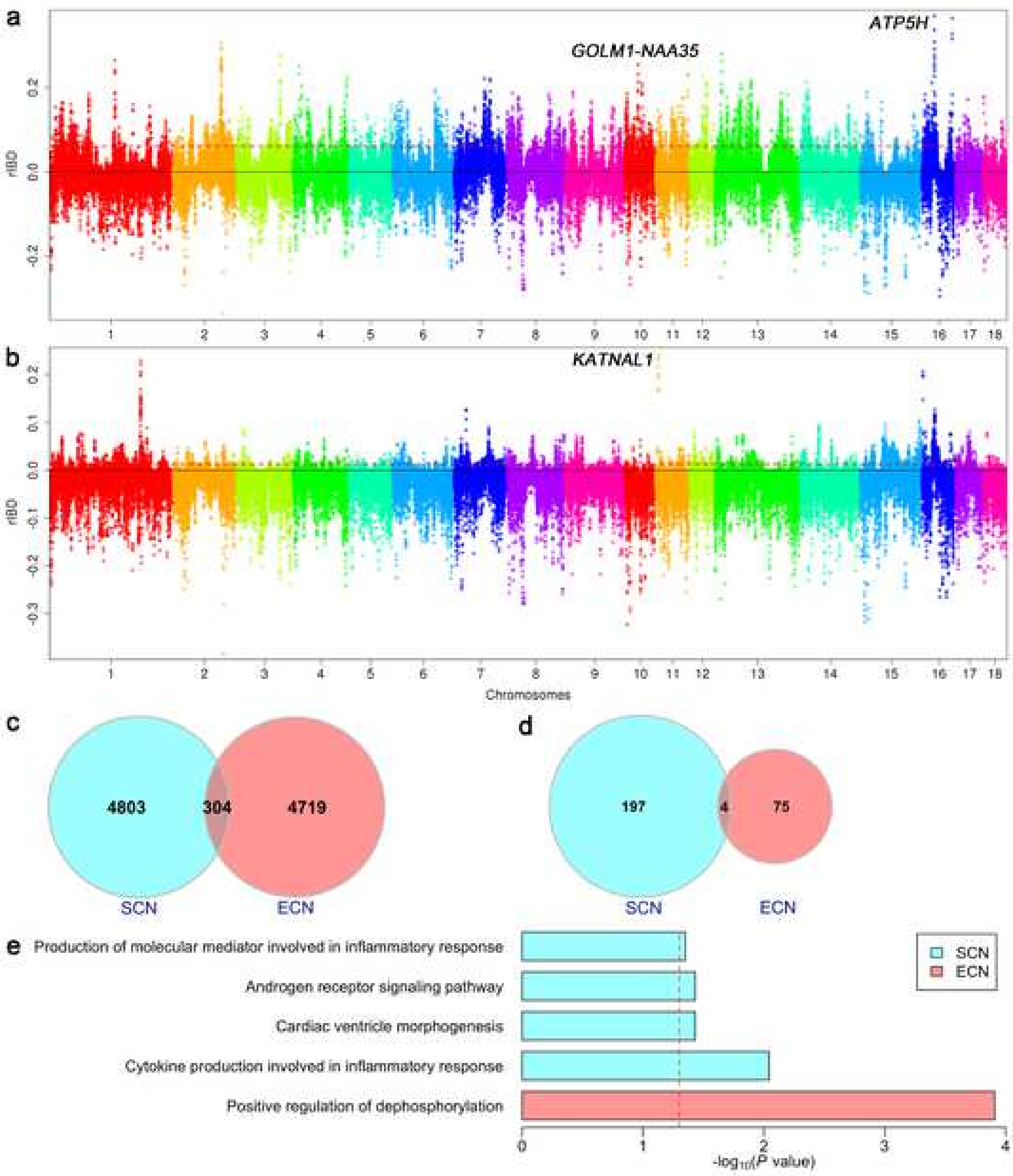

Figure 3

Click here to access/download;Figure;Figure 3.tif

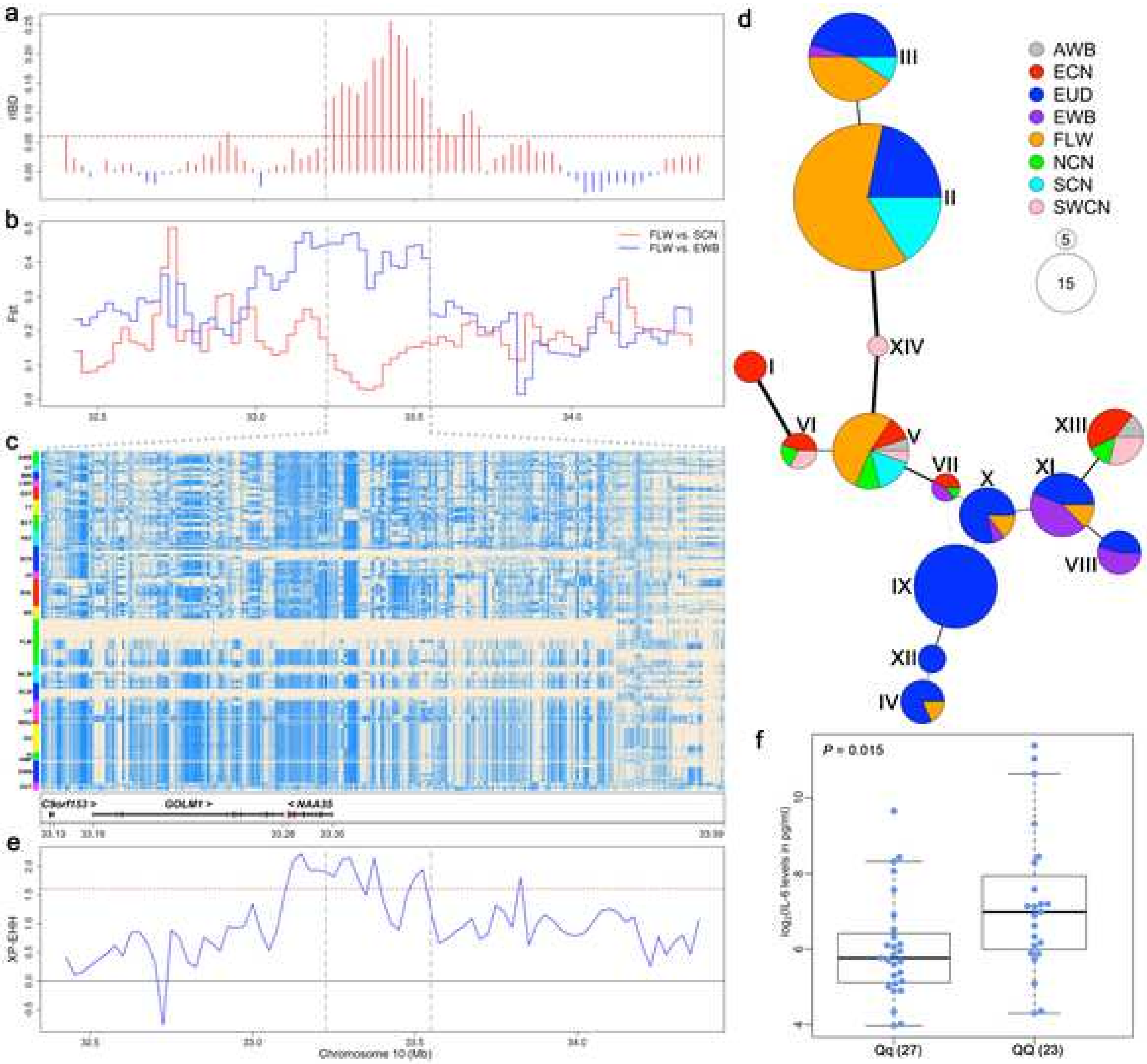

Figure 4

Click here to access/download;Figure;Figure 4.tif

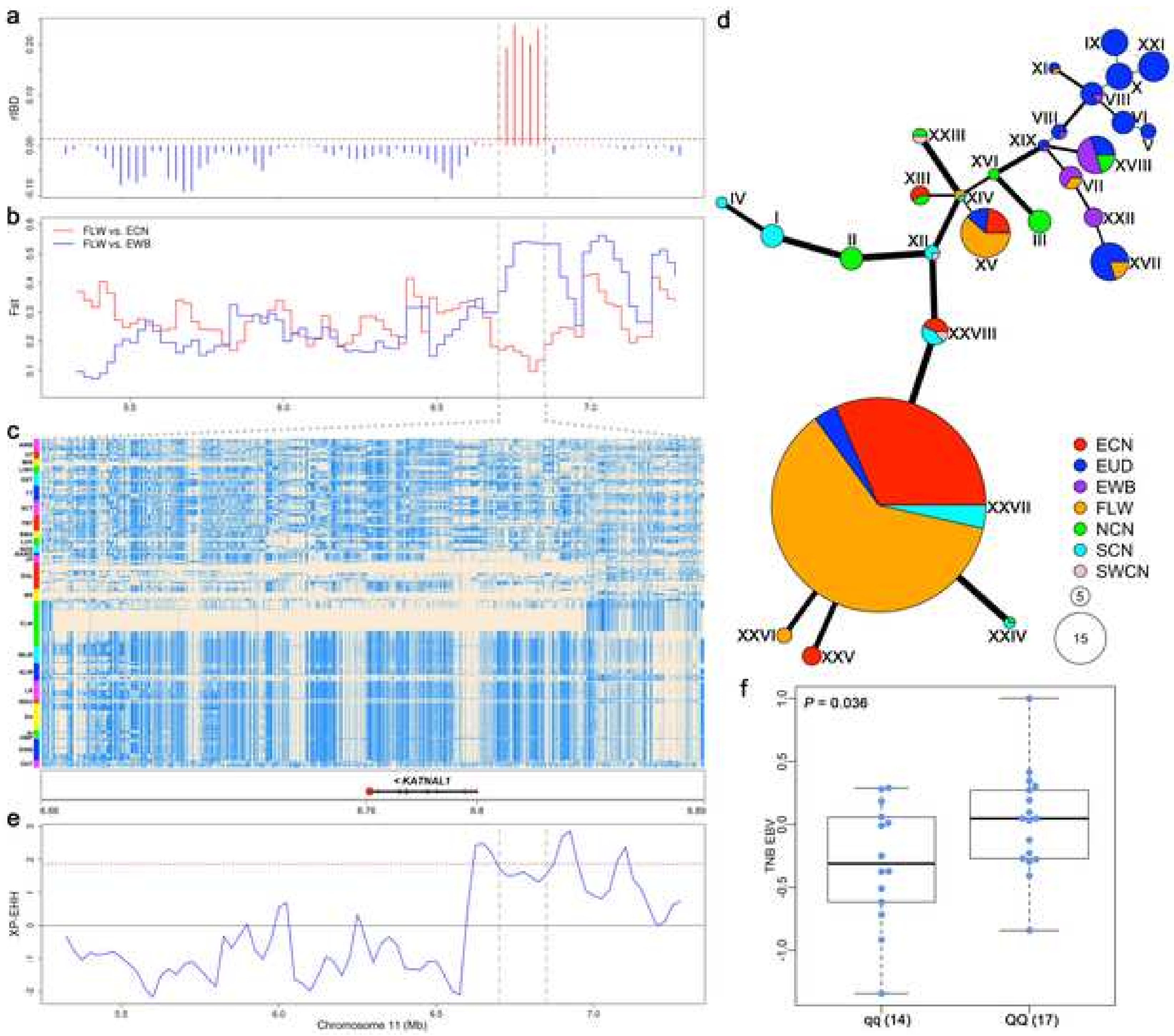

Figure 5

Click here to access/download;Figure;Figure 5.tif

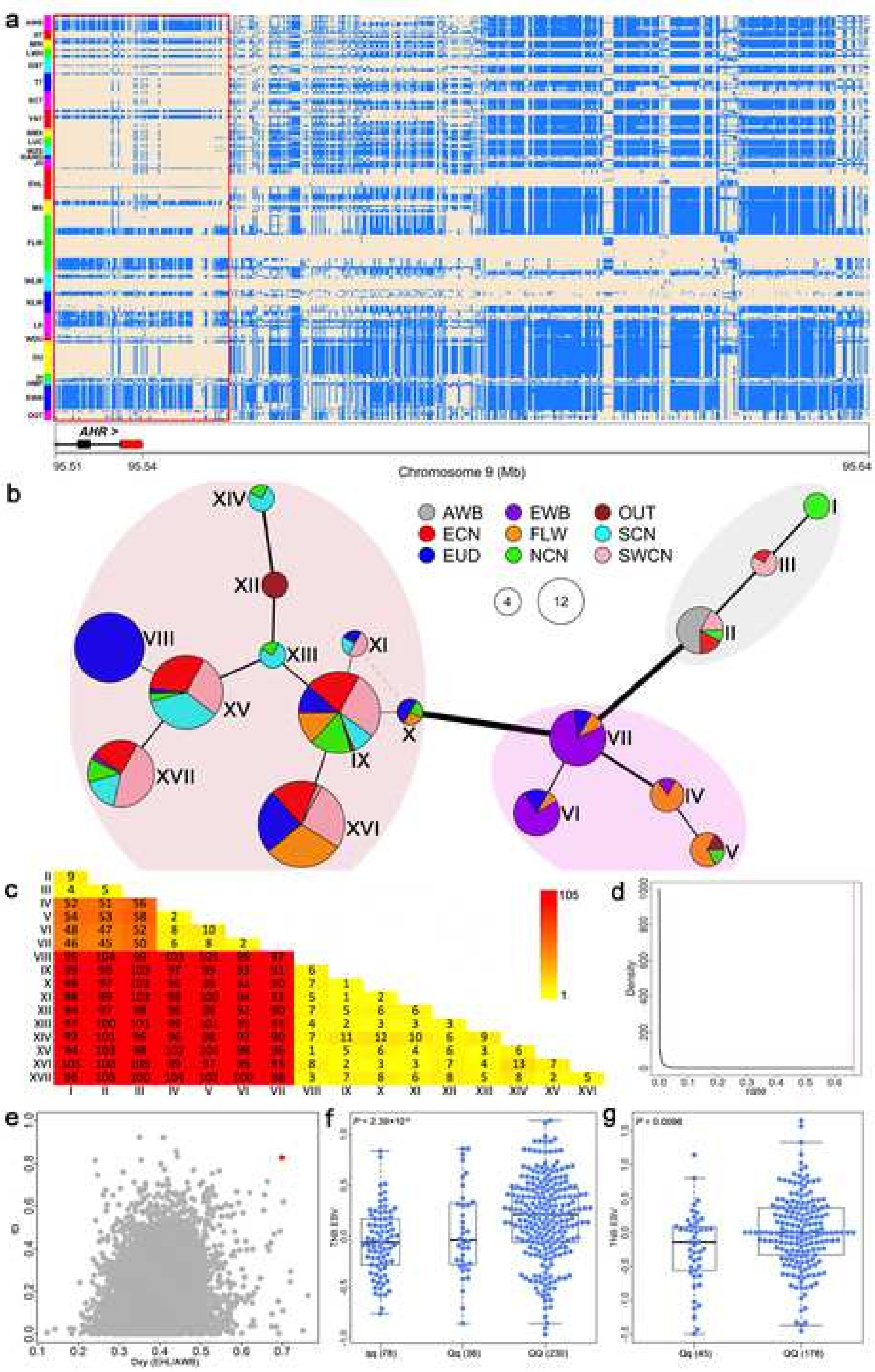

Click here to access/download
Supplementary Material
Supplementary Information.docx

Table S2

Click here to access/download
Supplementary Material
Table S2.xlsx

Table S3

Click here to access/download
Supplementary Material
Table S3.xlsx

Table S6

Click here to access/download
Supplementary Material
Table S6.xlsx

Table S7

Click here to access/download
Supplementary Material
Table S7.xlsx
