# Supplementary material for: Introgression of Eastern Chinese and Southern Chinese haplotypes contributes to the improvement of fertility and immunity in European modern pigs
Source: Gigascience. 2020 Mar 6;9(3):giaa014. doi: 10.1093/gigascience/giaa014 (PMC7059266; doi:10.1093/gigascience/giaa014)
Supplement: giaa014_Supplemental_Figures_and_Tables [file giaa014_supplemental_figures_and_tables.zip › Supplementary Information.docx]

**Supplementary Figures**


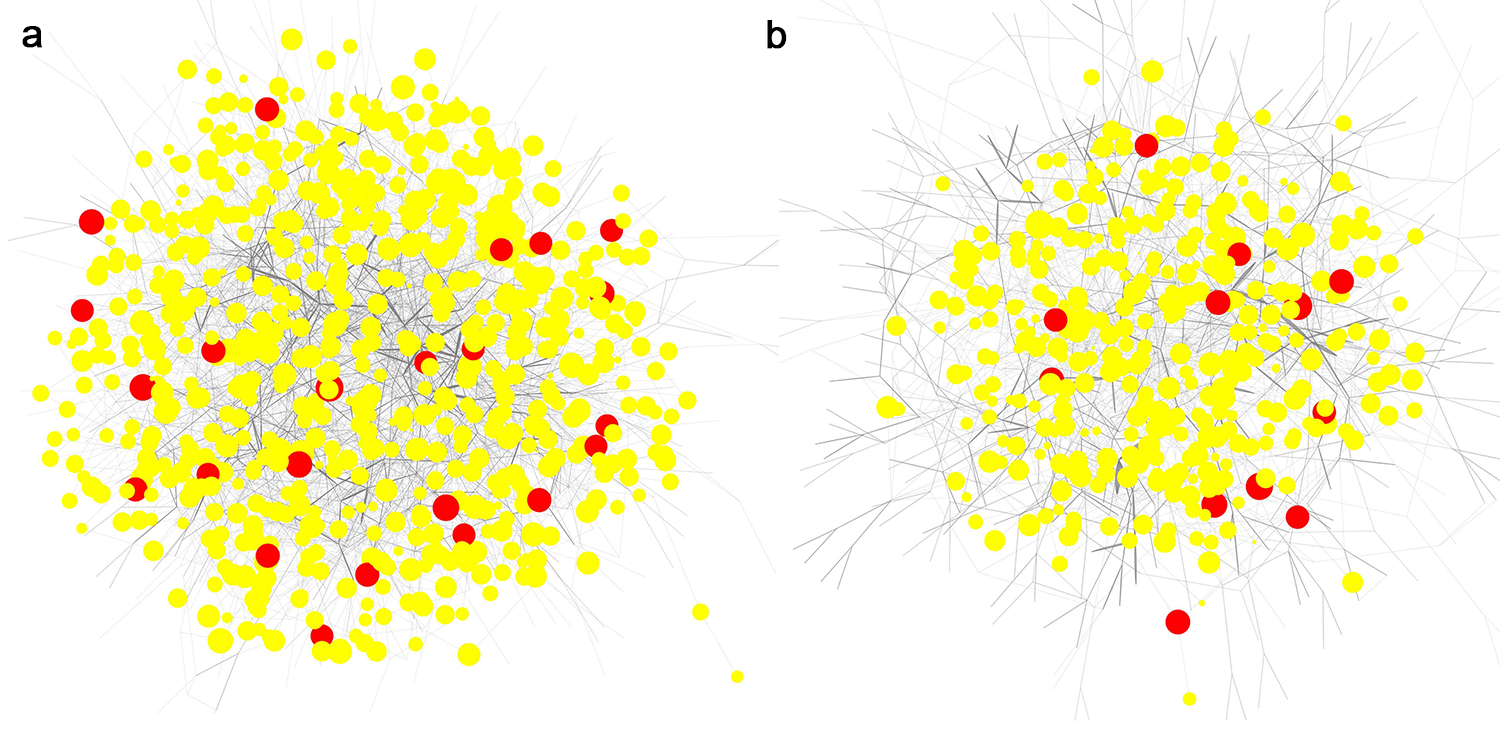


**Supplementary Fig. 1**

**Pedigree based relationship network among individuals constructed by Cytoscape v3.2.1.** (a) Relationship network of French Large White sows from the WENS company in Guangdong Province. Twenty-four resequenced individuals are highlighted by red dots. (b) Relationship network of French Large White sows from the Lvhuan company in Jiangxi Province. Twelve resequenced individuals are highlighted by red dots. Yellow dots indicate unsequenced individuals. Larger dots indicate sows with higher litter size. The longer and thicker line represents more distant relationship.

**
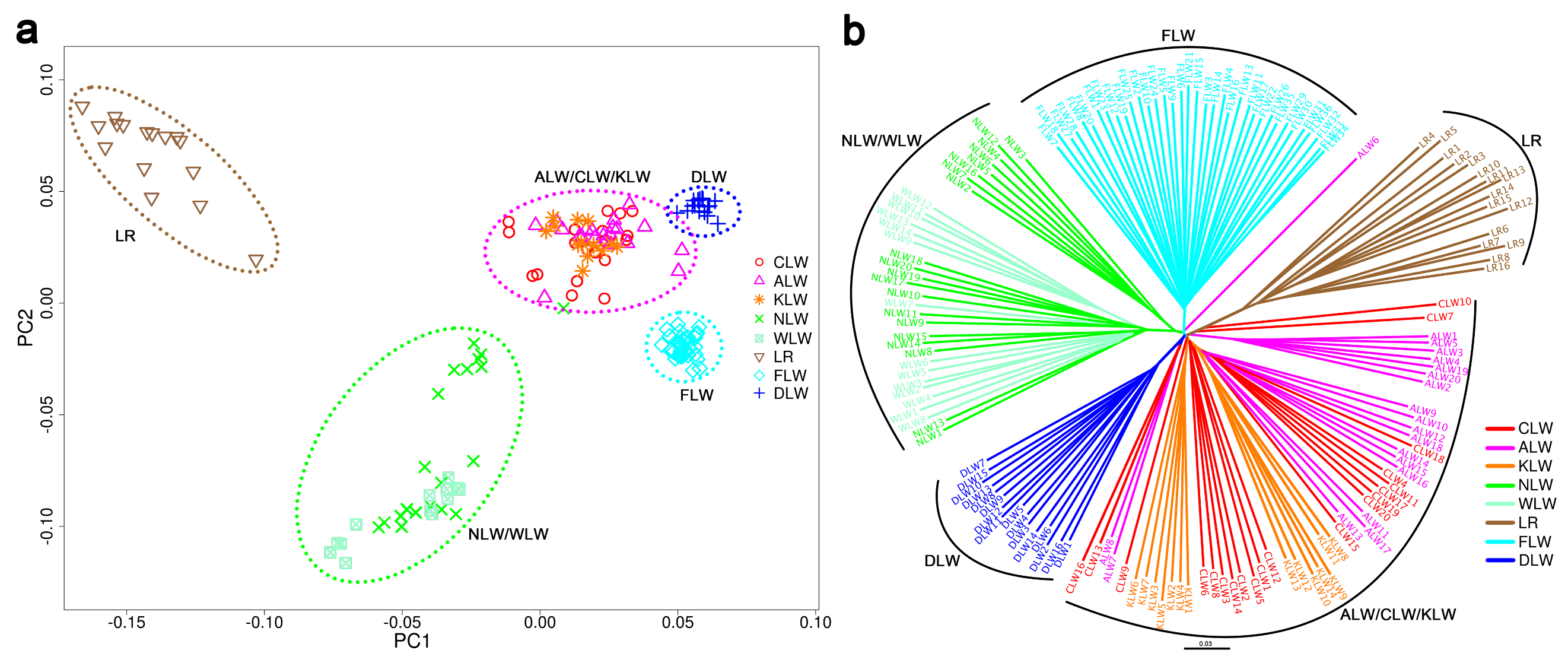
**

**Supplementary Fig. 2**

**Genetic relationships of Large White pigs from different countries.** (a) Principal component analysis. (b) Neighbor-joining clustering tree. CLW, Chinese Large White pigs; ALM, American Large White pigs; KLW, Korea Large White pigs; NLW/WLW, Dutch Large White pigs; FLW, French Large White pigs; DLW, Danish Large White pigs; LR, Landrace.

**
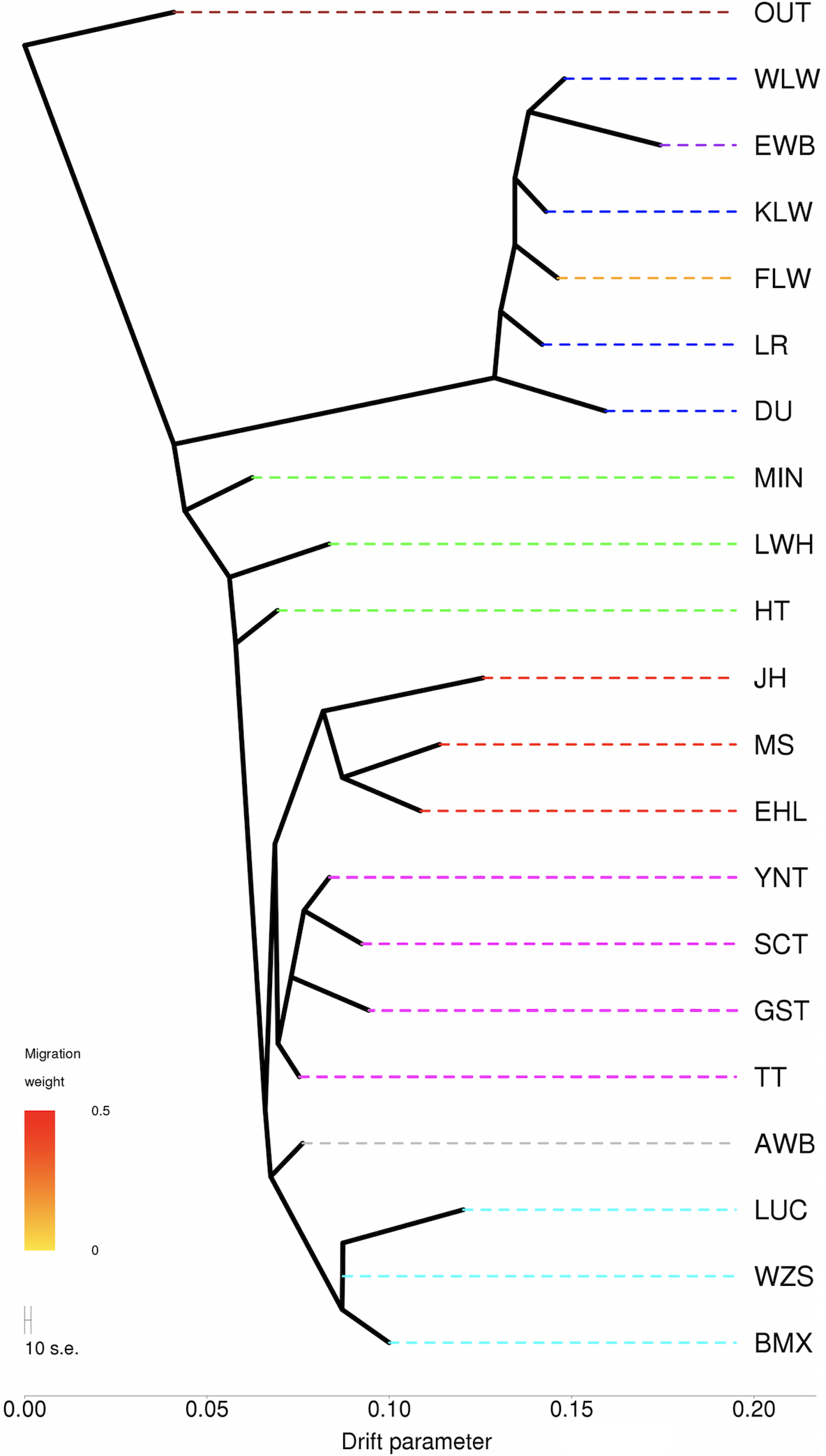
**

**Supplementary Fig. 3**

**Genetic relationships amongst 21 Chinese and Western pig populations inferred using the TreeMix program** **without migration edges**. OUT was used as an outgroup to root the tree. The length of the branch is proportional to the drift of each population. The scale bar shows ten times of average standard error (s.e.) of the entries in the sample covariance matrix. The colored dashed lines in the phylogenetic tree represent different genetic groups. Cyan, red, pink, green, and blue dashed lines represent SCN, ECN, SWCN, NCN and EUD, respectively. The full names of abbreviations of these 21 pig populations are given in the legend of Figure 1.

**
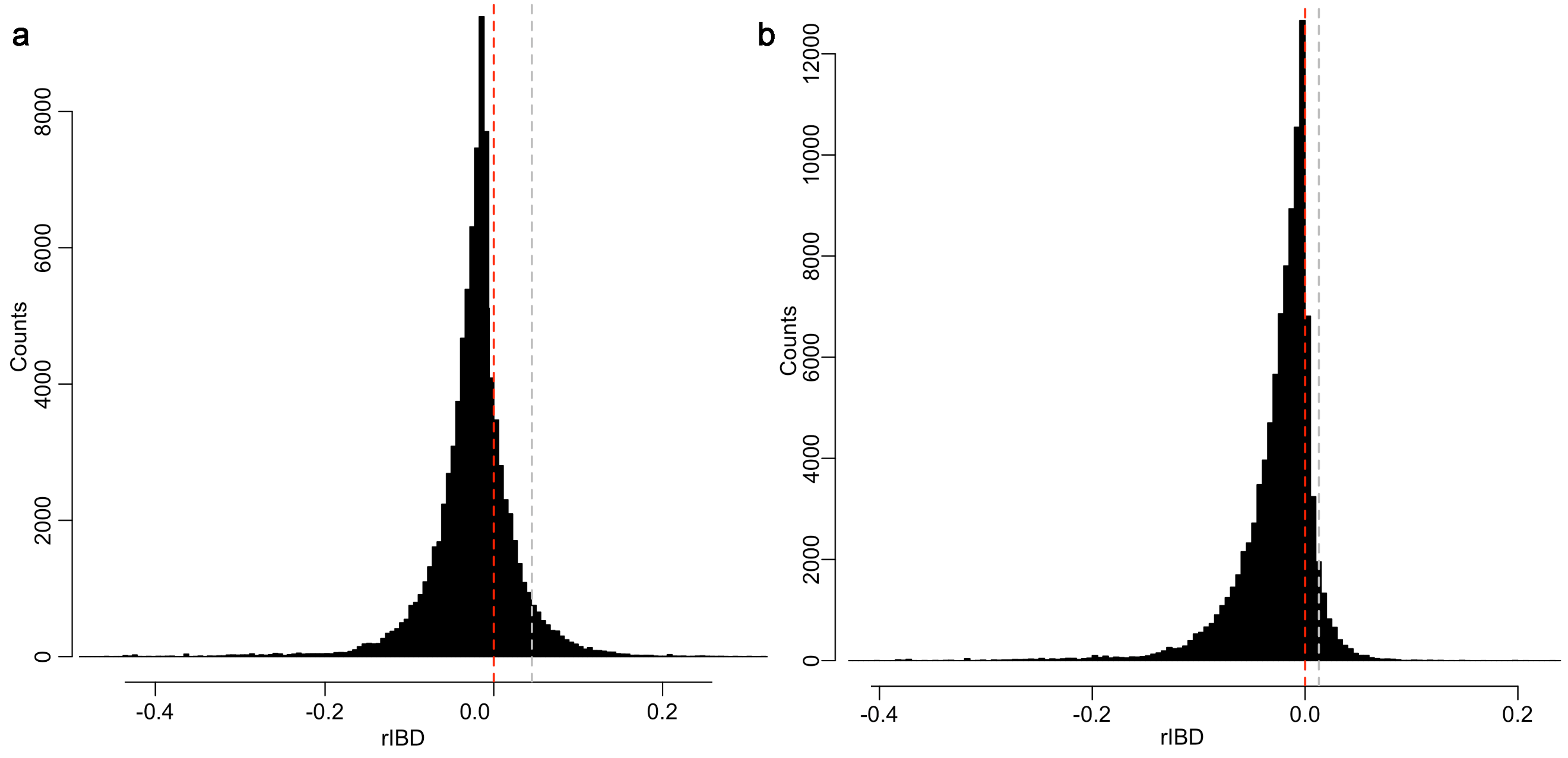
**

**Supplementary Fig. 4**

**Distribution curves of rIBD values.** (a) Distribution curve of rIBD values between FLW and SCN (positive value) or EWB (negative value). The red dash line indicates the top 5% significant threshold; (b) Distribution curve of rIBD values between FLW and ECN (positive value) or EWB (negative value). The red dash line indicates the top 5% significant threshold.


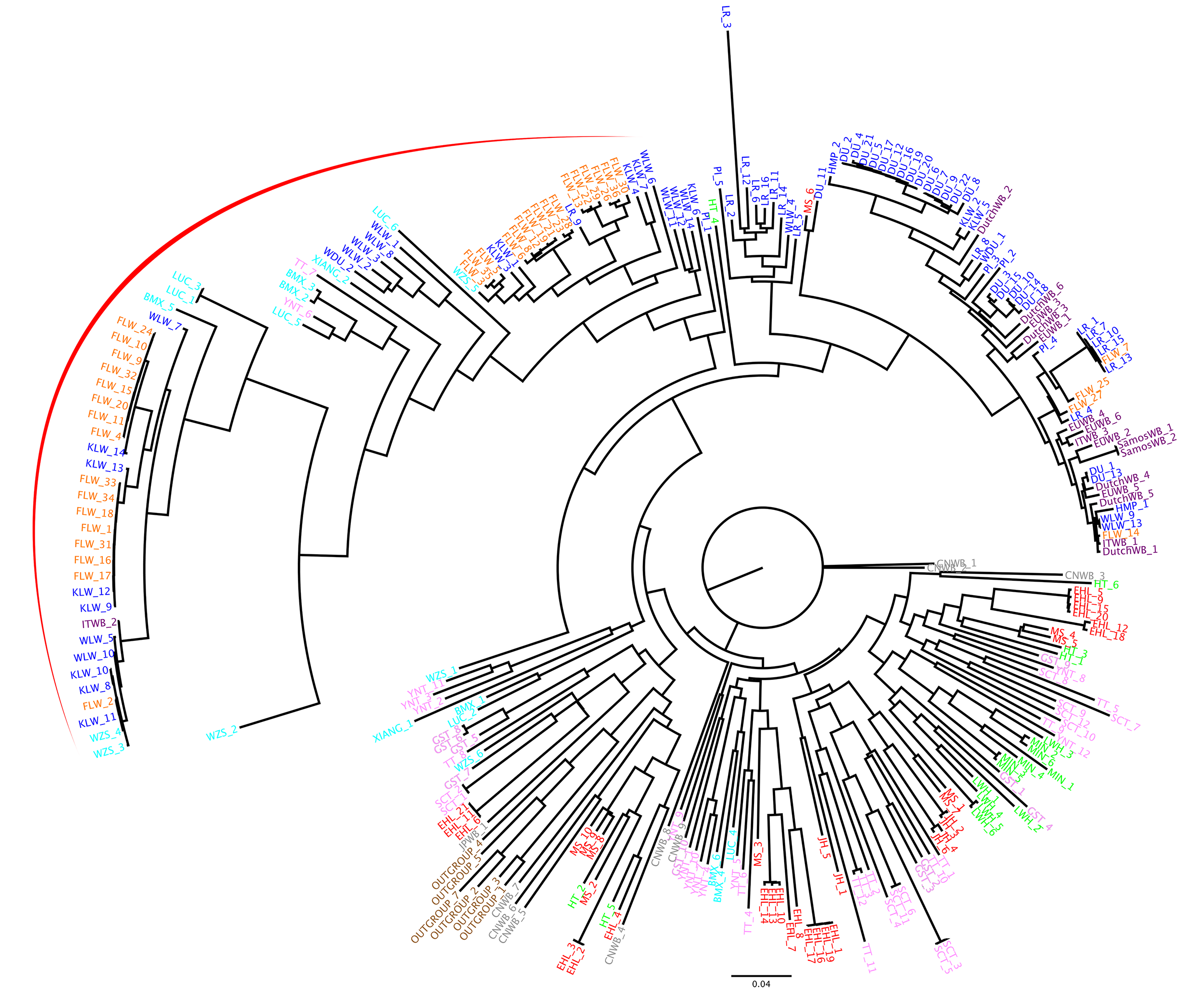


**Supplementary Fig. 5**

**Neighbor joining tree in the *GOLM1-NAA35* region.** The red arc indicates the major clade that include French Large White (FLW) pigs, other Large White pigs, Luchuan (LUC), Wuzhishan (WZS) and Bamaxiang (BMX) pigs from South China. ECN, red texts; NCN, green texts; SCN, cyan texts; SWCN, pink texts; EUD, blue texts; AWB, grey texts; EWB, purple texts; OUT, brown texts; FLW, orange texts.


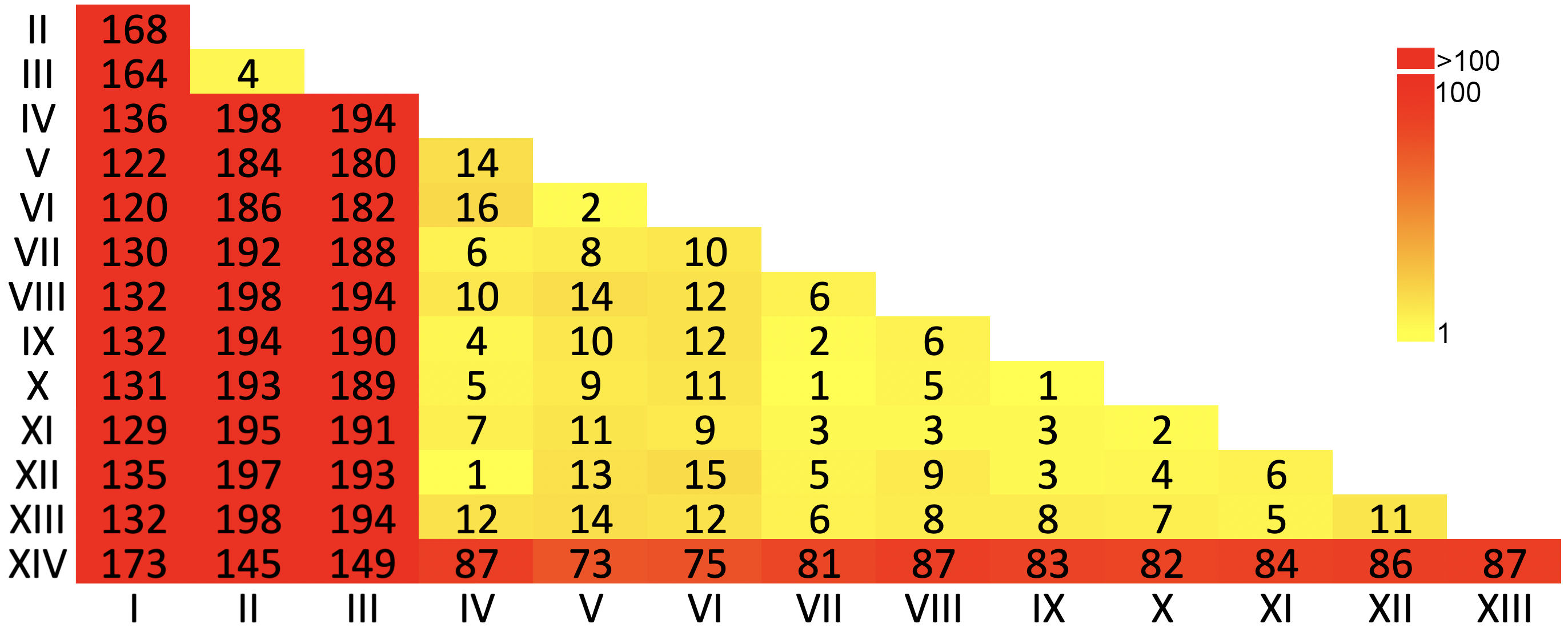


**Supplementary Fig. 6**

**Haplotype difference at the *GOLM1-NAA35* locus.**

**
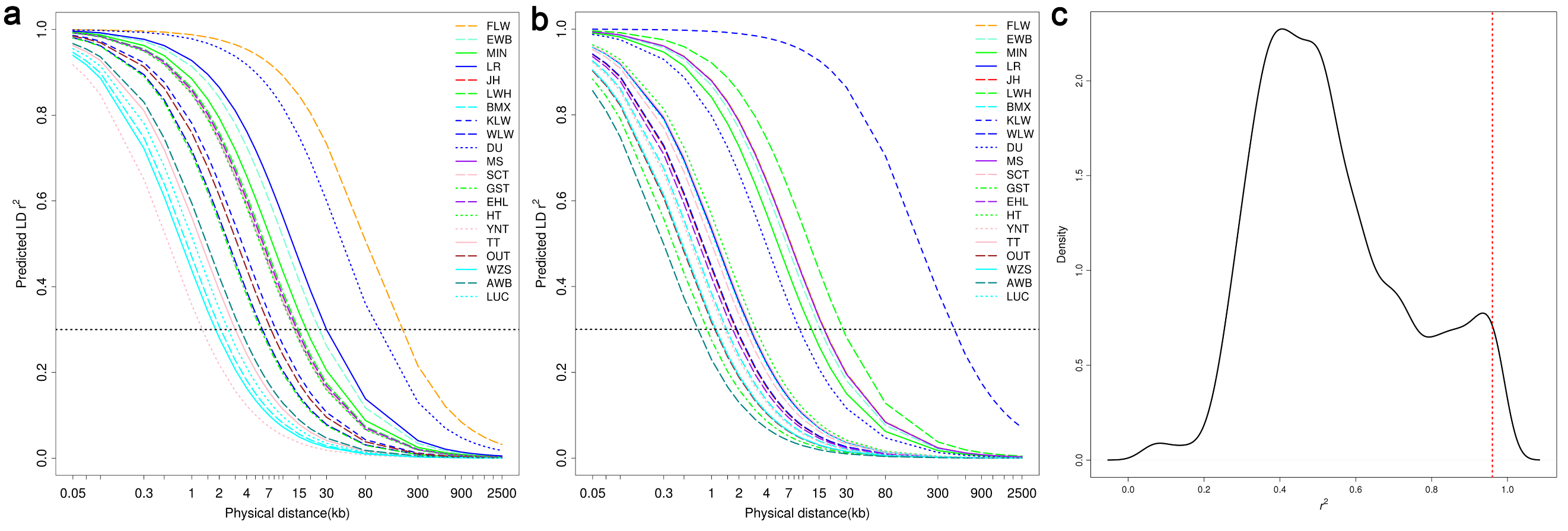
**

**Supplementary Fig. 7**

**Linkage disequilibrium (LD) analysis for *GOLM1-NAA35* haplotypes.** (a) LD decay in the *GOLM1-NAA35* region. LD values were estimated using whole-genome sequence data of six individuals randomly selected from each population. The y-axis indicates the physical distance, the ordinate indicates the predicted LD(*r^2^*) value, and the horizontal dashed line indicates the threshold line (*r^2^* = 0.3); (b) LD decay in a upstream (3 Mb) genomic region of the same size as the *GOLM1-NAA35* region; (c) Density curve of LD (*r^2^*) bootstrap values for 10,000 regions of the same size as the *GOLM1-NAA35* region in French Large White pigs. The red dashed line represents the LD(*r^2^*) value in the *GOLM1-NAA35* region.

**
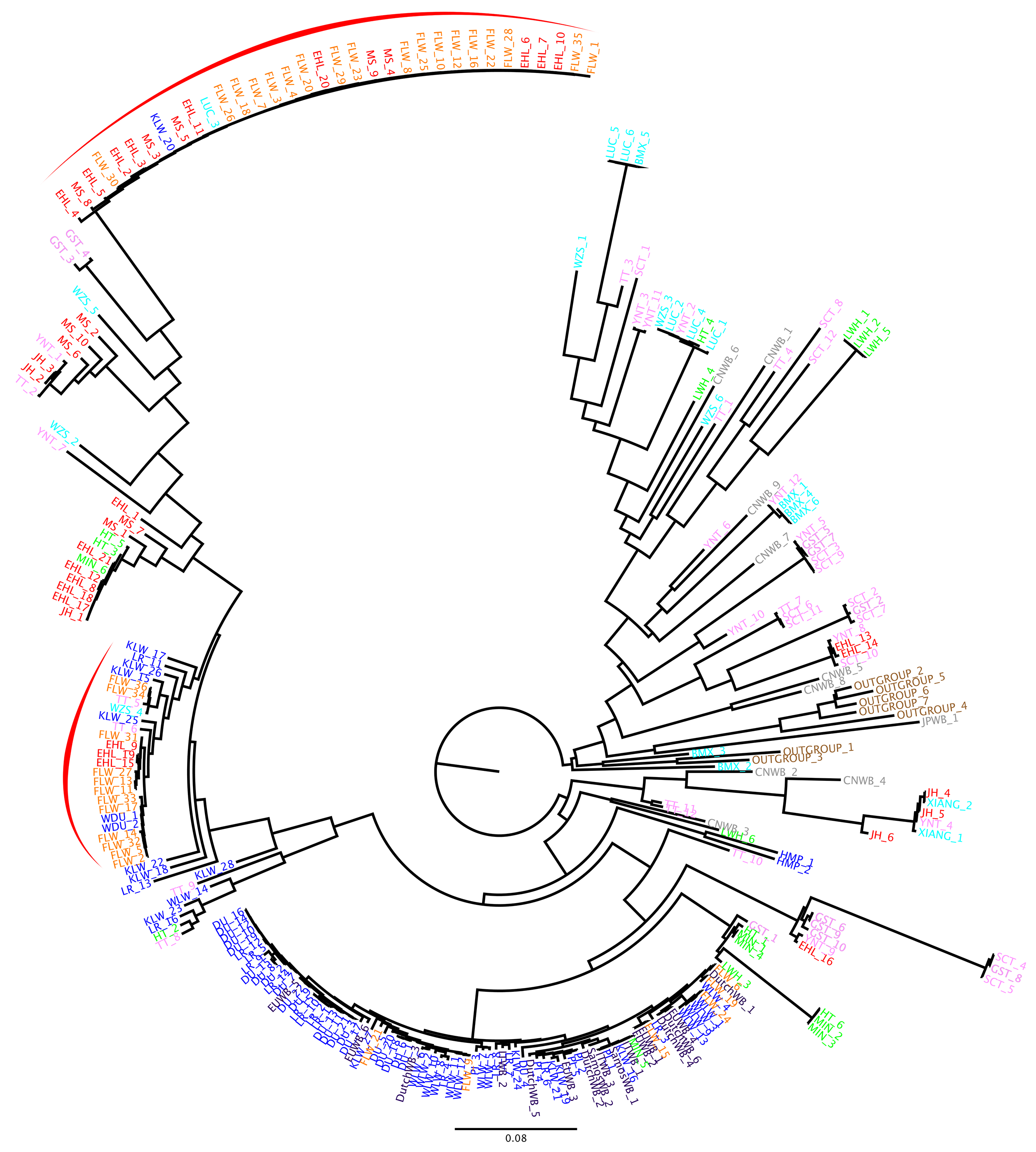
**

**Supplementary Fig. 8**

**Neighbor-joining phylogenetic tree of the tested individuals in the *KATNAL1* region.** The red arc represents the major clade that include French Large White (FLW) pigs, other Large White pigs, Erhualian (EHL) and Meishan (MS) pigs from East China. ECN, red texts; NCN, green texts; SCN, cyan texts; SWCN, pink texts; EUD, blue texts; AWB, grey texts; EWB, purple texts; OUT, brown texts; FLW, orange texts.

**
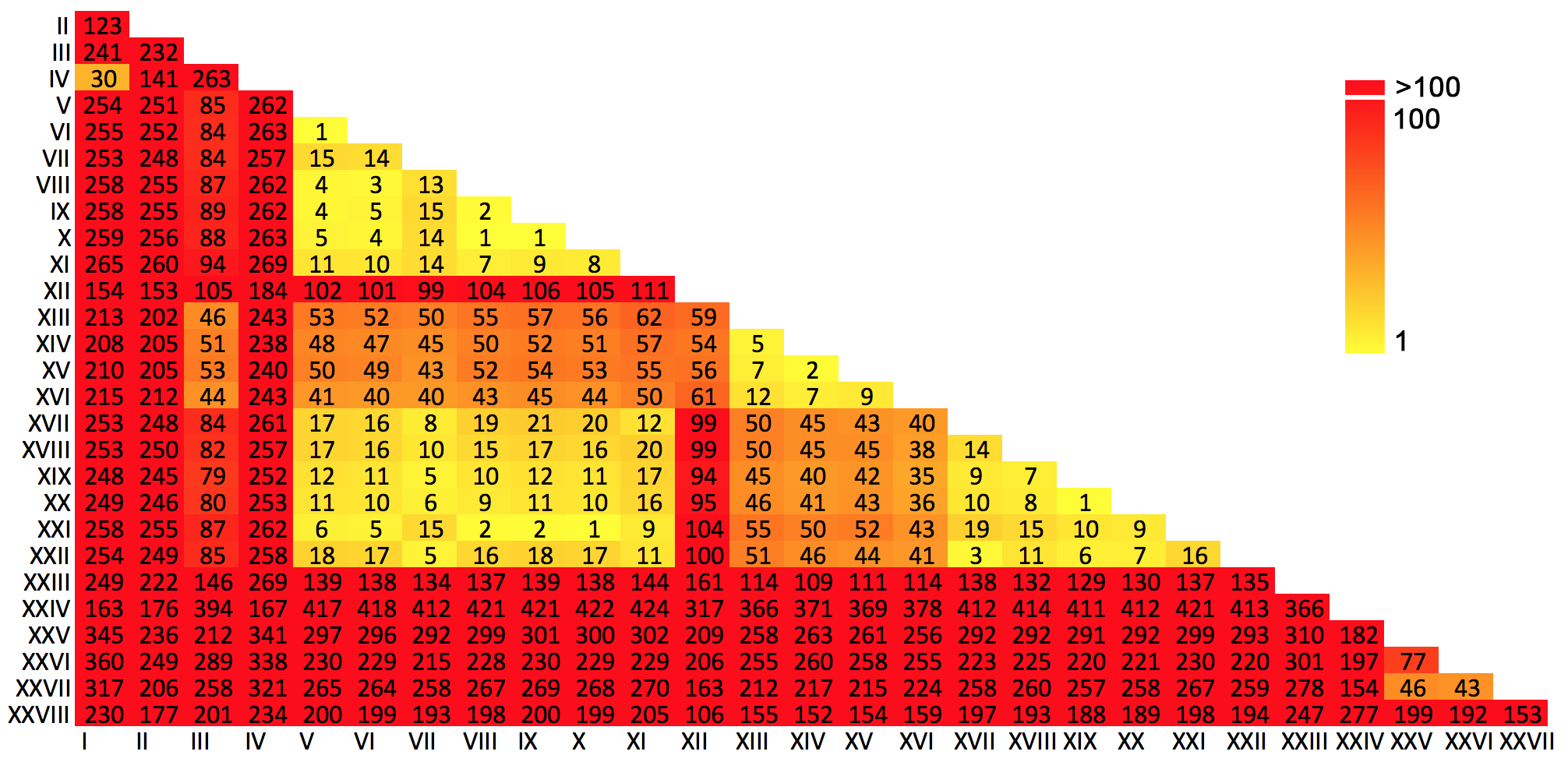
**

**Supplementary Fig. 9**

**Haplotype difference in the *KATNAL1* gene.**

**
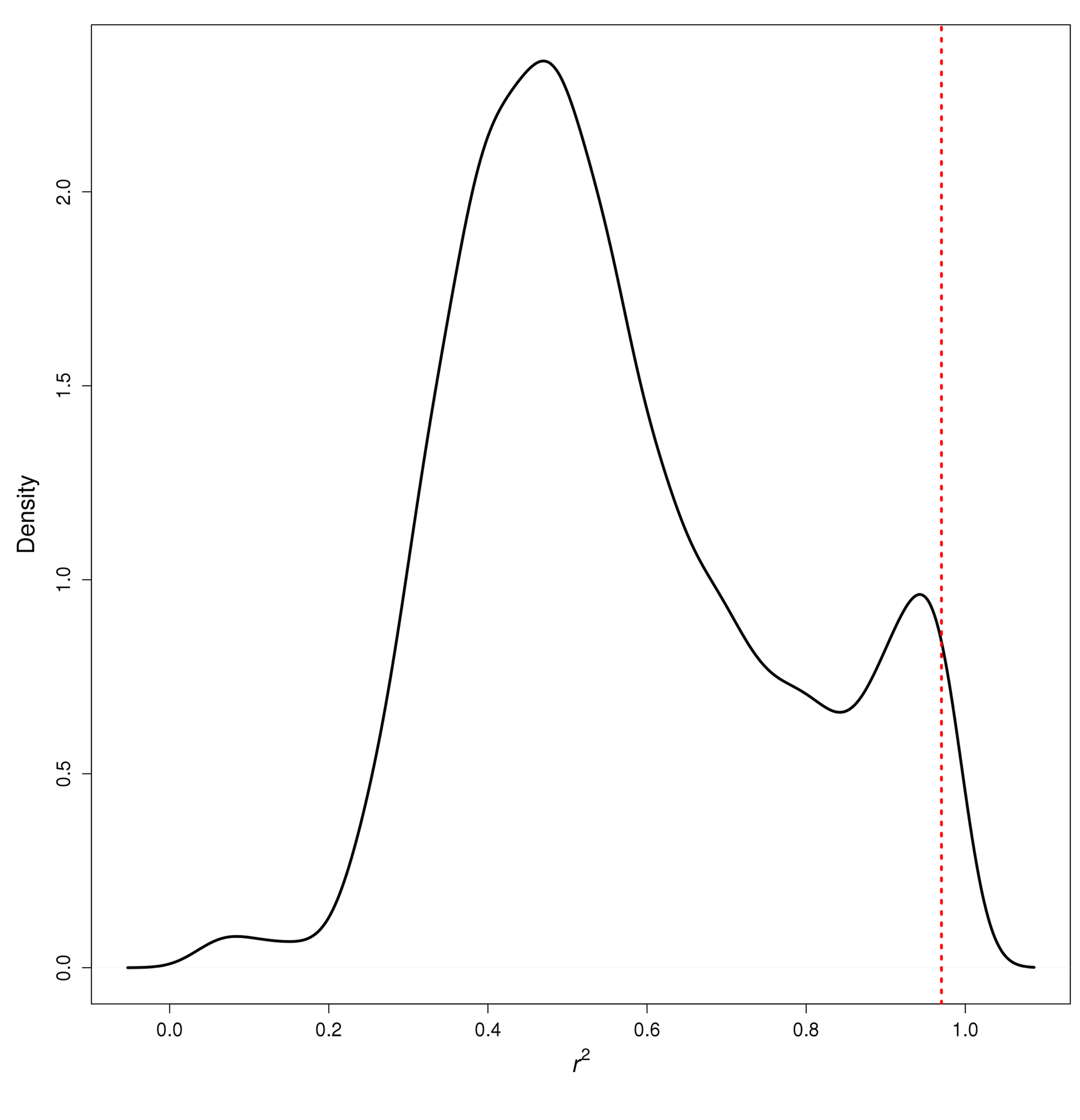
**

**Supplementary Fig. 10**

**Linkage disequilibrium (LD) at the *KATANL1* locus in French Large White pigs.** This figure shows the density curve of LD (*r^2^*) bootstrap values for 10,000 regions with the same size as *KATNAL1* that were randomly selected across the whole genome in French Large White pigs. The red dashed line represents the LD (*r^2^*) value of the *KATNAL1* gene.

**
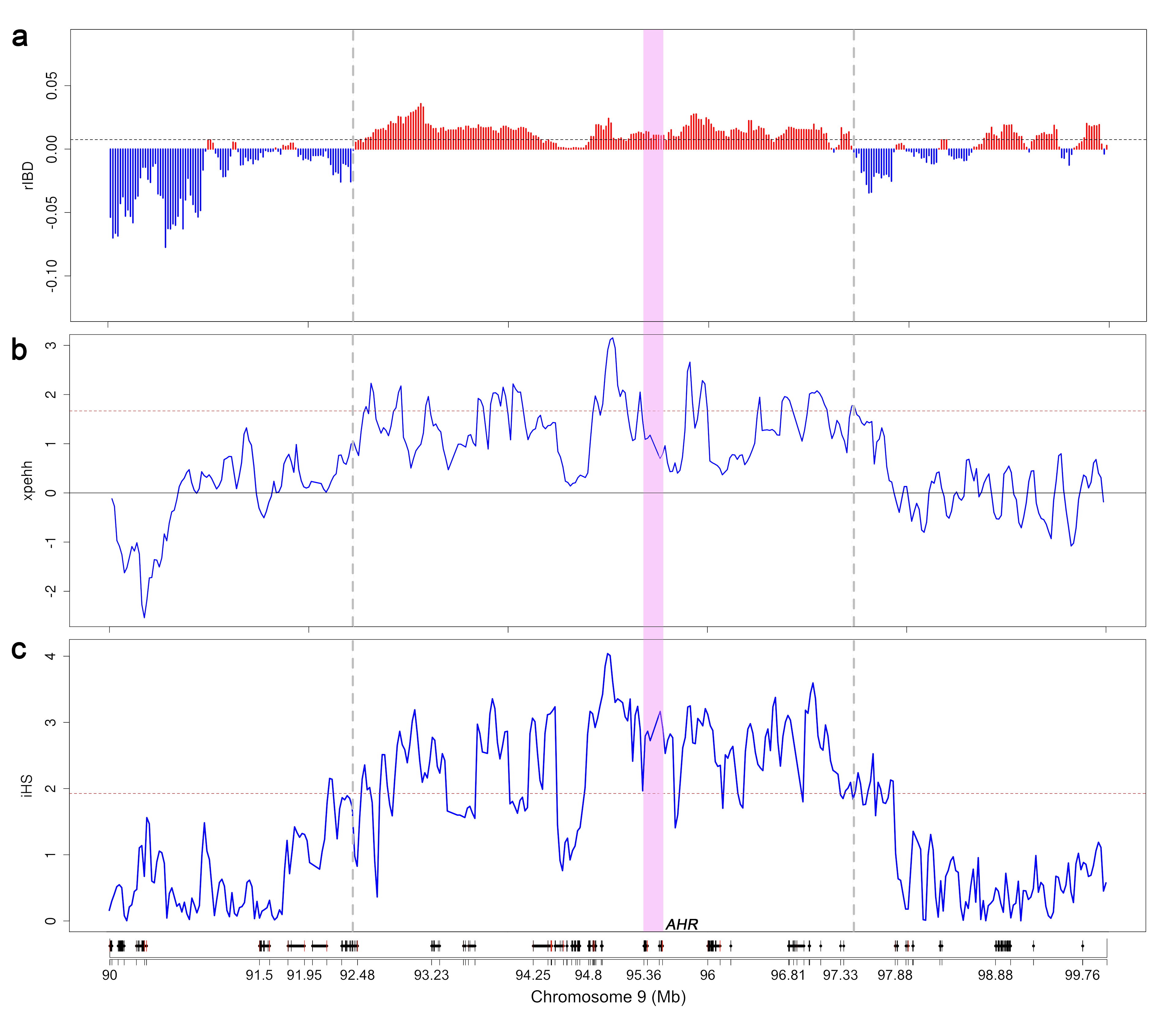
**

**Supplementary Fig. 11**

**rIBD and selection signals around the *AHR* region in Large White pigs.** (a) rIBD between Large White pigs and Chinese domesticated pigs in the SSC9: 90-100 Mb region (Sscrofa10.2, 81.8-90.6 Mb on Sscrofa11.1). The brown dotted line indicates the 5% threshold line. (b) Selection signals detected by the XP-EHH analysis between Large White pigs and European wild boars. (c) Selection signals within Large White pigs revealed by the iHS analysis. The brown dashed line represents the genome wide 5% threshold line, and the introgression region is indicated by two grey dashed lines. The pink shaded area represents the *AHR* gene region.

**
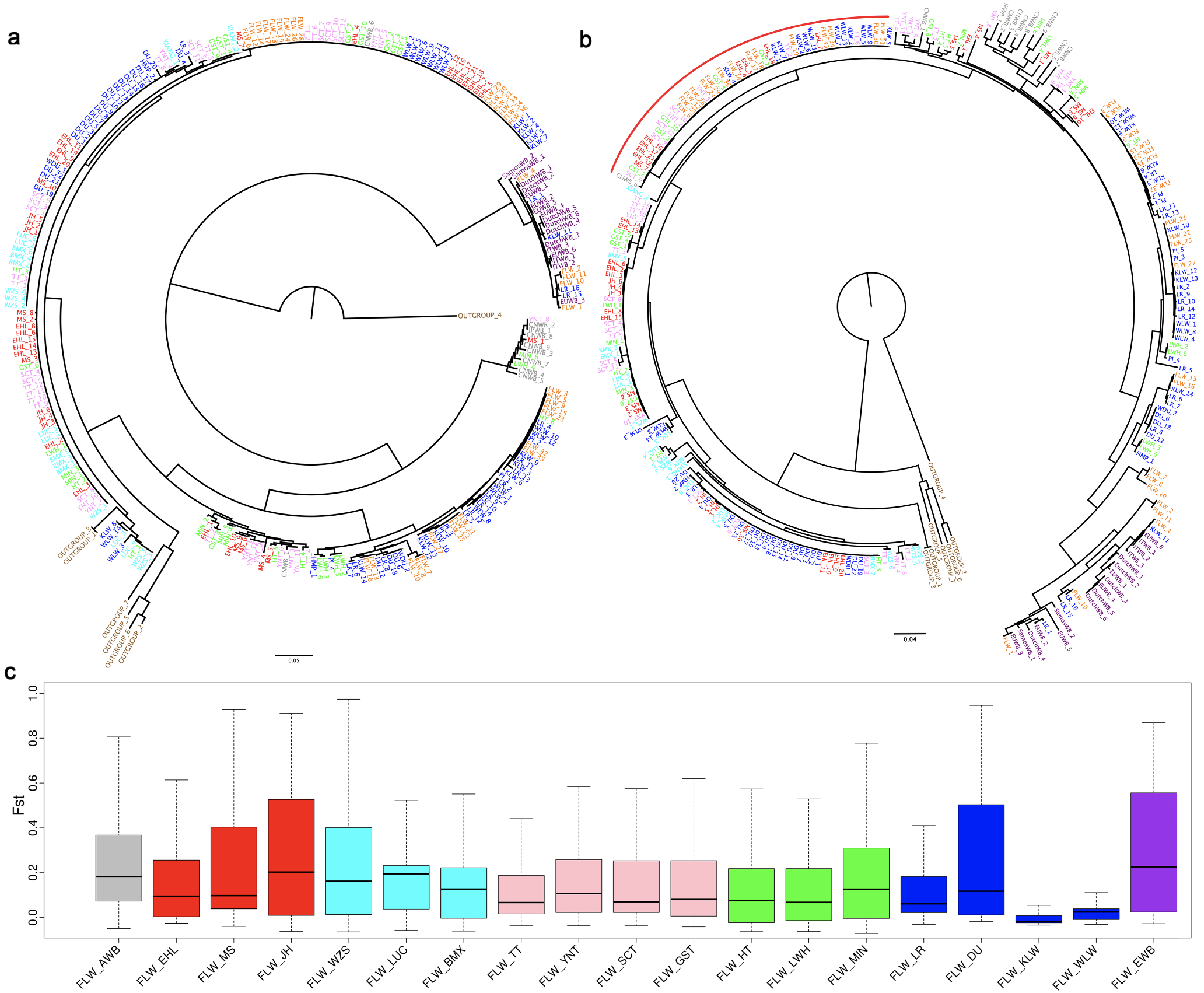
**

**Supplementary Fig. 12**

**Genetic relationships between French Large White pigs and other pig breeds in the *AHR* region.** (a) Neighbor-joining tree in the *AHR* gene. (b) Neighbor-joining tree in the SSC9: 86.5-86.6 Mb (Sscrofa11.1, 95.4-95.6 Mb on Sscrofa10.2) region encompassing the *AHR* gene. The red arc represents the major clade of French Large White (FLW) pigs. (c) Box plot of genetic differentiation between FLW pigs and other pig breeds in the *AHR* region (SSC9: 86.5-86.6 Mb). Different colors represent pig breeds from different geographical regions. Grey represents the genetic differentiation index between FLW pigs and Asian wild boars (AWB). Red, cyan, pink, green, blue and purple boxes represent the genetic differentiation index (F_ST_) between FLW and ECN, SCN, SWCN, NCN, EUD, and EWB, respectively.

**
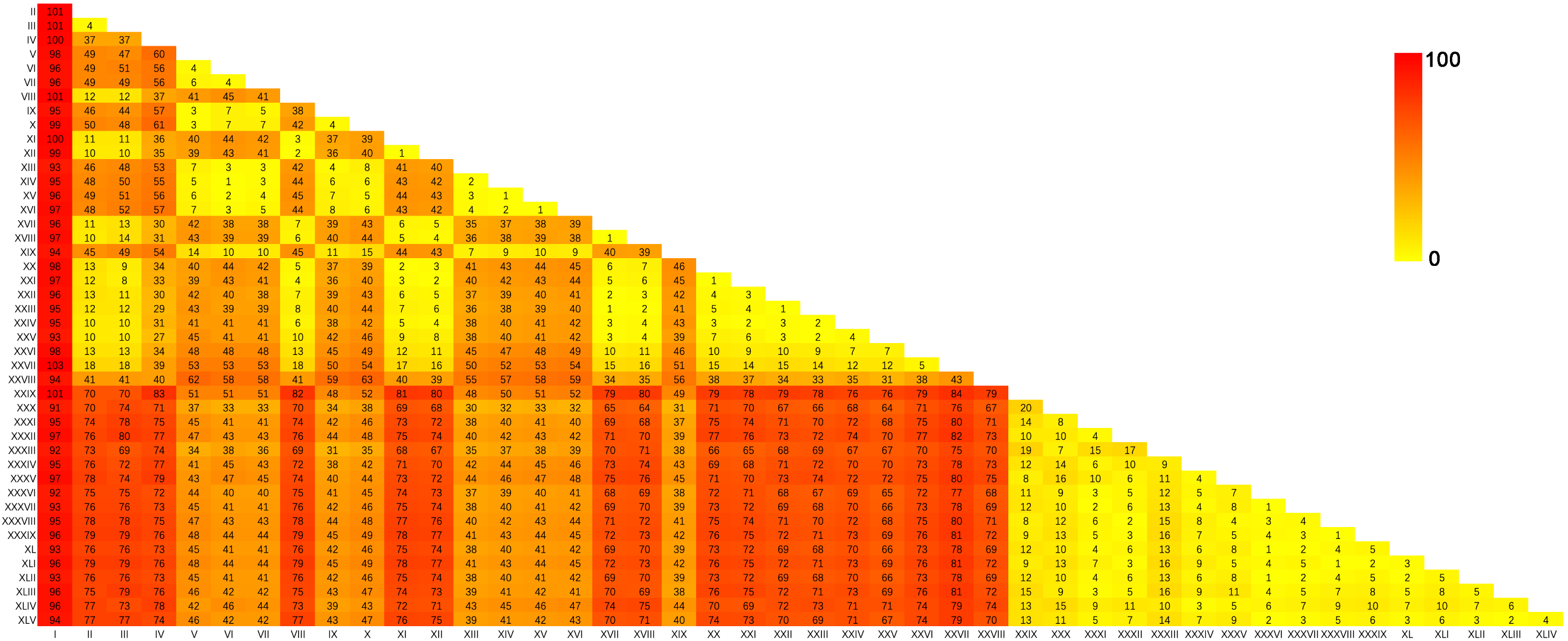
**

**Supplementary Fig. 13**

**Haplotype difference at the *AHR* locus.**

**
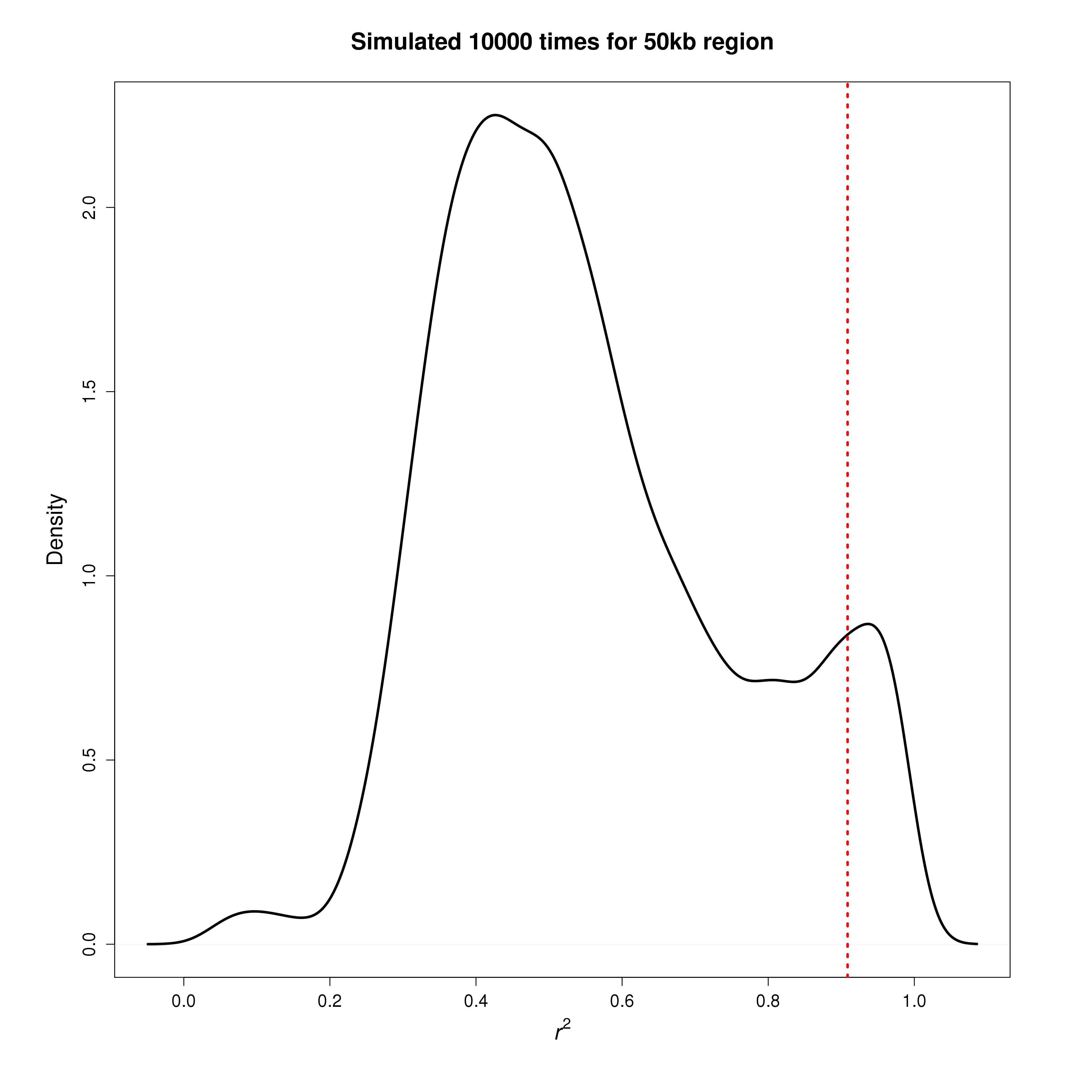
**

**Supplementary Fig. 14**

**Linkage disequilibrium (LD) at the *AHR* locus in French Large White pigs.** This figure shows the density curve of LD (*r^2^*) bootstrap values for 10,000 randomly selected regions with the same size as the *AHR* gene in French Large White pigs. The red dashed line represents the LD (*r^2^*) value in the *AHR* gene.

**
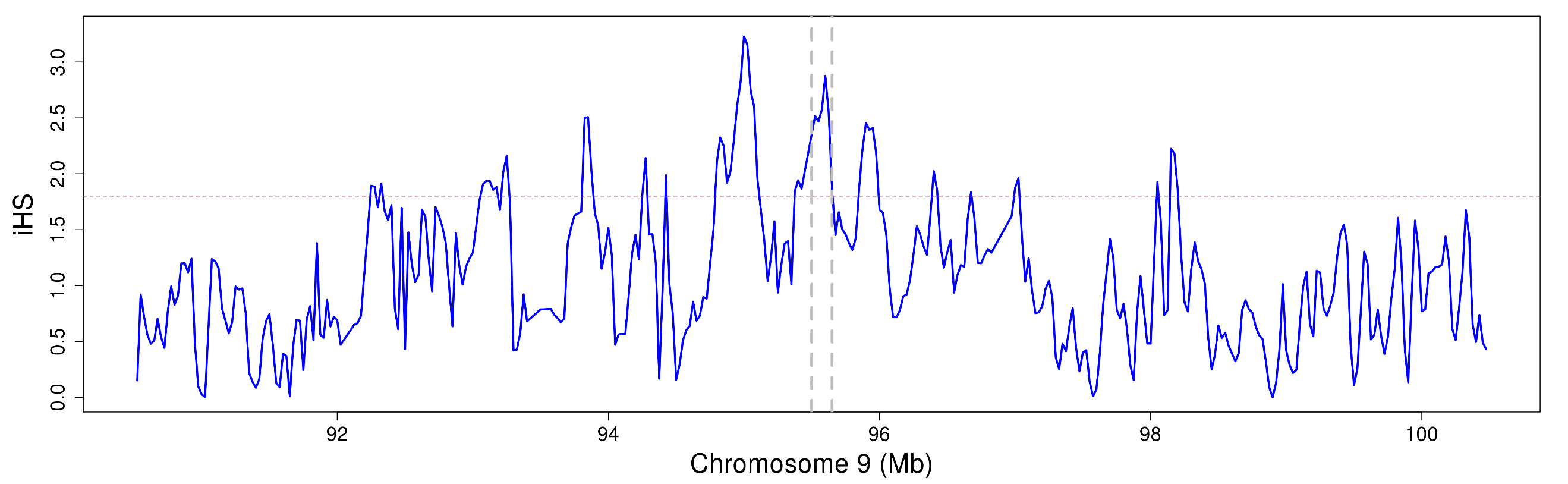
**

**Supplementary Fig. 15**

**Selection signals in the *AHR* region in French Large White pigs.** The signals were detected by the iHS analysis. The *AHR* gene region is indicated by two vertical dashed lines.

**Supplementary Tables**

**Supplementary Table 1. Animals and their whole genome sequencing information**

| **Classification** | **Population** | **Abbreviation** | **No.** | **Depth (×)** |
| --- | --- | --- | --- | --- |
| Asian Wild Boar | Asian Wild Boar | AWB | 6 | 23.42 |
|  | Asian Wild Boar^1^ | AWB | 4 | 19.06 |
| East Chinese (ECN) pigs | Erhualian | EHL | 21 | 28.17 |
|  | Jinhua | JH | 6 | 26.24 |
|  | Meishan^1^ | MS | 10 | 10.36 |
| South Chinese (SCN) pigs | Bama Xiang | BMX | 6 | 27.13 |
|  | Luchuan | LUC | 6 | 26.37 |
|  | Wuzhishan | WZS | 6 | 26.09 |
|  | Xiang^1^ | XIANG | 2 | 9.05 |
| Southwest Chinese (SWCN) pigs | Sichuan Tibetan | SCT | 12 | 26.78 |
|  | Xizang Tibetan | TT | 12 | 25.95 |
|  | Yunnan Tibetan | YNT | 12 | 26.41 |
| North Chinese (NCN) pigs | Gansu Tibetan | GST | 10 | 26.66 |
|  | Laiwu | LWH | 6 | 26.14 |
|  | Min | MIN | 6 | 25.80 |
|  | Hetao | HT | 6 | 24.40 |
| European Wild Boar | European Wild Boar^1^ | EWB | 17 | 12.90 |
| European Domestic (EUD) pigs | French Large White | FLW | 36 | 22.03 |
|  | White Duroc | WDU | 2 | 36.81 |
|  | Duroc^1^ | DU | 22 | 13.96 |
|  | Korea Large White^1^ | KLW | 14 | 16.18 |
|  | Dutch Large White^1^ | WLW | 14 | 9.40 |
|  | Hampshire^1^ | HMP | 2 | 10.81 |
|  | Landrance^1^ | LR | 16 | 12.14 |
|  | Pietrain^1^ | PI | 5 | 9.40 |
| Outgroup | *Sus scrofa (Sumatra)*^1^ | OUT | 2 | 11.13 |
|  | *Sus barbatus*^1^ | OUT | 1 | 7.24 |
|  | *Sus verrucosus*^1^ | OUT | 1 | 12.75 |
|  | *Sus cebifrons*^1^ | OUT | 1 | 8.43 |
|  | *Sus celebensis*^1^ | OUT | 1 | 23.89 |
|  | *Phacochoerus africanus*^1^ | OUT | 1 | 13.12 |
| Total |  |  | 266 |  |

^1^ Whole genome sequence data of these populations were downloaded from the public NCBI database with accession numbers ERP001813, PRJEB9922 and SRP047260.

**Supplementary Table 2. The 50 kb regions of potential introgression from South China pigs into French Large White pigs (see excel file “Table S2”)**

**Supplementary Table 3. The 50 kb regions of potential introgression from East China pigs into French Large White pigs (see excel file “Table S3”)**

**Supplementary Table 4.** **Strong candidate regions encompassing introgressed Chinese haplotypes in the genomes of French Large White pigs**

| **Origin** | **CHR** | **Region (Mb)** | **Length (Kb)** | **TopWin (Mb)** | **Top_rIBD** | **Pvalue** | **Gene** |
| --- | --- | --- | --- | --- | --- | --- | --- |
| SCN | 1 | 167.125-167.55 | 425 | 167.25-167.3 | 0.26 | 5.75E-09 | *CBLN2* |
|  | 2 | 123.45-124.125 | 675 | 123.65-123.7 | 0.31 | 2.29E-11 | *KCNN2* |
|  | 3 | 112.18-112.675 | 495 | 112.525-112.575 | 0.28 | 1.25E-09 | - |
|  | 10 | 33.2-33.575 | 375 | 33.4-33.45 | 0.26 | 1.64E-08 | *GOLM1*  *NAA35* |
|  | 13 | 17.2-17.4 | 200 | 17.275-17.325 | 0.28 | 7.39E-10 | - |
|  | 16 | 31.325-31.625 | 300 | 31.45-31.5 | 0.37 | 9.92E-16 | *ATP5H* |
|  | 16 | 76.95-77.15 | 200 | 77.05-77.1 | 0.36 | 2.44E-15 | - |
| ECN | 1 | 232.4-233.325 | 925 | 233.125-233.175 | 0.23 | 4.81E-11 | *MPDZ* |
|  | 11 | 6.675-6.875 | 200 | 6.725-6.775 | 0.24 | 9.92E-12 | *KATNAL1* |
|  | 16 | 2.175-2.3 | 125 | 2.225-2.275 | 0.21 | 2.23E-09 | - |

**Supplementary Table 5. *GOLM1*-*NAA35* haplotypes and serum interleukin 6 concentrations of 54 French Large White sows**

| **ID** | **SNP1** | **SNP2** | **Genotype** | **IL-6 (pg/ml)** | **SD** |
| --- | --- | --- | --- | --- | --- |
| YYJXLH115191906 | GG | GG | QQ | 11.027 | 0.0214 |
| YYJXLH115185909 | GG | GG | QQ | 7.117 | 0.0301 |
| YYJXLH115194203 | AG | CG | Qq | 5.382 | 0.0264 |
| YYJXLH115147104 | AG | CG | Qq | 5.301 | 0.0262 |
| YYJXLH115192407 | GG | GG | QQ | 7.188 | 0.0162 |
| YYJXLH115144106 | GG | GG | QQ | 6.330 | 0.0599 |
| YYJXLH115184905 | GG | GG | QQ | 10.625 | 0.0063 |
| YYJXLH115192408 | GG | GG | QQ | 7.135 | 0.0104 |
| YYJXLH115185910 | GG | GG | QQ | 5.868 | 0.0098 |
| YYJXLH115008904 | AG | CG | Qq | 5.150 | 0.0208 |
| YYJXLH115002002 | AG | CG | Qq | 5.694 | 0.0058 |
| YYJXLH115150504 | GG | GG | QQ | 8.297 | 0.0508 |
| YYJXLH115136602 | AG | CG | Qq | 5.019 | 0.0081 |
| YYJXLH115137107 | AG | CG | Qq | 8.317 | 0.0256 |
| YYJXLH115145709 | AG | CG | Qq | 5.592 | 0.0096 |
| YYJXLH117041604 | AG | CG | Qq | 4.894 | 0.0102 |
| YYJXLH317006207 | AA | CC | qq | 6.434 | 0.0302 |
| YYJXLH116104302 | GG | GG | QQ | 11.383 | 0.0344 |
| YYJXLH117053406 | GG | GG | QQ | 8.445 | 0.0541 |
| YYJXLH117051902 | AG | CG | Qq | 5.672 | 0.0105 |
| YYJXLH116135601 | AG | CG | Qq | 5.946 | 0.0282 |
| YYJXLH317009709 | GG | GG | QQ | 9.305 | 0.0168 |
| YYJXLH116070106 | AG | CG | Qq | 6.321 | 0.0204 |
| YYJXLH117035208 | AG | CG | Qq | 4.906 | 0.0161 |
| YYJXLH117045804 | AG | CG | Qq | 4.014 | 0.0168 |
| YYJXLH116035501 | AG | CG | Qq | 5.756 | 0.0195 |
| YYJXLH116025508 | AG | CG | Qq | 6.134 | 0.0136 |
| YYJXLH117059608 | GG | GG | QQ | 6.089 | 0.0090 |
| YYJXLH317009407 | GG | GG | QQ | 5.882 | 0.0108 |
| YYJXLH117049507 | AG | CG | Qq | 8.434 | 0.0245 |
| YYJXLH117042004 | AG | CG | Qq | 4.342 | 0.0188 |
| YYJXLH116131207 | GG | GG | QQ | 6.981 | 0.0199 |
| YYJXLH115138807 | GG | GG | QQ | 6.622 | 0.0375 |
| YYJXLH116028402 | AG | CG | Qq | 5.088 | 0.0060 |
| YYJXLH117055903 | AG | CG | Qq | 6.053 | 0.0146 |
| YYJXLH116132602 | GG | GG | QQ | 7.185 | 0.0393 |
| YYJXLH117029207 | GG | GG | QQ | 6.172 | 0.0295 |
| YYJXLH116029102 | AG | CG | Qq | 9.653 | 0.0177 |
| YYJXLH116130303 | AG | CG | Qq | 5.853 | 0.0157 |
| YYJXLH116031402 | GG | GG | QQ | 7.576 | 0.0222 |
| YYJXLH117054911 | GG | GG | QQ | 6.891 | 0.0214 |
| YYJXLH117032302 | AG | CG | Qq | 6.100 | 0.0104 |
| YYJXLH116025506 | AG | CG | Qq | 6.898 | 0.0085 |
| YYJXLH115198303 | GG | GG | QQ | 4.300 | 0.0181 |
| YYJXLH117050304 | GG | GG | QQ | 4.357 | 0.0054 |
| YYJXLH115145805 | GG | GG | QQ | 5.093 | 0.0134 |
| YYJXLH116137202 | AG | CG | Qq | 6.517 | 0.0033 |
| YYJXLH116033601 | GG | GG | QQ | 5.720 | 0.0109 |
| YYJXLH116137201 | AG | CC | qq | 5.231 | 0.0181 |
| YYJXLH317006901 | AG | CG | Qq | 3.969 | 0.0315 |
| YYJXLH117049302 | AG | CG | Qq | 7.559 | 0.0201 |
| YYJXLH116094102 | AG | CG | Qq | 8.062 | 0.0153 |
| YYJXLH117034903 | AA | CC | qq | 4.183 | 0.0081 |
| YYJXLH117055901 | AA | CC | qq | 5.573 | 0.0051 |

Note: Primers for amplification of tag SNP1 (A>G at 33,269,224 bp on chromosome 10 in Sscrofa10.2 and at 29,224,570 bp on chromosome 10 in Sscrofa11.1) are GAACTTGGCACAATGTAGGTGC (5’-3’) and TGGTCCTCTGGGTAAGTGGG (5’-3’). Primers for amplification of tag SNP2 (G>C at 33,282,602 bp on chromosome 10 in Sscrofa10.2 and at 29,238,784 bp on chromosome 10 in Sscrofa11.1) are AGTGGGTTTGATCCCTGTCC (5’-3’) and TCTGCTGTGTTGTGAGAAAGTT (5’-3’).

**Supplementary Table 6 Estimated breeding values for total number of piglets born (EBV_TNB) and *AHR* haplotypes of 224 Erhualian sows (see excel file “Table S6”)**

**Supplementary Table 7. Estimated breeding values for total number of piglets born (EBV_TNB) and *AHR* haplotypes of 344 French Large White sows (see excel file “Table S7”)**

**Supplementary Table 8. Primers for amplification of eight tag SNPs for identifying *KATNAL1* haplotypes**

|  | **Position(bp)^1^** | **Mutation** | **Forward** **primer (5’-3’)** | **Reverse** **primer (5’-3’)** |
| --- | --- | --- | --- | --- |
| SNP1 | 6,761,079 | T > C | GCTGCCCCATCAGAATAGGA | GGGGAGTATCTTCCGGCTTT |
| SNP2 | 6,761,456 | C > A | AATAGCGAAAGGCGAGTTCAG | CCAAAACCAGAGTATCGGGGG |
| SNP3 | 6,761,750 | C > G | TGGGTCCCTCATGTTCAACG | TGGTGTATTTTTAGGGACGCCT |
| SNP4 | 6,773,961 | G > A | ATAGCCTCCTGTTCACCACAC | AGGCAAGTCGCAGAGTCAAG |
| SNP5 | 6,775,977 | T > G | CCCTTGCCCTCCTAAGGGA | TCTTCAAGACGGGAGGGTTG |
| SNP6 | 6,779,936 | A > T | AGGAGGCGGTACTGAGAAGA | GCCTAAGTGCCTGACCAAGT |
| SNP7 | 6,796,248 | T > C | ACGGCTCTCACACCCATTTT | AGTTCCACCCCTAGCTGAGT |
| SNP8 | 6,800,363 | T > G | TGTGTTCTGCAGGTACAGGG | ACCAGTTCCTGCCCTCCTATC |

^1^ The positions of Ssrofa10.2. The corresponding positions of these eight SNPs on the pig genome assembly 11.1 are 7,005,720, 7,006,097, 7,006,391, 7,018,601, 7,020,626, 7,024,585, 7,040,895, 7,045,008 bp, respectively.

**Supplementary Table 9. *KATNAL1* haplotypes and fertility of 31 French Large White boars**

| **ID** | **Positions of tag SNPs on chromosome 11 (bp)** | | | | | | | | **Geno** | **No.** | **Mean TNB** | **Mean EBV** |
| --- | --- | --- | --- | --- | --- | --- | --- | --- | --- | --- | --- | --- |
|  | 6,761,079 | 6,761,456 | 6,761,750 | 6,773,961 | 6,775,977 | 6,779,936 | 6,796,248 | 6,800,363 |  |  |  |  |
| YYJXLH114017505 | T/T | A/A | G/G | A/A | T/T | T/T | C/C | G/G | QQ | 7 | 14.00 | 1.002 |
| YYJXLH115145907 | T/T | A/A | G/G | A/A | T/T | T/T | C/C | G/G | QQ | 8 | 13.63 | 0.414 |
| FRA29JEA1302883 | T/T | A/A | G/G | A/A | T/T | T/T | C/C | G/G | QQ | 6 | 14.50 | 0.345 |
| YYJXLH114049804 | T/T | A/A | G/G | A/A | T/T | T/T | C/C | G/G | QQ | 14 | 13.00 | 0.303 |
| YYJXLH114007801 | T/T | A/A | G/G | A/A | T/T | T/T | C/C | G/G | QQ | 5 | 12.80 | 0.271 |
| YYJXLH114008309 | T/T | A/A | G/G | A/A | T/T | T/T | C/C | G/G | QQ | 15 | 12.40 | 0.192 |
| FRA44C311303474 | T/T | A/A | G/G | A/A | T/T | T/T | C/C | G/G | QQ | 28 | 13.43 | 0.097 |
| FRA44C311303511 | T/T | A/A | G/G | A/A | T/T | T/T | C/C | G/G | QQ | 9 | 12.89 | 0.048 |
| FRA29JEA1303186 | T/T | A/A | G/G | A/A | T/T | T/T | C/C | G/G | QQ | 55 | 13.13 | 0.047 |
| FRA44C311303767 | T/T | A/A | G/G | A/A | T/T | T/T | C/C | G/G | QQ | 56 | 13.29 | 0.032 |
| YYJXLH115139306 | T/T | A/A | G/G | A/A | T/T | T/T | C/C | G/G | QQ | 6 | 12.33 | -0.124 |
| YYJXLH114019504 | T/T | A/A | G/G | A/A | T/T | T/T | C/C | G/G | QQ | 2 | 11.50 | -0.227 |
| FRA29JEA1303218 | T/T | A/A | G/G | A/A | T/T | T/T | C/C | G/G | QQ | 42 | 12.62 | -0.271 |
| YYJXLH114010310 | T/T | A/A | G/G | A/A | T/T | T/T | C/C | G/G | QQ | 6 | 11.67 | -0.277 |
| YYJXLH114018801 | T/T | A/A | G/G | A/A | T/T | T/T | C/C | G/G | QQ | 13 | 12.00 | -0.296 |
| FRA29JEA1302910 | T/T | A/A | G/G | A/A | T/T | T/T | C/C | G/G | QQ | 37 | 12.43 | -0.408 |
| FRA29JEA1303041 | T/T | A/A | G/G | A/A | T/T | T/T | C/C | G/G | QQ | 4 | 12.50 | -0.842 |
| YYJXLH114006513 | C/T | C/C | C/C | A/G | G/T | A/T | C/T | G/T | qq | 10 | 13.50 | 0.289 |
| FRA29JEA1303232 | T/T | C/C | C/C | A/A | T/T | A/T | C/T | G/T | qq | 27 | 14.19 | 0.279 |
| FRA29JEA1302869 | C/T | C/C | C/C | A/A | T/T | A/T | C/T | G/T | qq | 36 | 13.56 | 0.183 |
| FRA44C311303746 | C/T | C/C | C/C | A/A | T/T | A/T | C/T | G/T | qq | 39 | 13.41 | 0.058 |
| FRA44C311303820 | C/C | C/C | C/C | A/G | G/T | A/A | T/T | T/T | qq | 64 | 13.22 | 0.011 |
| FRA44C311303838 | C/T | C/C | C/C | A/A | T/T | A/T | C/T | G/T | qq | 46 | 13.52 | -0.010 |
| FRA44C311303879 | T/T | C/C | C/C | A/A | T/T | A/T | C/T | G/T | qq | 32 | 12.81 | -0.250 |
| YYJXLH114024601 | C/C | C/C | C/C | G/G | G/G | A/A | T/T | T/T | qq | 4 | 11.75 | -0.372 |
| YYJXLH114006712 | C/T | C/C | C/C | A/A | T/T | A/T | C/T | G/T | qq | 12 | 11.67 | -0.375 |
| FRA29JEA1303017 | C/T | C/C | C/C | A/A | T/T | A/T | C/T | G/T | qq | 25 | 12.60 | -0.509 |
| YYJXLH115155705 | T/T | C/C | C/C | A/A | T/T | A/T | C/T | G/T | qq | 6 | 10.67 | -0.616 |
| YYJXLH114016713 | C/T | C/C | C/C | A/G | G/T | A/T | C/T | G/T | qq | 15 | 11.27 | -0.716 |
| YYJXLH115014304 | C/T | C/C | C/C | A/A | T/T | A/T | C/T | G/T | qq | 1 | 11.00 | -0.916 |
| YYJXLH114007809 | T/T | C/C | G/G | A/A | T/T | T/T | C/C | G/G | qq | 2 | 9.50 | -1.342 |

**Geno,** genotypes; **No.,** the number of sows that mated with boars; **Mean** **TNB,** the mean total number of born piglets of sows that mated with boars; **Mean** **EBV**, the mean TNB_EBV of sows that mated with boars. The corresponding positions of these tag SNPs on the pig genome assembly 11.1 are 7,005,720, 7,006,097, 7,006,391, 7,018,601, 7,020,626, 7,024,585, 7,040,895, 7,045,008 bp, respectively.
