# Supplementary material for: Introgression of Eastern Chinese and Southern Chinese haplotypes contributes to the improvement of fertility and immunity in European modern pigs
Source: Gigascience. 2020 Mar 6;9(3):giaa014. doi: 10.1093/gigascience/giaa014 (PMC7059266; doi:10.1093/gigascience/giaa014)
Supplement: giaa014_Response_to_Reviewer_Comments_Original_Submission [file giaa014_response_to_reviewer_comments_original_submission.pdf]

Author's Response To Reviewer Comments

Close

Louis Bernatchez
Associate Editor, GigaScience
Oct 17, 2019
Dear Dr. Louis Bernatchez,

We are grateful to your and the reviewers' comments on our manuscript entitled "Population genomic data reveal origin and phenotypic effect of Chinese haplotypes introgressed into European modern pigs" (Manuscript ID: GIGA-D-19-00160). We greatly appreciate the opportunity to further revise this manuscript.

According to your suggestions, our article was polished by a professional editing company and we believe that the readability of the polished article has been greatly improved. In addition, we revised this paper on the basis of the two reviewers' comments. For details, please see our point-to-point response to these comments. All revisions are indicated in red in the manuscript. We have collected Authors' ORCIDs, including Hao Chen (0000-0002-5210-8924), Min Huang (0000-0001-9933-012X), Bin Yang (0000-0002-0689-0628), Zhongping Wu (0000-0001-5246-5190), Yong Hou (0000-0002-1351-9288) and Jun Ren (0000-0001-6664-3998). We did not develop new software and thus did not have RRIDs. According to your suggestion, we presented the 50-kb regions of introgression in supplementary tables 2 and 3.

We sincerely hope that the revised manuscript would satisfy you and the two reviewers and can be accepted for publication. Please do not hesitate to contact me if you have any other questions or comments.

Best regards,

Jun Ren
Reviewer #1
The paper describes interesting analyses on the origin and extend of Asian introgression into European commercial pig breeds. The amount of work is impressive and significantly contributes to our understanding of past introgression and persistence of haplotypes in European breeds. However, I had vaious questions about the methodology that I could not find the answer to in the paper. I also have some other issues that I think should be addressed before the manuscript can be considered for publication.
Response: Thank you for your positive comments. We have revised this paper on the basis of your comments.

The use of the English language is not always appropriate throughout the paper. Although it does not influence the overall readability and clarity of the message, I do think the manuscript will benefit from careful editing.
Response: According to this suggestion, our manuscript was polished by a professional editing company, LetPub (www.letpub.com).

The level of detail about the methodology is very inconsistent. Some analyses are described in much detail, whereas other analyses lack detail and cannot be reproduced. I think it is crucial to read through the methods again and add all details that will enable the readers to repeat the analysis. For example, details about how the haplotypes were constructed in the various haplotype plots using the pegas package should be provided. How many SNPs were used with which filtering, how was the phasing done, the distances calculated, etc.. Also, the results from the ALDER analyses should be shown somewhere (perhaps in the supplements?) because now it is too vague how the inference of 200-300 years was obtained.
Response: Thank you for your suggestion. We presented a more detailed description of the methodology in the revised manuscript. We also submitted all essential codes and files of the methodology to the

GigaDB temporary ftp site (ftp://user14@parrot.genomics.cn, username = user14, password = JunCNPig). For details, please see the readme text file. We provided details of the methods for haplotype reconstruction and estimation of admixture time. For your convenience, we show the relevant sentences as below:
"We also constructed haplotype networks using SNPs with a MAF of greater than 0.05 and call rates of greater than 90% at the GOLM1-NAA3 (298 SNPs), KATNAL1 (529 SNPs) and AHR (68 SNPs) loci. We explored the fastPhase function with 1000 iterations in Beagle v4.0 [39] to phase haplotypes and used the "haploNet" command in the R package "pegas" [40] to calculate the pairwise differences between haplotypes. We selected SNPs with an MAF of greater than 0.05, a call rate of greater than 90% and an LD (r2) < 0.3 using PLINK v1.9 [27], and then explored the selected SNPs to estimate the admixture time between populations via ALDER v1.0.3 under default parameters [13]. In short, we used the "convert" function in EIGENSTRAT [41] to convert the data format. We set FLW as a mixed population, EWB and SCN as one reference population, EWB and ECN as another reference population, and five years as one generation."

The reference genome assembly 10.2 was used, however the updated assembly 11.1 was produced in January 2017 already (manuscripthttps://www.biorxiv.org/content/10.1101/668921v1.). Especially the GOLM1-NAA35 and the AHR region occurred on multiple contigs and contained a gap nearby in 10.2, whereas 11.1 contains a single contig at that locus. I understand that redoing all the analyses on the new build requires a lot of work, but at some point we should move forward, especially if it improves the results. Perhaps a comparison could be made for specific loci?
Response: To address this concern, we mapped three representative regions including the GOLM1-NAA35, KATNAL1 and AHR loci to the Sscrofa11.1 reference genome assembly, and made a comparison of tag SNPs and interval positions at these loci on the two reference genome assemblies (10.2 vs. 11.1). For example, we present the positions of the three regions in both Sscrofa10.2 and Sscrofa11.1 in the main text. We also show the positions of tag SNPs for reconstructing GOLM1-NAA35 and KATNAL1 haplotypes in both Sscrofa10.2 and Sscrofa11.1 at the footnote of supplementary tables 6, 7, 8 and 9.

The rIBD in figure 2a seems to highly overestimate the proportion of Asia-derived haplotypes in the FLW. This seems impossible, given the admixture plot and PCA in figure 1. What were the settings for the fastIBD analysis? I could not find these in the methods. I assume the statement "We detected 5,107 and 5,024 50-kb regions with signatures of potential introgression from SCN or ECN pigs into FLW pigs, respectively" is therefore not correct, but these numbers rather reflect the bins in the far-right tail.
Response: As mentioned in the Method section, we conducted genome scans for regions of potential introgression using sliding windows of 50 kb with a step size of 25 kb. We calculated rIBD values for each window via IBD fragments that were iterated 10 times. As you assumed, the 5,107 and 5,024 50-kb regions are in the far-right tail of 5% empirical distribution. We defined these regions as the regions of potential introgression from SCN or ECN pigs into FLW pigs. Some of these regions are overlapped, corresponding to 4,803 and 4,719 non-overlapped regions, respectively. These regions are 240 Mb and 236 Mb in length, accounting for 8.9% and 8.7% of the total length of the pig genome (2.7G). The Asian fractions (8.9% and 8.7%) are roughly comparable to those in FLW as revealed by the ADMIXTURE analysis. Please see Figure 1d. Hence, the rIBD in figure 2a did not highly overestimate the proportion of Asia-derived haplotypes in the FLW.

Line 282: "with a strong introgression signal at the AHR locus (SSC9: 92.25-97.45 Mb)". I don't think this is a strong introgression signal, you have some peaks that stand out much stronger. What made you decide to focus on this locus?
Response: Boss et al. reported that the AHR region was one of the longest regions of introgression from Chinese pigs to Large White pigs, and an Asian-derived missense mutation in the AHR gene was significantly associated with litter size in Large White pigs (Nature Communication, 2014, doi: 10.1038/ncomms5392). Hence, we focused on this locus by using it as a proof of concept. We confirmed the significant association at this locus and then made a close examination on it, which enabled us to clarify the origin of the favorable haplotype for fertility.

Looking at the clustering, the sampled wild boar clearly represents the SCN lineage rather than ECN. Without this wild background leading to the ECN domestic lineage, it is impossible to assign the AHR haplotype to an external lineage such as the other Sus species.
Response: The clustering patterns indicate that the sampled wild boars (n = 10) have a closer genetic relationship with SCN pigs as compared to ECN pigs. Nevertheless, we did not think that these wild boars represent the SCN lineage. The ADMIXTURE analysis show that ECN and SCN pigs represent two ancient lineages of Chinese indigenous pigs when K = 3 to 4. The wild boars show a mixture of the two ancient lineages. Please see Figure 1d. In fact, two and four of the 10 sequenced wild boars were

sampled from Zhejiang province in Eastern China and Jiangxi province in Central China, respectively.

The clustering of one Chinese wild boar haplotype within the French Large white AHR haplotype group also suggests the occurrence of this haplotype in wild boar - although perhaps at low frequency.
Response: It is known that domestic pigs had recurrent hybridization with wild boars after domestication (Nature Genetics, 2015, doi: 0.1038/ng.3394). Given the very low frequency (1/20) of this haplotype in wild boars, we believe that the occurrence of this haplotype in wild boar is most likely due to gene flow between domestic pigs and wild boars.

You filtered out SNPs in 266 individuals with a MAF less than 0.01 -> this will remove many OUT-specific alleles. What would happen to the haplotype distances if you do include species-specific alleles, and how does that influence your conclusions?
Response: We agree with your opinion that a proportion of OUT-specific alleles will be removed under the filtering criteria of MAF less than 0.01. On the other side, this filtering criteria has been widely adopted to reduce the risk of sequencing errors especially when a limited number of samples were analyzed (Nature Genetics. doi:10.1038/s41588-018-0250-5 for example). We assume that haplotype distances between OUT and domestic pigs will be increased if we include species-specific alleles. When we analyzed the KATNAL1 locus, we did not include OUT haplotypes due to their low frequencies (Figure 4). To avoid potential bias, we removed OUT haplotypes at the GOLM1-NAA35 locus and present a new version of Figure 3 and Figure S6. From the two new figures, you can see that the deletion of the OUT haplotypes did not affect our result. At the AHR locus, the most frequent haplotype (XVI) appeared 100 times in all 266 sequenced individuals, including 30 FLW pigs, 24 other LW pigs, 18 EHL pigs and 26 in Tibetan pigs. This haplotype together with the OUT haplotype XII formed a major haplotype group, which was divergent from another two major haplotype groups from Eurasian wild boars and domestic pigs (fig. 5b). This pattern apparently conforms to the archaic introgression assumption. Altogether, our conclusion is not affected by filtering out SNPs with MAF of less than 0.01 in 266 individuals.

Regions of lower recombination can create outlier signals because of drift, this potential bias should be discussed
Response: We agree with this comment. Low recombination and genetic drift are two factors that cause reduced genetic variability at target regions, i.e. potential bias of selection signature.

The discussion section could better be read as some sort of conclusion, because lots of discussion is already provided in the results section (for example, line 211-221). I do like this structure (as in the discussion by section rather than discussion of all findings at the end) but it should be stated in the headers.
Response: Thank you for your suggestion. We added two headers for the two subsections of Discussion. One is entitled “Introgression of both SCN and ECN pig DNA contributed to the genetic improvement of European modern pig breeds” and another is entitled “Both naturally occurring interspecies hybridization and human-driven crossbreeding played important roles in the development of global pig breeds”.

Why is the highest peak, ATP5H, indicated in figure 2, but not discussed??
Response: As shown in Figure 2, the ATP5H region displayed the strongest signature of introgression from SCN pigs into FLW pigs. However, we did not detect significant selection signals in this region, neither between FLW and European wild boars (Figure 1a) nor between FLW and Large White pigs from other countries (Figure 1b) via the XP-EHH approach. This indicates that the ATP5H region did not experience directional selection after the introgression event and thus did not contribute to the selective breeding of FLW pigs. For this reason, we did not discuss about the ATP5H region.

Figure 1. Selection signals in the ATP5H region unraveled by the XP-EHH analysis. (a) Signals between FLW and European wild boars. (b) Signals between FLW and Large White pigs from other countries. The red dashed line indicates the 5% threshold line. The pink shaded area represents the ATP5H region.

Different coloration in figS12a and figS12b is confusing (the European wild).
Response: Corrected.

Why does one EWB cluster with the EUD in fig 1a?
Response: In fact, two European wild boars cluster with the EUD in an overlapped way in the PC1 and PC2 plots (Fig. 1a). The two wild boars were sampled from the Samos island of Greek. We assume that they had a considerable proportion of genetic components of the EUD due to gene flow as mentioned above. We further conducted PC3 and PC4 analyses for all sampled pigs. In the PC3 and PC4 plots, the two individuals cluster with other European wild boars. For details, please see the below figure. In

addition, the two individuals together with the other European wild boars define a branch separating from the EUD in the NJ tree (Fig 1b).

Figure 2. Principal component analysis (PCA) of Chinese and European pigs.

Was the t-test the most appropriate test for the haplotype association analysis? What about potential family relationships that could bias the outcome?
Response: We used EBV values of TNB as phenotypic traits for the association test at the KATNAL1 and AHR loci. The EBV values were calculated using the DMU software that treated family relationships, estrus, year, season, parity and pregnancy duration as fixed effects in the statistical model. For the association test at the GOLM1-NAA35 locus, we collected blood samples from unrelated Tibetan pigs that did not have common ancestors within three generations. Hence, we believe that potential family relationships did not bias the outcome.

How were the tagged SNPs for the haplotype association analyses selected?
Response: We explored the Beagle software to phase haplotypes at target loci and selected the least number of SNPs as tagged SNPs that can distinguish all haplotypes at the target loci through their genotypes. For instance, all KATNAL1 haplotypes in FLW pigs can be inferred using the genotypes of the eight tagged SNPs that we selected. For details, please see the figure shown below.

Figure 3. The eight tagged SNPs for phasing KATNAL1 haplotypes in FLW pigs.

Reviewer #2:
Chen et al. in their manuscript "Population genomic data reveal origin and phenotypic effect of Chinese haplotypes introgressed into European modern pigs" performed a thorough analysis explaining the introgression history of Chinese pigs from two distinc locations - Southern (SCN) and East (ECN) China - into an European breed, French Large White (FLW). The authors unravel introgression signatures from ECN and SCN and link them to immunity and reproductive traits respectively. They expand in two examples, the GOLM1-NAAR35 haplotype and the KATNAL1. Finally, they shed light in to the AHR intogressed haplotype introgressed form ECN pigs into Large-White pigs however showing that the AHR haplotype derived from ancient pig species. I consider this a very well written article of clear relevance to the field.
Response: We greatly appreciate your positive comments on our manuscript.

The authors mention Bosse et al. Nature Communications 2014 analysis as the pipeline used in their manuscript. In Bosse the authors use an independent method to verify gene flow between pig populations, namely D-statistics. Do the authors think that D-statistics in here would provide more robustness to their analysis?
Response: Yes, we agree that D-statistics would provide more robustness to our analysis. Bosse et al. also explored the rIBD approach to detect genomic signatures of introgression. We adopted this approach to uncover the introgression signals across the genome in this study. We further used complementary analyses including Fst, haplotype network and heatmap to obtain additional evidence for the introgression conclusion at the GOLM1-NAAR35, KATNAL1 and AHR loci

Page 6 Line 120: ECN pigs represented by Jinhua pigs and SCN pigs represented by Luchuan pigs appeared as the two ancestral lineages of Chinese pigs when K=3 (fig. 1d). Please consider adding … Jinghua pigs (JH) …
Response: Corrected.

Page 6 Line 129: We detected 5107 and 5024 50-Kb regions with signatures of potential introgression from SCN or ENC pigs into FLW pigs.To help follow-up studies it would be convenient to report an excel table with the set of detected regions with potential signatures of introgression, and not only the seven with "strong singatures of introgression" (supplementary Table 2).
Response: According to your suggestion, we present two supplementary tables that provide details for the 5170 (supplementary Table 2) and 5024 (supplementary Table 3) 50-kb regions with signatures of potential introgression from SCN or ENC pigs into FLW pigs.

Page 30 Line 704: Fig1. Population relationship and structure. (a) Neighbour-joining (NJ) tree based on an identity-by-state matrix among individuals. (b) Principal component analysis (PCA) of Chinese and European pigs. The figure shows in panel (a) the PCA analysis and in panel (b) the NJ tree.
Response: Thank you for your reminding. We have corrected this error.

Supplementary Figure 3. The colored dashed in the phylogenetic tree represent different genetic groups. Some clarifications or legends on that would be much appreciated, SCN, ECN, NCN…
Response: According to this comment, we have added clarifications for SCN, ECN, SWCN, NCN and EUD in the new version of Supplementary Figure 3.

Page 7 Line 36. SCN pigs and ECN pigs were enriched in the immune-related signalling and fertility pathways, respectively (fig. 2e). ECN genes are related to "positive regulation of dephosphorilation", is that related to fertility?
Response: Yes.

Page 15 Line 335. Better clarification on FLW pigs versus OUT animals were only 1.51 and 1.41, respectively (supplementary fig S13).
Response: Done. Please see the new version of Supplementary Figure 13

Supplementary Figure 12 is not mentioned in the text.
Response: Added.

Close
