# Supplementary material for: Introgression of Eastern Chinese and Southern Chinese haplotypes contributes to the improvement of fertility and immunity in European modern pigs
Source: Gigascience. 2020 Mar 6;9(3):giaa014. doi: 10.1093/gigascience/giaa014 (PMC7059266; doi:10.1093/gigascience/giaa014)
Supplement: giaa014_Response_to_Reviewer_Comments_Revision_1 [file giaa014_response_to_reviewer_comments_revision_1.pdf]

Author's Response To Reviewer Comments

Close

Dear Dr. Hongling Zhou,

Thank you for giving us the opportunity to further revise our manuscript entitled "Population genomic data reveal origin and phenotypic effect of Chinese haplotypes introgressed into European modern pigs" (GIGA-D-19-00160.R1). We are grateful to the reviewer's constructive comments that help us to improve this manuscript. According to the reviewer's suggestions, we explored a new data set retaining all informative SNPs to reanalyze the AHR region. The results are in agreement with the reviewer's assumption that interspecies hybridization most likely did not occur at this locus. For details, please see the point-to-point respond to the reviewer's comments. We revised the manuscript accordingly and now submit it to your journal. We sincerely hope that the revised manuscript would satisfy you and the reviewer. Your consideration of acceptance for publication will be greatly appreciated. Thanks again for your kind help and effort to our work. Please do not hesitate to contact me if you have any other questions or comments.

Best regards,
Jun Ren

Respond to Reviewers:

The manuscript has certainly improved compare to the previous version. I do like the overall paper and would like to see it published, however I feel some of my criticisms were not accurately dealt with. I will expand a bit on these to clarify what I meant and how they could help to improve the manuscript.

Respond: Thank you for your positive comment on the improvement of our manuscript. We greatly appreciate your further comments that enabled us to improve this paper.

-Looking at Figure 2a, the mean of the rIBD seems centered around 0. What I would conclude from that is that the majority of the genomes of FLW contains equal contributions from SCN and EWB, and indeed 'a positive rIBD indicated potential introgression' (line 486-487). I find it surprising that such a large part of the FLW genomes contains this high SCN signature. Looking at the different panels in Fig1 I don't see evidence of such high haplotype sharing. Could it be that the distribution is Z-transformed? If not, what would be your explanation of the rIBD value centered around 0 for figure 2a? Could you discuss this is the manuscript as well?

Respond: To address this concern, we carefully checked the statistical data and made a close examination on the introgression signals between FLW and SCN, and on those between FLW and EWB. Although the Manhattan plot (Figure 2a) looks like roughly equal contributions from SCN and EWB to FLW genomes, the frequency distribution of rIBD values clearly show that FLW contains a larger fraction of EWB genomes than SCN genomes. We show the distribution plot as a supplementary figure (supplementary figure S4a in the manuscript). In this figure, the median and mean of rIBD values were -0.023 and -0.026. It should be mentioned that the distribution was not Z-transformed.

-I am still not convinced that the introgression at the AHR locus is coming from interspecies hybridization. I feel the data does not support that conclusion. I have two reasons to doubt this statement:
1) The clustering of a Chinese wild boar within the haplotype group of FLW at the AHR locus. This can be seen in Fig 5a, 5b, Supp12. In your response you argue that this is probably due to introgression from domestic pigs into wild boar, but there is no evidence provided that this is more likely than the haplotype (or a similar one) being present (be it at low frequency) in the Chinese wild boars. Please note also that the sampling of Chinese wild boar is rather low, and represents multiple locations in the wild, spanning a large geographical area. Therefore, only low haplotype frequencies within this group would

be expected anyway.
2) Filtering for minor allele frequency >0.05 removed many OUT-specific alleles. If you remove all alleles that occur less than 25 times (when using 266 re-sequenced animals) the out-specific branch length is strongly reduced. This introduced a bias in your OUT animals towards ancestral alleles that are present in outgroup animals as well as in sus scrofa. In line 318 you mention that the nucleotide differences between the XVI haplotype and OUT haplotypes are only 7, but I would really like to know the differences without a filter for minor allele frequency. I find it highly unlikely that all out species contain the exact same haplotype at this locus, since they diverged millions of years ago. Therefore, I believe this is an artefact of the filtering. Even though not filtering for MAF may introduce some false positive variants within your dataset, those results can provide valuable information of how distinct these haplotypes really are. Also note that ABBA-BABA tests relyon an excess of derived lineage-specific alleles, and when these are filtered out such proportions are distorted.
I strongly suggest to redo the analysis at the AHR locus using a less stringent filtering on MAF, because of the unequal sampling in your dataset. Perhaps if you retain all alleles that are observed at least twice (so homozygous within one animal, or two heterozygotes) you already have a less biased view on the origin of the haplotypes at this locus. Your results indeed support Asian pig-derived haplotypes into FLW, and I think these results are worthwhile, but I would remove the conclusions about interspecies hybridization. If indeed interspecies hybridization occured before introgression into FLW, could you reconstruct a scenario how this should have happened? Was the introgression directly into the domestic lineage, or into a wild ancestor of ECN?

Respond: We are thankful to these constructive comments. According to your suggestions, we reanalyzed the AHR region using a new data set containing all SNPs that were observed at least twice in the 266 re-sequenced animals. The result is in agreement with your expectation. First, a number of OUT-specific alleles were added to this region (Figure 5a in the revision). Second, the most frequent haplotype (XVIII) appeared 99 times in the 266 sequenced individuals, including 30 FLW pigs, 24 Large White pigs from other countries, 17 Erhualian pigs, 26 Tibetan pigs and two Asian wild boars (Figure 5b in the revision). Last, the OUT-specific alleles increased the distance between this major haplotype and five OUT haplotypes XXVI, XXVII, XXVIII, IV and I from 7 to 11, 16 35, 31, 97, respectively (supplementary Figure 13 in the revision). Altogether, these findings support our conclusion of introgression of Asian (most likely East Chinese pigs) haplotypes into FLW pigs, but do not support our previous assumption of interspecies hybridization at the AHR locus. Hence, we removed the conclusion about interspecies hybridization from the manuscript and revised this manuscript accordingly. We highlight all corrections in red and show these new findings in the new version of Figure 5 and supplementary files in the revised manuscript.

Minor comment:
-In table S2 and S3 you have regions of potential introgression on chromosome 23, which doesn't exist in pigs. You probably mean the X-chromosome?

Respond: Yes. We have changed “23” to “X” in the two tables.

Close
