# Supplementary material for: Introgression of Eastern Chinese and Southern Chinese haplotypes contributes to the improvement of fertility and immunity in European modern pigs
Source: Gigascience. 2020 Mar 6;9(3):giaa014. doi: 10.1093/gigascience/giaa014 (PMC7059266; doi:10.1093/gigascience/giaa014)
Supplement: giaa014_Reviewer_1_Report_Original_Submission — Mirte Bosse -- 6/17/2019 Reviewed [file giaa014_reviewer_1_report_original_submission.pdf]

Reviewer Report

Title: Introgression of Eastern Chinese and Southern Chinese haplotypes contributes to the improvement of fertility and immunity in European modern pigs

Version: Original Submission **Date:** 6/17/2019

Reviewer name: Mirte Bosse

Reviewer Comments to Author:

The paper describes interesting analyses on the origin and extend of Asian introgression into European commercial pig breeds. The amount of work is impressive and significantly contributes to our understanding of past introgression and persistence of haplotypes in european breeds. However I had vaious questions about the methodology that I could not find the answer to in the paper. I also have some other issues that I think should be addressed before the manuscript can be considered for publication.

-The use of the English language is not always appropriate throughout the paper. Although it does not influence the overall readability and clarity of the message, I do think the manuscript will benefit from careful editing

-The level of detail about the methodology is very inconsistent. Some analyses are described in much detail, whereas other analyses lack detail and cannot be reproduced. I think it is crucial to read through the methods again and add all details that will enable the readers to repeat the analysis. For example, details about how the haplotypes were constructed in the various haplotype plots using the pegas package should be provided. How many SNPs were used with which filtering, how was the phasing done, the distances calculated, etc.. Also, the results from the ALDER analyses should be shown somewhere (perhaps in the supplements?) because now it is too vague how the inference of 200-300 years was obtained.

-The reference genome assembly 10.2 was used, however the updated assembly 11.1 was produced in January 2017 already (manuscript https://www.biorxiv.org/content/10.1101/668921v1.). Especially the GOLM1-NAA35 and the AHR region occurred on multiple contigs and contained a gap nearby in 10.2, whereas 11.1 contains a single contig at that locus. I understand that redoing all the analyses on the new build requires a lot of work, but at some point we should move forward, especially if it improves the results. Perhaps a comparison could be made for specific loci?

-The rIBD in figure 2a seems to highly overestimate the proportion of Asia-derived haplotypes in the FLW. This seems impossible, given the admixture plot and PCA in figure 1. What were the settings for the fastIBD analysis? I could not find these in the methods. I assume the statement "We detected 5,107 and 5,024 50-kb regions with signatures of potential introgression from SCN or ECN pigs into FLW pigs, respectively" is therefore not correct, but these numbers rather reflect the bins in the far-right tail.

Line 282: "with a strong introgression signal at the AHR locus (SSC9: 92.25-97.45 Mb)". I don't think this is a strong introgression signal, you have some peaks that stand out much stronger. What made you decide to focus on this locus?

I would be thrilled if the AHR locus is introgressed from another Sus species, but I am doubtful because

of the following:
-Looking at the clustering, the sampled wild boar clearly represent the SCN lineage rather than ECN. Without this wild background leading to the ECN domestic lineage, it is impossible to assign the AHR haplotype to an external lineage such as the other Sus species.
-The clustering of one chinese wild boar haplotype within the French Large white AHR haplotype group also suggests the occurence of this haplotype in wild boar - although perhaps at low frequency.
-You filtered out SNPs in 266 individuals with a MAF less than 0.01 -> this will remove many OUT-specific alleles. What would happen to the haplotype distances if you do include species-specific alleles, and how does that influence your conclusions?
-regions of lower recombination can create outlier signals because of drift, this potential bias should be discussed
Some other comments:
The discussion section could better be read as some sort of conclusion, because lots of discussion is already provided in the results section (for example, line 211-221). I do like this structure (as in ,the discussion by section rather than discussion of all findings at the end) but it should be stated in the headers.
Why is the highest peak, ATP5H, indicated in figure 2, but not discussed??
-different coloration in figS12a and figS12b is confusing (the European wild).
-why does one EWB cluster with the EUD in fig1a?
-Was the t-test the most appropriate test for the haplotype association analysis? What about potential family relationships that could bias the outcome?
-How were the tagged SNPs for the haplotype association analyses selected?

Methods

Are the methods appropriate to the aims of the study, are they well described, and are necessary controls included? Choose an item.

Conclusions

Are the conclusions adequately supported by the data shown? Choose an item.

Reporting Standards

Does the manuscript adhere to the journal's guidelines on minimum standards of reporting? Choose an item.

Choose an item.

Statistics

Are you able to assess all statistics in the manuscript, including the appropriateness of statistical tests used? Choose an item.

Quality of Written English

Please indicate the quality of language in the manuscript: Choose an item.

Declaration of Competing Interests

Please complete a declaration of competing interests, considering the following questions:

- Have you in the past five years received reimbursements, fees, funding, or salary from an organisation that may in any way gain or lose financially from the publication of this manuscript, either now or in the future?
- Do you hold any stocks or shares in an organisation that may in any way gain or lose financially from the publication of this manuscript, either now or in the future?
- Do you hold or are you currently applying for any patents relating to the content of the manuscript?
- Have you received reimbursements, fees, funding, or salary from an organization that holds or has applied for patents relating to the content of the manuscript?
- Do you have any other financial competing interests?
- Do you have any non-financial competing interests in relation to this paper?

If you can answer no to all of the above, write 'I declare that I have no competing interests' below. If your reply is yes to any, please give details below.

I declare that I have no competing interests

I agree to the open peer review policy of the journal. I understand that my name will be included on my report to the authors and, if the manuscript is accepted for publication, my named report including any attachments I upload will be posted on the website along with the authors' responses. I agree for my report to be made available under an Open Access Creative Commons CC-BY license (http://creativecommons.org/licenses/by/4.0/). I understand that any comments which I do not wish to be included in my named report can be included as confidential comments to the editors, which will not be published.

Choose an item.

To further support our reviewers, we have joined with Publons, where you can gain additional credit to further highlight your hard work (see: https://publons.com/journal/530/gigascience). On publication of this paper, your review will be automatically added to Publons, you can then choose whether or not to claim your Publons credit. I understand this statement.

Yes Choose an item.
