# Supplementary material for: Introgression of Eastern Chinese and Southern Chinese haplotypes contributes to the improvement of fertility and immunity in European modern pigs
Source: Gigascience. 2020 Mar 6;9(3):giaa014. doi: 10.1093/gigascience/giaa014 (PMC7059266; doi:10.1093/gigascience/giaa014)
Supplement: giaa014_Reviewer_1_Report_Revision_1 — Mirte Bosse -- 10/22/2019 Reviewed [file giaa014_reviewer_1_report_revision_1.pdf]

Reviewer Report

Title: Introgression of Eastern Chinese and Southern Chinese haplotypes contributes to the improvement of fertility and immunity in European modern pigs

Version: Revision 1 **Date:** 10/22/2019

Reviewer name: Mirte Bosse

Reviewer Comments to Author:

The manuscript has certainly improved compare to the previous version. I do like the overall paper and would like to see it published, however I feel some of my criticisms were not accurately dealt with. I will expand a bit on these to clarify what I meant and how they could help to improve the manuscript.
-Looking at Figure 2a, the mean of the rIBD seems centered around 0. What I would conclude from that is that the majority of the genomes of FLW contains equal contributions from SCN and EWB, and indeed 'a positive rIBD indicated potential introgression' (line 486-487). I find it surprising that such a large part of the FLW genomes contains this high SCN signature. Looking at the different panels in Fig1 I don't see evidence of such high haplotype sharing. Could it be that the distribution is Z-transformed? If not, what would be your explanation of the rIBD value centered around 0 for figure 2a? Could you discuss this is the manuscript as well?
-I am still not convinced that the introgression at the AHR locus is coming from interspecies hybridization. I feel the data does not support that conclusion. I have two reasons to doubt this statement:
1) The clustering of a Chinese wild boar within the haplotype group of FLW at the AHR locus. This can be seen in Fig 5a, 5b, Supp12. In your response you argue that this is probably due to introgression from domestic pigs into wild boar, but there is no evidence provided that this is more likely than the haplotype (or a similar one) being present (be it at low frequency) in the Chinese wild boars. Please note also that the sampling of Chinese wild boar is rather low, and represents multiple locations in the wild, spanning a large geographical area. Therefore, only low haplotype frequencies within this group would be expected anyway.
2) Filtering for minor allele frequency >0.05 removed many OUT-specific alleles. If you remove all alleles that occur less than 25 times (when using 266 re-sequenced animals) the out-specific branch length is strongly reduced. This introduced a bias in your OUT animals towards ancestral alleles that are present in outgroup animals as well as in sus scrofa. In line 318 you mention that the nucleotide differences between the XVI haplotype and OUT haplotypes are only 7, but I would really like to know the differences without a filter for minor allele frequency. I find it highly unlikely that all out species contain the exact same haplotype at this locus, since they diverged millions of years ago. Therefore, I believe this is an artefact of the filtering. Even though not filtering for MAF may introduce some false positive variants within your dataset, those results can provide valuable information of how distinct these haplotypes really are. Also note that ABBA-BABA tests rely on an excess of derived lineage-specific alleles, and when these are filtered out such proportions are distorted.
I strongly suggest to redo the analysis at the AHR locus using a less stringent filtering on MAF, because of

the unequal sampling in your dataset. Perhaps if you retain all alleles that are observed at least twice (so homozygous within one animal, or two heterozygotes) you already have a less biased view on the origin of the haplotypes at this locus.
Your results indeed support Asian pig-derived haplotypes into FLW, and I think these results are worthwhile, but I would remove the conclusions about interspecies hybridization.
If indeed interspecies hybridization occured before introgression into FLW, could you reconstruct a scenario how this should have happened? Was the introgression directly into the domestic lineage, or into a wild ancestor of ECN?
minor comment:
-In table S2 and S3 you have regions of potential introgression on chromosome 23, which doesn't exist in pigs. You probably mean the X-chromosome?

Methods

Are the methods appropriate to the aims of the study, are they well described, and are necessary controls included? Choose an item.

Conclusions

Are the conclusions adequately supported by the data shown? Choose an item.

Reporting Standards

Does the manuscript adhere to the journal's guidelines on [minimum standards of reporting?]() Choose an item.

Choose an item.

Statistics

Are you able to assess all statistics in the manuscript, including the appropriateness of statistical tests used? Choose an item.

Quality of Written English

Please indicate the quality of language in the manuscript: Choose an item.

Declaration of Competing Interests

Please complete a declaration of competing interests, considering the following questions:

- Have you in the past five years received reimbursements, fees, funding, or salary from an organisation that may in any way gain or lose financially from the publication of this manuscript, either now or in the future?

- Do you hold any stocks or shares in an organisation that may in any way gain or lose financially from the publication of this manuscript, either now or in the future?
- Do you hold or are you currently applying for any patents relating to the content of the manuscript?
- Have you received reimbursements, fees, funding, or salary from an organization that holds or has applied for patents relating to the content of the manuscript?
- Do you have any other financial competing interests?
- Do you have any non-financial competing interests in relation to this paper?

If you can answer no to all of the above, write 'I declare that I have no competing interests' below. If your reply is yes to any, please give details below.

I declare that I have no competing interests

I agree to the open peer review policy of the journal. I understand that my name will be included on my report to the authors and, if the manuscript is accepted for publication, my named report including any attachments I upload will be posted on the website along with the authors' responses. I agree for my report to be made available under an Open Access Creative Commons CC-BY license (http://creativecommons.org/licenses/by/4.0/). I understand that any comments which I do not wish to be included in my named report can be included as confidential comments to the editors, which will not be published.

Choose an item.

To further support our reviewers, we have joined with Publons, where you can gain additional credit to further highlight your hard work (see: https://publons.com/journal/530/gigascience). On publication of this paper, your review will be automatically added to Publons, you can then choose whether or not to claim your Publons credit. I understand this statement.

Yes Choose an item.
