# Supplementary material for: Introgression of Eastern Chinese and Southern Chinese haplotypes contributes to the improvement of fertility and immunity in European modern pigs
Source: Gigascience. 2020 Mar 6;9(3):giaa014. doi: 10.1093/gigascience/giaa014 (PMC7059266; doi:10.1093/gigascience/giaa014)
Supplement: giaa014_Reviewer_2_Report_Original_Submission — Marina Sanchez -- 8/19/2019 Reviewed [file giaa014_reviewer_2_report_original_submission.pdf]

Reviewer Report

Title: Introgression of Eastern Chinese and Southern Chinese haplotypes contributes to the improvement of fertility and immunity in European modern pigs

Version: Original Submission Date: 8/19/2019

Reviewer name: Marina Sanchez

Reviewer Comments to Author:

Chen et al. in their manuscript "Population genomic data reveal origin and phenotypic effect of Chinese haplotypes introgressed into European modern pigs" performed a thorough analysis explaining the introgression history of Chinese pigs from two distinc locations - Southern (SCN) and East (ECN) China - into an European breed, French Large White (FLW)
The authors unravel introgression signatures from ECN and SCN and link them to immunity and reproductive traits respectively. They expand in two examples, the GOLM1-NAAR35 haplotype and the KATNAL1. Finally, they shed light in to the AHR intogressed haplotype introgressed form ECN pigs into Large-White pigs however showing that the AHR haplotype derived from ancient pig species.
I consider this a very well written article of clear relevance to the field. I have the following minor comments:
Minor comments:
The authors mention Bosse et al. Nature Communications 2014 analysis as the pipeline used in their manuscript. In Bosse the authors use and independent method to verify gene flow between pig populations, namely D-statistics. Do the authors think that D-statistics in here would provide more robustness to their analysis
Page 6 Line 120: ECN pigs represented by Jinhua pigs and SCN pigs represented by Luchuan pigs appeared as the two ancestral lineages of Chinese pigs when K=3 (fig. 1d).
Please consider adding … Jinghua pigs (JH) …
Page 6 Line 129: We detected 5107 and 5024 50-Kb regions with signatures of potential introgression from SCN or ENC pigs into FLW pigs.
To help follow-up studies it would be convenient to report an excel table with the set of detected regions with potential signatures of introgression, and not only the seven with "strong singatures of introgression" (supplementary Table 2).
Page 30 Line 704: Fig1. Population relationship and structure. (a) Neighbour-joining (NJ) tree based on an identity-by-state matrix among individuals. (b) Principal component analysis (PCA) of Chinese and European pigs.
The figure shows in panel (a) the PCA analysis and in panel (b) the NJ tree.
Supplementary Figure 3. The colored dashed in the phylogenetic tree represent different genetic groups.
Some clarification or legend on that would be much appreciated, SCN, ECN, NCN…
Page 7 Line 36. SCN pigs and ECN pigs were enriched in the immune-related signalling and fertility pathways, respectively (fig. 2e).

ECN genes are related to "positive regulation of dephosphorilation", is that related to fertility?
Page 15 Line 335. Better clarification on FLW pigs versus OUT animals were only 1.51 and 1.41, respectively (supplementary fig S13.
Supplementary Figure 12 is not mentioned in the text.

Methods

Are the methods appropriate to the aims of the study, are they well described, and are necessary controls included? Choose an item.

Conclusions

Are the conclusions adequately supported by the data shown? Choose an item.

Reporting Standards

Does the manuscript adhere to the journal's guidelines on minimum standards of reporting? Choose an item.

Choose an item.

Statistics

Are you able to assess all statistics in the manuscript, including the appropriateness of statistical tests used? Choose an item.

Quality of Written English

Please indicate the quality of language in the manuscript: Choose an item.

Declaration of Competing Interests

Please complete a declaration of competing interests, considering the following questions:

- Have you in the past five years received reimbursements, fees, funding, or salary from an organisation that may in any way gain or lose financially from the publication of this manuscript, either now or in the future?
- Do you hold any stocks or shares in an organisation that may in any way gain or lose financially from the publication of this manuscript, either now or in the future?
- Do you hold or are you currently applying for any patents relating to the content of the manuscript?
- Have you received reimbursements, fees, funding, or salary from an organization that holds or has applied for patents relating to the content of the manuscript?
- Do you have any other financial competing interests?

- Do you have any non-financial competing interests in relation to this paper?

If you can answer no to all of the above, write 'I declare that I have no competing interests' below. If your reply is yes to any, please give details below.

I declare that I have no competing interests

I agree to the open peer review policy of the journal. I understand that my name will be included on my report to the authors and, if the manuscript is accepted for publication, my named report including any attachments I upload will be posted on the website along with the authors' responses. I agree for my report to be made available under an Open Access Creative Commons CC-BY license (http://creativecommons.org/licenses/by/4.0/). I understand that any comments which I do not wish to be included in my named report can be included as confidential comments to the editors, which will not be published.

Choose an item.

To further support our reviewers, we have joined with Publons, where you can gain additional credit to further highlight your hard work (see: https://publons.com/journal/530/gigascience). On publication of this paper, your review will be automatically added to Publons, you can then choose whether or not to claim your Publons credit. I understand this statement.

Yes Choose an item.
